# Changes in healthcare spending attributable to obesity and overweight: payer- and service-specific estimates

**DOI:** 10.1186/s12889-022-13176-y

**Published:** 2022-05-13

**Authors:** Eline van den Broek-Altenburg, Adam Atherly, Evon Holladay

**Affiliations:** 1 University of Vermont, The Larner College of Medicine, 89 Beaumont Ave., VT 05405 Burlington, USA; 2 University of Denver, Daniels College of Business, CO, Denver, USA

**Keywords:** Obesity, Healthcare spending, BMI, Quasi-experimental design, Cost model

## Abstract

**Background:**

National efforts to control US healthcare spending are potentially undermined by changes in patient characteristics, and in particular increases in rates of obesity and overweight. The objective of this study was to provide current estimates of the effect of obesity and overweight on healthcare spending overall, by service line and by payer using the National Institutes of Health classifications for BMI.

**Methods:**

We used a quasi-experimental design and analyzed the data using generalized linear models and two-part models to estimate obesity- and overweight-attributable spending. Data was drawn from the 2006 and 2016 Medical Expenditures Panel Survey. We identified individuals in the different BMI classes based on self-reported height and weight.

**Results:**

Total medical costs attributable to obesity rose to $126 billion per year by 2016, although the marginal cost of obesity declined for all obesity classes. The overall spending increase was due to an increase in obesity prevalence and a population shift to higher obesity classes. Obesity related spending between 2006 and 2016 was relatively constant due to decreases in inpatient spending, which were only partially offset by increases in outpatient spending.

**Conclusions:**

While total obesity related spending between 2006 and 2016 was relatively constant, by examining the effect of different obesity classes and overweight, it provides insight into spend for each level of obesity and overweight across service line and payer mix. Obesity class 2 and 3 were the main factors driving spending increases, suggesting that persons over BMI of 35 should be the focus for policies focused on controlling spending, such as prevention.

**Supplementary Information:**

The online version contains supplementary material available at 10.1186/s12889-022-13176-y.

## Background

Obesity has been identified as one of the key drivers of increased healthcare spending and reduced life expectancy in the United States [1–5] and worldwide [6]. Obesity has been linked to a multitude of health conditions, including coronary heart disease [7], chronic renal failure [8], many cancers, sleep apnea, gallbladder disease [9], Type 2 Diabetes [10] and other conditions. The link between obesity and chronic illness is the reason for the link between obesity and reduced life expectancy [3, 4].

There has also been an extensive investigation of the impact of obesity on healthcare spending. Obesity was identified as one of the key drivers of increased healthcare spending during the 1996–2006 time period [1], with the effect largely driven by increases in spending on chronic diseases caused by obesity [5]. More recent work has found that the proportion of spending attributable to obesity increased by 29% from 2001 to 2015, from 6.1 to 7.9% [11] with obese adults having higher inpatient and prescription drug spending, in particular [12]. The costs of obesity are higher in more obese individuals, both overall and for particular chronic illnesses, such as diabetes [13]. “Interesting, there is some evidence that the effect of obesity on total spending may have moderated in recent years, with a statistically insignificant decrease in total spending from 2010 to 2013, from $3748 [14]  to $3429.”

The more recent economic literature has begun measuring the effect of obesity by Body Mass Index (BMI) categories, mirroring the medical community. This is done using the National Institutes of Health (NIH) Body Mass Index (BMI) categories of overweight (BMI of 25–29.9), Class 1 (30–34.9), Class 2 (35–39.9) and Class 3 (Extreme) (BMI over 40). In clinical research, the new classification system has shown that decreases in life expectancy are concentrated in Class 3 [15]. There is limited evidence about whether healthcare spending is similarly concentrated in higher BMI classes, despite studies addressing BMI up to 45 [13, 16].

This study makes a number of new contributions to the existing literature on the effect of obesity and overweight on healthcare spending. First, we measure the effect of obesity and overweight on spending by service line (Emergency care, Inpatient and Outpatient) and payer using the NIH classifications for obesity and overweight; constant obesity related spending is largely due to a shift from inpatient care to outpatient care coupled with slight reductions in prescription drug spending. Previous service line and payer specific estimates used the more general obese / non-obese framework [17, 18], which may miss important nuances if the effect of obesity is concentrated in the higher categories [1]. Second, reforms in the Affordable Care Act (ACA) have shifted payer types, particularly through Medicaid expansions, which may have changed the distribution of payers from previous studies. Third, we examine the effect of different obesity classes and overweight on spending, by service line, to understand differences in how utilization occurs for different levels of obesity and overweight. Finally,we provide a careful examination of the suggestive evidence cited above that the effect of obesity may have moderated over more recent years. To do this, we analyze trends in obesity rates and obesity-induced spending between 2006 and 2016 and model the changes in spending for different BMI classes.

## Methods

Our data source is the Medical Expenditure Panel Survey (MEPS) Household Component, which collects detailed information regarding the use and payment for health care services from a nationally representative sample of Americans [19]. We used the 2006 and 2016 Full Year Consolidated file for our analyses. The MEPS data uses a consistent sampling frame over time and is a representative sample of the US non-institutionalized civilian population.

The MEPS sample included 34,655 observations for 2016 and 34,145 for 2006. The insurance categories are drawn from MEPS categorizations and are not mutually exclusive. To analyze the effect of obesity and overweight on healthcare spending, we looked at expenditures across service lines (total, inpatient, non-inpatient, and drugs), as well as by payer. We excluded everyone under the age of 18 and observations for whom we had no insurance or BMI information, which left us with 24,408 observations for 2016 and 22,989 for 2006.

In our empirical model, our dependent variables are healthcare expenditures, including total expenditures, inpatient, non-inpatient, and drugs expenditures. Non-inpatient is defined as outpatient and office-based expenditures. The main explanatory variable is BMI categories. BMI was used to create dummy variables for four BMI categories, overweight (BMI 25–29.9), BMI obesity class 1 (30–34.9), BMI obesity class 2 (35–39.9) and BMI obesity class 3 (extreme) (above 40). BMI was calculated based on self-reported height and weight. The BMI class “normal” (18.5–24.9) was the reference group in all models. Individuals with a BMI less than 18.5 were coded as “underweight”; underweight is controlled for in the model but not reported in the tables. The models controlled for sociodemographic and health characteristics that are not in the causal pathway between obesity and spending.

The control variables are drawn from the MEPS data, and include gender, race/ethnicity, smoking status, marital status, region of the country, education, and family income. Age was included and coded as a categorical variable for ages 18–34, 35–44, 45–54, 55–64, 65–74 and 75+. Expenditures were modelled using Generalized Linear Models (GLM) for total and non- inpatient expenditures; inpatient and drugs spending were modelled using two-part models (TPM) [20, 21] For all the expenditures classes, we performed a Modified Park test to identify the distribution of the expenditure data and the coefficient of the conditional variance function. The test supported the choice for GLM with gamma family and log link for all models. We used the Hosmer-Lemeshow test for goodness of fit. We calculated standard errors using bootstrap with 1000 iterations per model. Differences between coefficients were estimated using a standard t test. Observations with missing data for insurance (*n* = 265 for 2006 and 256 for 2016) or BMI (*n* = 741 and 701) were omitted from the analysis.

We also estimated the attributable fraction (AF) for obesity and overweight, which is equal to the ratio of the change in spending with and without obesity and overweight divided by total spending. The AF represents the proportion of spending attributable to the different BMI categories, controlling for other variables in the model. The estimated magnitude of the cost of obesity in previous work has varied considerably, perhaps driven by different study methodologies [22]. The advantage of using the AF methodology is that the estimates can be updated periodically to track the cost effect of BMI. This approach has been previously used in obesity as well smoking [23] and falls in older adults [24, 25]. Standard errors were calculated using a bootstrap method with 200 replications. We used STATA 15 for all analysis. Expenditure numbers from 2006 were adjusted to 2016 prices using the gross domestic product implicit price deflator (GDP deflator) from the Bureau of Economic Analysis [26]. The general price deflator was preferred to allow for differences in the social value of healthcare interventions [27].

## Results

We first estimated the marginal effect of obesity and overweight (in dollars), by BMI category, on overall healthcare spending (Table 1).

**Table 1:** Increase in adult per capita total spending attributable to obesity and overweight in 2016 and 2006

Year	2016		2006	
**BMI class**	**Mean spending difference compared to normal weight ($)**	**Proportion of population (weighted) (%)**	**Mean spending difference compared to normal weight ($)**	**Proportion of population (weighted) (%)**
***Overweight***	367.11 [251.966]	33.8	321.39* [189.187]	34.6
***Obese 1***	1028.65 *** [301.020]	18.3	1482.45*** [238.620]	17.1
***Obese 2***	1803.94 *** [412.280]	8.0	2165.40*** [348.764]	6.3
***Obese 3***	2718.67 *** [508.660]	5.0	3002.72*** [434.515]	3.8

*p < 0.10 ** *p* < 0.05 ***p < 0.01

Marginal effects, standard errors between []

This marginal effect represents the mean association of spending with obesity and overweight, controlling for other factors. The largest difference in spending was for the Obese 3 class; individuals who have Class 3 Obesity spent an average of $2719 more per person per year in 2016 than those in a normal weight class. This is significantly higher than those in Obese 2, who spent an average of $1804 more per person per year, and Obese 1, where mean spending was $1029 per person per year. The increase in healthcare spending in Class 3 is problematic because the proportion of individuals in Class 3 has increased by 31.5% between 2006 and 2016 (from 3.8 to 5%). Surprisingly, the marginal effect was smaller in 2016 than 2006 for all obesity classes, after adjusting for inflation. The largest decline was for Obese 3, which declined from 10.5% from $3003 in 2006 to $2719 in 2016. This same trend was found for Obese 2, which went decreased16,67% from $2165 to $1804 and Obese 1, which decreased 30.6% from $1482 to $1029. Individuals in the overweight category were marginally significantly different (*p* < 0.1) from the reference group only for 2006, the estimated coefficient for 2016 was similar to that in 2006. This time trend varies across payers (Table 2).

**Table 2:** Increase in adult per capita total spending attributable to obesity and overweight in 2016 and 2006, by payer

Insurance category	BMI category	Mean spending difference compared to normal weight ($) 2016	Mean spending difference compared to normal weight ($) 2006
**Medicare**	*Overweight*	700.12 [615.527]	− 396.71 [645.569]
	*Obese 1*	1759.05*** [718.867]	1587.90** [803.166]
	*Obese 2*	2881.70*** [1018.698]	2189.19** [1140.802]
	*Obese 3*	3774.82*** [1213.880]	6615.03*** [1540.392]
**Medicaid**	*Overweight*	774.95 [755.025]	306.68 [766.943]
	*Obese 1*	662.26 [847.869]	1121.38 [871.873]
	*Obese 2*	1541.40 [1105.689]	2621.23** [1163.954]
	*Obese 3*	2953.68 *** [1199.789]	4324.89*** [1184.332]
**Private**	*Overweight*	189.26 [304.958]	486.80 ** [249.337]
	*Obese 1*	899.426 *** [369.729]	1633.36 *** [315.903]
	*Obese 2*	1634.05 *** [506.560]	2461,27 *** [466.720]
	*Obese 3*	2614.93 *** [651.227]	2787.39 *** [599.565]

*p < 0.10 ** *p* < 0.05 ***p < 0.01

Marginal effects, standard errors between []

For Medicare, the costs for Obese 3 declined markedly from 2006 to 2016 ($6615 to $3775), while increasing for Obese 2 ($2189 to $2882) and Obese 1 ($1588 to $1759). Medicaid exhibited a similar trend for Obese 3, with a large decrease in the marginal cost ($4325 to $2954). Medicaid also showed decreases for Obese 2 ($2621 to $1541) and Obese 1 ($1121 to $662), but the 2016 Obese 2 coefficient and both Obese 1 coefficients were statistically insignificant. For private insurance, spending went down for all three BMI classes, with the largest decrease for Obese 2 ($2461 to $1634)

Being overweight had no effect on spending overall. Although 33% of the population was overweight in 2016, the marginal effect overall (Table 1) and by payer (Table 2) was insignificantly different from zero for all models except private insurance in 2006.

The reason for these trends is suggested by Table 3. Inpatient spending for Obese 3 declined 35% from $1110 to $727 from 2006 to 2016 and inpatient spending for Obese 2 declined 29% from $679 to $483. Inpatient spending for Obese 1 increased between 2006 and 2016 from $241 to $343. In contrast, non-inpatient spending increased for BMI classes 3 and 2, with an increase for Obesity 3 from $714 in 2006 to $869 in 2016, and an increase for Obese 2 from $592 to $751.

**Table 3:** Increase in adult per capita total spending attributable to obesity and overweight in 2016 and 2006, by service line

Type of Service	Obesity category	Mean spending difference compared to normal weight ($) 2016	Mean spending difference compared to normal weight ($) 2006
**Inpatient**	*Overweight*	239.01 [168.218]	9.81 [108.656]
	*Obese 1*	353.49 ** [172.540]	241.42 * [141.323]
	*Obese 2*	483.46 ** [235.758]	679.46 *** [177.842]
	*Obese 3*	727.23 *** [251.747]	1110.10 *** [209.555]
**Non-inpatient**	*Overweight*	220.57 ** [103.852]	7.54 [69.645]
	*Obese 1*	396.07 *** [124.030]	435.29 *** [87.427]
	*Obese 2*	751.34 *** [169.683]	592.21 *** [126.827]
	*Obese 3*	868.66 *** [208.378]	714.17 *** [156.638]
**Rx**	*Overweight*	−27.82 [118.381]	181.226 *** [51.697]
	*Obese 1*	379.05 *** [125.496]	643.61 *** [150.279]
	*Obese 2*	632.33 *** [160.973]	671.49 *** [72.626]
	*Obese 3*	1045.79 *** [145.211]	1031.44 *** [86.354]

*p < 0.10 ** *p* < 0.05 ***p < 0.01

Marginal effects, standard errors between []

Meanwhile, there was a small decrease for Obese 1 in non-inpatient spending. Prescription drug spending was relatively flat for Obese 3 and Obese 2 but declined from $643 to $379 for Obese 1.

Table 3 suggests a shift in spending for obesity. For Obese 3, the most expensive spending category in 2006 was inpatient spending ($1110), followed by prescription drugs ($1031) and non-inpatient spending ($714). In contrast, the top expense in 2016 was for prescription drugs ($1046), with inpatient spending third ($727). Obese 2 showed the same general pattern: a very slight decline in drug spending, an increase in non-inpatient spending and a decrease in inpatient spending.

Overall, changes in the attributable fraction of healthcare spending varied depending on the service line and BMI category (Table 4). For inpatient care, the attributable fraction declined for Obese 2 (3.2 to 1.9%) and Obese 3 (3.9 to 2.5%) but increased for Obese 1 (2.4 to 3.5%). Non-inpatient and prescription drug spending had exactly the opposite pattern, with the attributable fraction decreasing for Obese 1 (5.6 to 4.0%) while increasing for Obese 2 (3.0 to 3.5%) and Obese 3 (2.2 to 2.5%). Finally, the attributable fraction for prescription drug spending decreased for both Obese 1 (7.6 to 5.3%) and Obese 2 (4.3 to 4.1%) and increased slightly for Obese 3 (4.2 to 4.3%).

**Table 4:** Aggregate total medical spending attributable to levels of obesity and overweight, by service line^a^

	Service Line	Overweight	Obese 1	Obese 2	Obese 3
**2016**	Inpatient	3.9 (3.17) 12.4	3.5 (2.08) 10.9	1.9 (1.20) 5.9	2.5 (0.88) 7.8
	Non-Inpatient	3.9 (2.05) 16.7	4.0 (1.42) 17.4	3.5 (1.01) 15.0	2.5 (0.62) 10.5
	Rx	−1.2 (2.05) -4.2	5.3 (1.56) 18.0	4.1 (0.99) 14.1	4.3 (0.58) 14.9
**2006**	Inpatient	1.8 (3.33) 5.1	2.4 (1.68) 6.8	3.2 (1.00) 9.1	3.9 (1.09) 11.2
	Non-inpatient	0.2 (1.70) 0.5	5.6 (1.35) 16.9	3.0 (0.84) 9.2	2.2 (0.48) 6.8
	Rx	5.1 (1.19) 12.2	7.6 (1.28) 18.3	4.3 (0.43) 10.2	4.2 (0.44) 10.0

Attributable fraction (%) and Total spending ($ million). Standard error in parentheses

^a^The Totals were calculated by adding up the columns. We also calculated AF and Total Spending independently and found similar results

Actual spending increased for prescription drugs and non-inpatient care for Obese 3, with a nearly $5B increase in prescription drug spending alone. Spending in Obese 2 had the largest overall increase, with an increase of nearly $4B in prescription drugs ($10.2B to $14.1B) and $6B in non-inpatient care. Spending for Obese 1 was largely flat for prescription drugs and non- inpatient care. Inpatient spending declined for Obesity Class 2 ($3.2B) and Obesity Class 3 ($3.4B) but increased for Obese Class 1 ($4.1B).

The effect by payer varied (Table 5). Medicare experienced an increase in attributable fraction for Obese 1 (from 3.1% in 2006 to 3.6% in 2016) and Obese 2 (1.8 to 2.4%) and a decline for Obese 3 (from 4.0 to 2.3%). Medicaid also experienced an increase in attributable fraction for Obese 1 (from 2.7 to 3.5%), while private insurance saw a decrease for that same class (from 7.0 to 3.6%). Both Medicaid and private insurance saw deceases in the attributable fraction for Obese 2 (3.8 to 2.2% and 4.2 to 2.9%, respectively).

**Table 5:** Aggregate total medical spending attributable to levels of obesity and overweight, by payer

	Insurance Category	Overweight	Obese 1	Obese 2	Obese 3	TOTAL ($ billion)
**2016**	Medicare	2.3 (2.58) 12.0	3.6 (1.84) 18.8	2.4 (1.16) 12,5	2.3 (0.13) 12.3	
	Medicaid	3.5 (4.31) 6.6	1.9 (2.67) 3.5	2.2 (1.72) 4.2	4.3 (1.50) 8.0	
	Private	1.3 (2.38) 9.4	3.6 (1.33) 25.9	2.9 (0.92) 21.0	2.8 (0.74) 19.9	
			**48.2**	**37.7**	**40.2**	**126.1**
**2006**	Medicare	−1.4 (2.32) -5.1	3.1 (1.42) 11.1	1.8 (0.79) 6.2	4.0 (1.30) 14.1	
	Medicaid	(2.85) 1.1	2.7 (2.37) 2.7	3.8 (2.47) 3.9	6.8 (2.63) 7.0	
	Private	3.7 (1.78) 21.0	7.0 (1.70) 40.1	4.2 (0.86) 24.2	2.6 (0.53) 15.0	
			**53.9**	**34.3**	**36.1**	**124.3**

Attributable fraction (%) and Total spending ($ billion). Standard error in parentheses

Row totals exclude Overweight, to calculate obesity total spending

The pattern for Obese 3 was similar, with declines for Medicaid (6.8 to 4.3%) and private insurance largely unchanged (2.6 to 2.8%). Overall spending for Obese 1 decreased from $53.9 billion to $48.2 billion, while it increased for Obese 2 from $34.3B to $37.7B and for Obese 3 from $36.1B to $40.2B. Total spending increased from $124.3B to 126.1B, even though spending in Obesity 1, the largest group, declined. The largest increase is in Obese 3 (from 36.1B to 40.2B), an increase that nearly matched the decrease in spending Obesity 1, despite the Obese 1 group being more than three times as large.

## Discussion

In this paper we find that spending associated with obesity and overweight has changed in some important ways over the past 10 years. First, we show that the spending on obesity is increasingly focused on individuals in Obesity Class 3 (Extreme). These individuals are 5% of the total population and only about one in six obese persons fall into this class. Yet more than a quarter of obesity related costs (26.1%) are concentrated in this group. And this is the group that is proportionately growing the fastest, with a 32% increase over the past decade.

For other obesity classes and overweight, spending has been more effectively controlled and total spending has been relatively flat. The models separating the effect of changes in obesity prevalence and the relationship of obesity and spending indicate that the latter is the reason for the moderation in effect. This is largely due to a shift from inpatient care to outpatient care coupled with slight reductions in prescription drug spending. Also, despite the coverage expansions in the ACA, the majority of spending remains paid for by private insurance ($67B), rather than Medicare ($43B) or Medicaid ($16B). The higher spending in private insurance reflects the higher number of individuals with coverage of that type. Spending for overweight persons is insignificantly different from normal weight spending, which may suggest a lost opportunity to intervene.

There are a number of limitations to this study. First, the analysis is based on MEPS data. Other data sources may have different spending numbers, particularly due to the inclusion or exclusion of long-term care spending. The advantage of MEPS is its widespread usage as a measure of healthcare spending, which allows comparisons to other studies. Second, our data is based on self-report height and weight as there are no nationally representative data set that includes both measured height/weight and annual medical spending [2]. Previous research concluded that reporting error in weight can lead to bias in estimates of the healthcare consequences of obesity and the extent of underreporting increases with measured weight [28]. Endogeneity is also possible in this study if there are unobserved characteristics associated with both insurance and obesity. “For example, unobserved socioeconomic status could be correlated with obesity creating bias in the obesity coefficient.” Comparisons across different studies should be done with caution because of differences in model design, sampling frame and control variables. Caution should be used in comparing differences between payers, given that patients with different arrangements face very different prices, which may lead to differences in demand or access for a given level of health. For example, individuals in Medicaid may struggle to access primary care which could lead to relatively lower outpatient spending and higher inpatient spending. We do not include the uninsured in our study because our focus is on changes by payer. Future research should examine how obesity and overweight affects the uninsured.

We stratify the analysis by age and insurance status to be able to compare results to comparable work done with earlier years, so time trends could be established. It would have been interesting to stratify by gender as well, but it is difficult to treat the gender issue carefully without providing significant additional context and result tables. Finally, the interplay between age, obesity or overweight and chronic illness may become more important as people who became obese at a younger age enter the Medicare program.

## Conclusions

Overall, we find that the obesity attributable fraction of healthcare spending has actually declined over the past decade, despite increased obesity and overweight prevalence. Our results suggest this success is due to the shift from inpatient to outpatient settings for care. These findings suggest there are two potential conclusions that may be drawn from this. First, obesity has not been the key driver of increases in healthcare spending over the past decade. Obesity related spending has increased, but other spending (the denominator) has increased more quickly. To understand why costs have increased over the past decade, analysts need to look for other culprits.

Second, obesity may be a more important cost driver in the next decade. The proportion of the population which is obese is increasing. Over the past decade, the increased prevalence was offset with changes in the pattern of spending – inpatient to non-inpatient – which moderated the increase. Without further reductions in per capita spending, the effect of increases in the proportion of the population which is obese may have a larger effect on healthcare spending. This is particularly true because of the increase in extreme obesity. Future efforts to control obesity- related spending are likely to be most impactful if they concentrate on individuals with BMI over 40 as well as preventing individuals from progressing to high levels of obesity.

## Supplementary Information

**Additional file 1.** 2006 Full Regression Results.

**Additional file 2.** 2016 Full regression Results.

## Data Availability

The datasets generated and/or analyzed during the current study are available at https://www.meps.ahrq.gov/mepsweb/data_stats/data_overview.jsp

## Abbreviations

ACA: Affordable Care Act
AF: Attributable Fraction
BMI: Body Mass Index
GDP: Gross Domestic Product
GLM: Generalized Linear Models
MEPS: Medical Expenditure Panel Survey
NIH: National Institutes of Health
TPM: Two-Part Models

## References

[1] Finkelstein EA, Fiebelkorn IC, Wang G (2003). National Medical Spending Attributable to Overweight and Obesity: how much, and Who's paying? Further evidence that overweight and obesity are contributing to the nation's health care bill at a growing rate. Health Aff.

[2] Finkelstein EA, Trogdon JG, Cohen JW, Dietz W (2009). Annual medical spending attributable to obesity: payer- and service-specific estimates. Health Aff.

[3] Fontaine KR, Redden DT, Wang C, Westfall AO, Allison DB. Years of life lost due to obesity. JAMA. 2003;289(2):187–93. 10.1001/jama.289.2.187 12517229

[4] Peeters A, Barendregt J, Willekens F, Mackenbach J, Al Mamun A, Bonneux L. Obesity in adulthood and its consequences for life expectancy: a life-table analysis. Ann Intern Med. 2003. 10.7326/0003-4819-138-1-200301070-00008 12513041

[5] Thorpe KE, Florence CS, Howard DH, Joski P (2004). The impact of obesity on rising medical spending: higher spending for obese patients is mainly attributable to treatment for diabetes and hypertension. Health Aff.

[6] Collaborators GO (2017). Health effects of overweight and obesity in 195 countries over 25 years. New England J Med.

[7] Manson JE, Colditz GA, Stampfer MJ, Willett WC, Rosner B, Monson RR (1990). A prospective study of obesity and risk of coronary heart disease in women. New England J Med.

[8] Ejerblad E, Fored CM, Lindblad P, Fryzek J, McLaughlin JK, Nyrén O (2006). Obesity and risk for chronic renal failure. J Am Soc Nephrol.

[9] Pi-Sunyer FX (1993). Medical hazards of obesity. Ann Int Med.

[10] Meigs JB, Wilson PW, Fox CS, Vasan RS, Nathan DM, Sullivan LM (2006). Body mass index, metabolic syndrome, and risk of type 2 diabetes or cardiovascular disease. J Clin Endocrinol Metab.

[11] Biener A, Cawley J, Meyerhoefer C (2018). The impact of obesity on medical care costs and labor market outcomes in the US. Clin Chem.

[12] Biener AI, Decker SL (2018). Medical care use and expenditures associated with adult obesity in the United States. JAMA.

[13] Cawley J, Meyerhoefer C, Biener A, Hammer M, Wintfeld N (2015). Savings in medical expenditures associated with reductions in body mass index among US adults with obesity, by diabetes status. Pharmacoeconomics.

[14] Biener A, Cawley J, Meyerhoefer C. The high and rising costs of obesity to the US health care system: Springer; 2017. 10.1007/s11606-016-3968-8 PMC5359159 28271429

[15] Preston SH, Stokes A (2011). Contribution of obesity to international differences in life expectancy. Am J Public Health.

[16] Wang YC, Pamplin J, Long MW, Ward ZJ, Gortmaker SL, Andreyeva T (2015). Severe obesity in adults cost state Medicaid programs nearly $8 billion in 2013. Health Aff.

[17] Bell K, Salmon A, McNaughton D (2011). Alcohol, tobacco, obesity and the new public health.

[18] Cawley J, Meyerhoefer C (2012). The medical care costs of obesity: an instrumental variables approach. J Health Econ.

[19] Cohen JW, Monheit AC, Beauregard KM, Cohen SB, Lefkowitz DC, Potter DE (1996). The medical expenditure panel survey: a national health information resource. Inquiry.

[20] Belotti F, Deb P, Manning WG, Norton EC (2015). Twopm: two-part models. Stata J.

[21] Norton EC, Dow WH, Do YK (2008). Specification tests for the sample selection and two-part models. Health Serv Outcome Res Methodol.

[22] Kim DD, Basu A (2016). Estimating the medical care costs of obesity in the United States: systematic review, meta-analysis, and empirical analysis. Value Health.

[23] Xu X, Bishop EE, Kennedy SM, Simpson SA, Pechacek TF (2015). Annual healthcare spending attributable to cigarette smoking: an update. Am J Prevent Med.

[24] Florence CS, Bergen G, Atherly A, Burns E, Stevens J, Drake C (2018). Medical costs of fatal and nonfatal falls in older adults. J Am Geriatr Soc.

[25] Haddad YK, Bergen G, Florence C (2019). Estimating the economic burden related to older adult falls by state. J Public Health Manage Pract.

[26] Bureau of Economic Analysis USDoC. GDP Price Deflator. 2019.

[27] Dunn A, Grosse SD, Zuvekas SH (2018). Adjusting health expenditures for inflation: a review of measures for health services research in the United States. Health Serv Res.

[28] Cawley J, Maclean JC, Hammer M, Wintfeld N (2015). Reporting error in weight and its implications for bias in economic models. Econ Hum Biol.

