# Supplementary material for: Changes in healthcare spending attributable to obesity and overweight: payer- and service-specific estimates
Source: BMC Public Health. 2022 May 13;22:962. doi: 10.1186/s12889-022-13176-y (PMC9101934; doi:10.1186/s12889-022-13176-y)
Supplement: Supplementary file 1 — Additional file 1. 2006 Full Regression Results. [file 12889_2022_13176_MOESM1_ESM.pdf]

	(1)	(2)
	outpatient	
	Coef./std.errors	Coef./std.errors
main		
overweight	0.0142	
	(0.0959)	
obese	0.3695***	
	(0.1111)	
unins	-0.9198***	
	(0.1014)	
medicaid	0.4907***	
	(0.1732)	
medicare	0.4293	
	(0.5197)	
female	0.9185***	
	(0.0865)	
smoker	0.2206**	
	(0.1059)	
married	0.2741***	
	(0.0909)	
black	-0.0944	
	(0.1577)	
amind	-0.2763	
	(0.2246)	
asian	0.3910***	
	(0.1203)	
othrace	0.0000	
	(.)	
midwest	-0.0658	
	(0.1328)	
south	-0.1722	
	(0.1236)	
west	-0.1135	
	(0.1354)	
lesshischool	-0.0153	
	(0.1213)	
collegeplus	0.2936***	
	(0.1037)	
poor	0.1079	
	(0.1570)	
nearpoor	0.3025	
	(0.2235)	
middleinc	0.0277	
	(0.1330)	
highinc	0.1184	
	(0.1426)	

_bs_1		2.9852e+09***
		(1.0234e+09)
_bs_2		0.0847***
		(0.0277)
_cons	5.2827***	
	(0.2116)	
No. of Obs.	5848.0000	5848.0000
R-Squared		

	(1)	(2)
	inpatient	
	Coef./std.errors	Coef./std.errors
main		
overweight	0.3188**	
	(0.1274)	
obese	0.3504***	
	(0.1290)	
unins	-0.4889***	
	(0.1501)	
medicaid	0.6736***	
	(0.1591)	
medicare	0.7689*	
	(0.4022)	
female	1.7721***	
	(0.1527)	
smoker	-0.1062	
	(0.1398)	
married	0.6839***	
	(0.1102)	
black	-0.1335	
	(0.1670)	
amind	0.1889	
	(0.3108)	
asian	0.0560	
	(0.1408)	
othrace	0.0000	
	(.)	
midwest	0.1316	
	(0.1928)	
south	0.1987	
	(0.1753)	
west	-0.1588	
	(0.1868)	
lesshischool	-0.1058	
	(0.1345)	
collegeplus	-0.0341	
	(0.1338)	
poor	0.5598***	
	(0.1603)	
nearpoor	0.0143	
	(0.2299)	
middleinc	-0.1078	
	(0.1687)	
highinc	-0.3854*	
	(0.1997)	

_bs_1		3.1721e+09**
		(1.4084e+09)
_bs_2		0.0944**
		(0.0405)
_cons	-4.3375***	
	(0.2869)	
glm		
overweight	852.8192	
	(1269.4494)	
obese	877.0597	
	(1272.4061)	
unins	-3793.8168**	
	(1608.6482)	
medicaid	-1825.1314	
	(1545.4380)	
medicare	7227.2773**	
	(3663.3823)	
female	-5159.5896***	
	(1658.6007)	
smoker	70.3581	
	(1487.6374)	
married	434.1625	
	(1198.9775)	
black	3143.6703*	
	(1671.1615)	
amind	-1114.6730	
	(3208.3186)	
asian	1187.6286	
	(1461.1929)	
othrace	0.0000	
	(.)	
midwest	-1634.2903	
	(1939.3854)	
south	-1551.9261	
	(1782.3810)	
west	33.6626	
	(1869.5516)	
lesshischool	2083.3418	
	(1329.2047)	
collegeplus	-1174.9257	
	(1336.6689)	
poor	-80.9104	
	(1624.0386)	
nearpoor	5930.7125**	
	(2334.2632)	
middleinc	2261.3042	

	(1715.7756)	
highinc	4550.3952**	
	(2008.2560)	
_cons	11407.3418***	
	(2746.0026)	
No. of Obs.	5848.0000	5848.0000
R-Squared		

	(1)	(2)
	inpatient	
	Coef./std.errors	Coef./std.errors
main		
overweight	0.2002	
	(0.1989)	
obese	0.8319***	
	(0.1812)	
unins	-0.2825	
	(0.2083)	
medicaid	0.4012*	
	(0.2369)	
medicare	1.2547***	
	(0.2935)	
female	0.7683***	
	(0.1601)	
smoker	0.1365	
	(0.1778)	
married	0.0640	
	(0.1637)	
black	0.2621	
	(0.2566)	
amind	0.7167**	
	(0.3598)	
asian	0.3627*	
	(0.2166)	
othrace	0.0000	
	(.)	
midwest	0.3720	
	(0.2382)	
south	-0.0164	
	(0.2315)	
west	-0.0246	
	(0.2470)	
lesshischool	0.2243	
	(0.2159)	
collegeplus	0.1927	
	(0.1801)	
poor	-0.0163	
	(0.2415)	
nearpoor	-0.0635	
	(0.3307)	
middleinc	-0.3384	
	(0.2250)	
highinc	-0.2843	
	(0.2444)	

_bs_1		7.7628e+09***
		(2.5552e+09)
_bs_2		0.3154***
		(0.0924)
_cons	-4.1216***	
	(0.4102)	
glm		
overweight	1684.0910	
	(3313.8903)	
obese	2990.0399	
	(3166.1976)	
unins	-5774.5219	
	(3603.5567)	
medicaid	345.1821	
	(4074.6451)	
medicare	10725.3856**	
	(4360.7032)	
female	-5691.2832**	
	(2586.6656)	
smoker	7140.4047**	
	(3021.4195)	
married	6888.2930**	
	(2965.8311)	
black	-563.5131	
	(4273.1068)	
amind	-5033.7443	
	(5901.5116)	
asian	-4264.2046	
	(3587.4922)	
othrace	0.0000	
	(.)	
midwest	6827.7689*	
	(3902.8600)	
south	3035.7154	
	(3745.6863)	
west	8826.7765**	
	(3956.9235)	
lesshischool	2100.3848	
	(3537.4515)	
collegeplus	4238.9668	
	(3172.2222)	
poor	1949.2996	
	(3907.1575)	
nearpoor	6290.9965	
	(5249.4572)	
middleinc	751.9616	

	(3671.3290)	
highinc	2136.2558	
	(3909.2107)	
_cons	2195.4252	
	(6424.2101)	
No. of Obs.	3885.0000	3885.0000
R-Squared		

	(1)	(2)
	inpatient	
	Coef./std.errors	Coef./std.errors
main		
overweight	0.0407	
	(0.1657)	
obese	0.1778	
	(0.1600)	
unins	-0.0659	
	(0.1942)	
medicaid	0.8443***	
	(0.2190)	
medicare	0.5240**	
	(0.2254)	
female	0.1924	
	(0.1344)	
smoker	-0.2148	
	(0.1544)	
married	-0.1382	
	(0.1454)	
black	0.3820	
	(0.2336)	
amind	-0.2063	
	(0.5460)	
asian	0.4460**	
	(0.2084)	
othrace	0.0000	
	(.)	
midwest	-0.1453	
	(0.2083)	
south	0.0168	
	(0.1860)	
west	-0.4789**	
	(0.2179)	
lesshischool	-0.1243	
	(0.1896)	
collegeplus	0.0374	
	(0.1523)	
poor	0.5590**	
	(0.2291)	
nearpoor	0.6020**	
	(0.2986)	
middleinc	-0.0838	
	(0.2252)	
highinc	-0.1426	
	(0.2374)	

_bs_1		2.4709e+09
		(3.0308e+09)
_bs_2		0.0736
		(0.0901)
_cons	-3.0469***	
	(0.3616)	
glm		
overweight	2870.3255	
	(3208.5088)	
obese	993.2829	
	(3055.4536)	
unins	-3914.4257	
	(3798.5550)	
medicaid	1795.7142	
	(3810.8131)	
medicare	-538.8949	
	(4037.0150)	
female	-841.3098	
	(2625.5151)	
smoker	-2677.4154	
	(2932.8308)	
married	-43.5838	
	(2866.9823)	
black	-1225.1217	
	(4370.3699)	
amind	-8684.1771	
	(10857.1021)	
asian	-1524.9920	
	(3828.9969)	
othrace	0.0000	
	(.)	
midwest	274.5786	
	(3965.2617)	
south	-376.4266	
	(3617.6786)	
west	7250.5575*	
	(4223.5758)	
lesshischool	-76.3569	
	(3643.8596)	
collegeplus	-824.5754	
	(2870.5802)	
poor	281.2429	
	(4325.3223)	
nearpoor	-2765.0684	
	(5631.6894)	
middleinc	1993.0306	

	(4329.2142)	
highinc	-1677.7935	
	(4386.6672)	
_cons	15074.9540**	
	(6859.9428)	
No. of Obs.	3914.0000	3914.0000
R-Squared		

	(1)	(2)
	inpatient	
	Coef./std.errors	Coef./std.errors
main		
overweight	0.1241	
	(0.1801)	
obese	0.3655**	
	(0.1735)	
unins	-0.2826	
	(0.2142)	
medicaid	0.6721***	
	(0.2227)	
medicare	0.7763***	
	(0.1870)	
female	-0.1186	
	(0.1371)	
smoker	0.0241	
	(0.1647)	
married	-0.1346	
	(0.1469)	
black	0.1245	
	(0.2486)	
amind	-0.4749	
	(0.5032)	
asian	0.1625	
	(0.2143)	
othrace	0.0000	
	(.)	
midwest	0.0028	
	(0.2192)	
south	0.1833	
	(0.1922)	
west	-0.2047	
	(0.2270)	
lesshischool	0.0865	
	(0.1840)	
collegeplus	-0.1179	
	(0.1601)	
poor	0.0902	
	(0.2531)	
nearpoor	0.4640	
	(0.3033)	
middleinc	0.3682	
	(0.2299)	
highinc	-0.1275	
	(0.2479)	

_bs_1		5.6571e+09*
		(3.3125e+09)
_bs_2		0.1411*
		(0.0810)
_cons	-2.7020***	
	(0.3806)	
glm		
overweight	2619.2003	
	(2936.2388)	
obese	1357.3099	
	(2763.5348)	
unins	-5082.3697	
	(3491.8059)	
medicaid	-3993.9362	
	(3417.4359)	
medicare	-183.8857	
	(2711.2971)	
female	-1284.7015	
	(2106.8592)	
smoker	-6051.2516**	
	(2512.3005)	
married	-479.4268	
	(2326.2148)	
black	8039.8451**	
	(3773.4046)	
amind	4117.3958	
	(8146.7286)	
asian	2552.5057	
	(3177.5700)	
othrace	0.0000	
	(.)	
midwest	3929.6259	
	(3371.0944)	
south	2836.6296	
	(2946.1071)	
west	3153.7192	
	(3667.5371)	
lesshischool	-1382.7343	
	(2818.5224)	
collegeplus	-3640.5991	
	(2495.7235)	
poor	1055.3340	
	(4019.2957)	
nearpoor	1292.4826	
	(4635.9004)	
middleinc	4057.3621	

	(3621.8738)	
highinc	7746.9803*	
	(3985.8809)	
_cons	9762.1985*	
	(5933.0252)	
No. of Obs.	2892.0000	2892.0000
R-Squared		

	(1)	(2)
	inpatient	
	Coef./std.errors	Coef./std.errors
main		
overweight	-0.0711	
	(0.1716)	
obese	0.0809	
	(0.1747)	
female	-0.0868	
	(0.1428)	
smoker	-0.2034	
	(0.2009)	
married	-0.0122	
	(0.1502)	
black	0.3667	
	(0.2534)	
amind	-0.3776	
	(0.4806)	
asian	0.1004	
	(0.2270)	
othrace	0.0000	
	(.)	
midwest	0.3876*	
	(0.2355)	
south	0.2470	
	(0.2182)	
west	0.2135	
	(0.2444)	
lesshischool	0.0290	
	(0.1834)	
collegeplus	-0.0851	
	(0.1712)	
poor	-0.0937	
	(0.2244)	
nearpoor	0.1205	
	(0.2634)	
middleinc	-0.5537**	
	(0.2171)	
highinc	-0.3190	
	(0.2135)	
_bs_1		-2.7140e+08
		(5.0935e+09)
_bs_2		-0.0066
		(0.1216)
_cons	-1.7889***	
	(0.3705)	

glm		
overweight	-4931.2107	
	(5580.9473)	
obese	-1521.8501	
	(5575.1511)	
female	924.7976	
	(4713.9338)	
smoker	4345.5213	
	(6298.9492)	
married	6614.0745	
	(5100.0005)	
black	-6264.4894	
	(7801.1400)	
amind	83799.2586***	
	(15905.1242)	
asian	-7852.0482	
	(7001.8570)	
othrace	0.0000	
	(.)	
midwest	-5552.7305	
	(7522.2236)	
south	-9512.4198	
	(7051.7990)	
west	-4814.7757	
	(8106.8257)	
lesshischool	-5091.9424	
	(5705.4504)	
collegeplus	-2801.7920	
	(5665.9909)	
poor	13108.7461*	
	(7017.8303)	
nearpoor	1972.5231	
	(8211.1285)	
middleinc	-694.3279	
	(6971.1117)	
highinc	7079.5527	
	(6866.5864)	
_cons	24387.1751**	
	(11989.2917)	
No. of Obs.	1788.0000	1788.0000
R-Squared		

	(1)	(2)
	inpatient	
	Coef./std.errors	Coef./std.errors
main		
overweight	-0.1800	
	(0.1471)	
obese	0.0851	
	(0.1744)	
female	0.1511	
	(0.1459)	
smoker	0.2375	
	(0.2430)	
married	-0.3275**	
	(0.1488)	
black	0.1684	
	(0.2729)	
amind	-0.0440	
	(0.4946)	
asian	0.4063*	
	(0.2308)	
othrace	0.0000	
	(.)	
midwest	0.0650	
	(0.2063)	
south	0.1648	
	(0.1847)	
west	-0.3175	
	(0.2239)	
lesshischool	0.2553	
	(0.1635)	
collegeplus	-0.0958	
	(0.1743)	
poor	0.1448	
	(0.2038)	
nearpoor	0.0239	
	(0.2376)	
middleinc	-0.2588	
	(0.1944)	
highinc	0.3273	
	(0.2016)	
_bs_1		-3.2617e+09
		(2.6134e+09)
_bs_2		-0.0638
		(0.0487)
_cons	-1.7877***	
	(0.3531)	

glm		
overweight	-6349.9075*	
	(3497.7324)	
obese	-6636.9903	
	(4059.4558)	
female	-4220.1490	
	(3602.1244)	
smoker	-1869.9759	
	(5691.8250)	
married	181.6556	
	(3639.2688)	
black	-8107.1800	
	(6709.0367)	
amind	-475.7672	
	(12459.2629)	
asian	-7005.8709	
	(5775.6105)	
othrace	0.0000	
	(.)	
midwest	-6929.3034	
	(4875.9886)	
south	1040.6784	
	(4363.4869)	
west	-8683.7301	
	(5644.2521)	
lesshischool	-104.5715	
	(3944.6007)	
collegeplus	-4726.3208	
	(4239.8491)	
poor	-1170.3682	
	(4829.6649)	
nearpoor	177.3089	
	(5588.1297)	
middleinc	3726.0471	
	(4670.0978)	
highinc	6770.6868	
	(4736.7131)	
_cons	30602.7438***	
	(8971.9963)	
No. of Obs.	1569.0000	1569.0000
R-Squared		

	(1)	(2)
	inpatient	
	Coef./std.errors	Coef./std.errors
main		
overweight	0.0397	
	(0.0691)	
obese	0.3142***	
	(0.0689)	
age3544	-0.4009***	
	(0.0985)	
age4554	-0.1961**	
	(0.0935)	
age5564	0.1120	
	(0.0934)	
age6574	0.4727***	
	(0.0916)	
age75plus	0.8456***	
	(0.0902)	
female	0.4513***	
	(0.0594)	
smoker	0.0332	
	(0.0750)	
married	0.0774	
	(0.0605)	
black	0.1399	
	(0.0992)	
amind	-0.0391	
	(0.1736)	
asian	0.0814	
	(0.0859)	
othrace	0.0000	
	(.)	
midwest	0.1499*	
	(0.0906)	
south	0.1026	
	(0.0833)	
west	-0.0940	
	(0.0937)	
lesshischool	0.0792	
	(0.0754)	
collegeplus	-0.0198	
	(0.0682)	
poor	0.4048***	
	(0.0925)	
nearpoor	0.1713	
	(0.1219)	

middleinc	-0.2740***	
	(0.0886)	
highinc	-0.3763***	
	(0.0923)	
_bs_1		1.5937e+10**
		(6.8003e+09)
_bs_2		0.0747**
		(0.0323)
_cons	-2.7393***	
	(0.1490)	
glm		
overweight	-1342.0693	
	(1496.3188)	
obese	-212.9111	
	(1486.0076)	
age3544	4193.2001*	
	(2205.2329)	
age4554	5325.9214**	
	(2075.1361)	
age5564	6304.3689***	
	(2082.1172)	
age6574	8513.0130***	
	(2018.8146)	
age75plus	7373.2446***	
	(1982.3560)	
female	-2739.4518**	
	(1341.6161)	
smoker	-708.7571	
	(1643.0946)	
married	695.0102	
	(1350.7043)	
black	-715.2612	
	(2156.0719)	
amind	8071.3503**	
	(3825.0258)	
asian	-1596.0002	
	(1855.0132)	
othrace	0.0000	
	(.)	
midwest	-674.3758	
	(1964.5481)	
south	-647.9895	
	(1797.8004)	
west	1369.2276	
	(2062.5835)	
lesshischool	66.2178	

	(1588.2566)	
collegeplus	-1940.8780	
	(1500.4080)	
poor	2236.8637	
	(1981.7048)	
nearpoor	2215.4622	
	(2587.5626)	
middleinc	2487.2337	
	(1932.3329)	
highinc	5338.4772***	
	(1995.3104)	
_cons	10491.4097***	
	(3340.8441)	
No. of Obs.	15175.0000	15175.0000
R-Squared		

	(1)	(2)
	inpatient	
	Coef./std.errors	Coef./std.errors
main		
overweight	0.0943	
	(0.0925)	
obese	0.3932***	
	(0.0938)	
age3544	-0.3582***	
	(0.1230)	
age4554	-0.2599**	
	(0.1204)	
age5564	-0.0006	
	(0.1219)	
age6574	0.6441***	
	(0.1349)	
age75plus	1.2545***	
	(0.1299)	
female	0.5754***	
	(0.0785)	
smoker	-0.0926	
	(0.1112)	
married	0.3526***	
	(0.0838)	
black	0.1383	
	(0.1581)	
amind	0.2996	
	(0.2143)	
asian	0.1993	
	(0.1314)	
othrace	0.0000	
	(.)	
midwest	0.1403	
	(0.1180)	
south	0.1584	
	(0.1120)	
west	-0.0178	
	(0.1262)	
lesshischool	-0.0878	
	(0.1210)	
collegeplus	-0.0194	
	(0.0856)	
poor	0.2202	
	(0.1773)	
nearpoor	0.1092	
	(0.2132)	

middleinc	-0.1390	
	(0.1222)	
highinc	-0.2610**	
	(0.1238)	
_bs_1		1.5000e+10***
		(5.1571e+09)
_bs_2		0.1056***
		(0.0368)
_cons	-3.3757***	
	(0.2147)	
glm		
overweight	-48.3398	
	(2145.4784)	
obese	657.2754	
	(2140.8243)	
age3544	2675.9633	
	(2878.5112)	
age4554	4726.5321*	
	(2813.9610)	
age5564	8963.2408***	
	(2888.3463)	
age6574	10224.2000***	
	(3131.4725)	
age75plus	5827.9200*	
	(2992.9497)	
female	-1015.0766	
	(1889.3501)	
smoker	-1418.5133	
	(2585.9541)	
married	350.0539	
	(1900.0883)	
black	4287.5829	
	(3703.1181)	
amind	15374.1700***	
	(4946.9518)	
asian	2061.9883	
	(3048.7988)	
othrace	0.0000	
	(.)	
midwest	192.0746	
	(2697.1409)	
south	-1174.1773	
	(2562.0536)	
west	2143.7050	
	(2921.5027)	
lesshischool	1500.3561	

	(2724.5845)	
collegeplus	-1959.8475	
	(1996.6042)	
poor	-887.3945	
	(4015.6845)	
nearpoor	1810.8728	
	(4785.6879)	
middleinc	1453.1394	
	(2799.4525)	
highinc	2541.7664	
	(2836.7083)	
_cons	7130.5411	
	(5009.9071)	
No. of Obs.	11061.0000	11061.0000
R-Squared		

	(1)	(2)
	inpatient	
	Coef./std.errors	Coef./std.errors
main		
overweight	0.0873	
	(0.1434)	
obese	0.1598	
	(0.1353)	
age3544	-0.5461***	
	(0.1857)	
age4554	-0.0059	
	(0.1682)	
age5564	0.1613	
	(0.1874)	
age6574	0.1376	
	(0.1900)	
age75plus	0.2430	
	(0.1912)	
female	0.2618**	
	(0.1308)	
smoker	-0.1031	
	(0.1318)	
married	-0.1051	
	(0.1326)	
black	-0.0237	
	(0.1640)	
amind	-0.4615	
	(0.4218)	
asian	0.1297	
	(0.1511)	
othrace	0.0000	
	(.)	
midwest	0.4951**	
	(0.1924)	
south	0.2635	
	(0.1754)	
west	-0.0433	
	(0.1807)	
lesshischool	0.0674	
	(0.1351)	
collegeplus	0.2442	
	(0.1663)	
poor	0.3774**	
	(0.1628)	
nearpoor	0.2626	
	(0.2085)	

middleinc	0.1884	
	(0.2094)	
highinc	-0.3124	
	(0.4367)	
_bs_1		3.8611e+09
		(2.4997e+09)
_bs_2		0.1415
		(0.0897)
_cons	-2.2210***	
	(0.2747)	
glm		
overweight	1307.7783	
	(2556.1437)	
obese	2937.5579	
	(2427.0935)	
age3544	6985.5575*	
	(3570.5747)	
age4554	8664.9291***	
	(3072.2427)	
age5564	1232.1119	
	(3452.3596)	
age6574	11450.7178***	
	(3497.5120)	
age75plus	3586.6364	
	(3400.0125)	
female	-4900.8808*	
	(2519.0764)	
smoker	854.9366	
	(2428.2256)	
married	540.5378	
	(2495.1814)	
black	-1160.3977	
	(3053.2930)	
amind	-7770.4080	
	(8055.4130)	
asian	-2799.0542	
	(2724.9466)	
othrace	0.0000	
	(.)	
midwest	-441.1397	
	(3479.7089)	
south	-3664.7780	
	(3226.2130)	
west	3632.1731	
	(3295.3814)	
lesshischool	741.4958	

	(2446.4171)	
collegeplus	-1178.5213	
	(3023.7249)	
poor	5172.6391*	
	(3020.8129)	
nearpoor	4678.2682	
	(3773.2541)	
middleinc	2261.5808	
	(3830.3614)	
highinc	6476.1714	
	(8178.0893)	
_cons	7571.9173	
	(5069.9334)	
No. of Obs.	2245.0000	2245.0000
R-Squared		

	(1)	(2)
	inpatient	
	Coef./std.errors	Coef./std.errors
main		
overweight	-0.0778	
	(0.1045)	
obese	0.1786	
	(0.1091)	
age3544	0.2162	
	(0.4525)	
age4554	0.0135	
	(0.4160)	
age5564	0.2490	
	(0.4019)	
age6574	-0.1664	
	(0.3826)	
age75plus	0.1857	
	(0.3822)	
female	0.0820	
	(0.0913)	
smoker	-0.0188	
	(0.1264)	
married	-0.1468	
	(0.0951)	
black	0.0630	
	(0.1639)	
amind	-0.5121	
	(0.3366)	
asian	0.0076	
	(0.1427)	
othrace	0.0000	
	(.)	
midwest	0.1575	
	(0.1399)	
south	0.0880	
	(0.1264)	
west	-0.1059	
	(0.1492)	
lesshischool	0.0272	
	(0.1090)	
collegeplus	-0.0994	
	(0.1085)	
poor	0.0825	
	(0.1337)	
nearpoor	0.2189	
	(0.1589)	

middleinc	-0.2334*	
	(0.1325)	
highinc	0.0741	
	(0.1375)	
_bs_1		-3.4107e+09
		(6.2167e+09)
_bs_2		-0.0313
		(0.0557)
_cons	-1.5968***	
	(0.4267)	
glm		
overweight	-5907.6720**	
	(2745.2663)	
obese	-4422.5103	
	(2889.1435)	
age3544	6918.3370	
	(11686.4263)	
age4554	-2807.1766	
	(10846.5192)	
age5564	-1647.1339	
	(10449.7767)	
age6574	-463.9785	
	(10003.8583)	
age75plus	-1450.6572	
	(10067.6837)	
female	-2126.5168	
	(2467.3036)	
smoker	157.8656	
	(3298.5305)	
married	3503.6610	
	(2632.2122)	
black	-4369.6474	
	(4297.7534)	
amind	42099.7292***	
	(9239.1008)	
asian	-4710.1593	
	(3756.2672)	
othrace	0.0000	
	(.)	
midwest	-4349.2425	
	(3643.1770)	
south	-693.5824	
	(3289.4661)	
west	-1769.0440	
	(4064.7493)	
lesshischool	-1763.4468	

	(2857.8510)	
collegeplus	-4289.6647	
	(2884.7446)	
poor	3059.2476	
	(3499.8449)	
nearpoor	1559.6471	
	(4096.6794)	
middleinc	1656.5610	
	(3517.3535)	
highinc	7712.8767**	
	(3578.8089)	
_cons	24796.7558**	
	(11601.8260)	
No. of Obs.	3861.0000	3861.0000
R-Squared		

	(1)	(2)
	inpatient	
	Coef./std.errors	Coef./std.errors
main		
overweight	0.3188**	
	(0.1274)	
obese	0.3504***	
	(0.1290)	
unins	-0.4889***	
	(0.1501)	
medicaid	0.6736***	
	(0.1591)	
medicare	0.7689*	
	(0.4022)	
female	1.7721***	
	(0.1527)	
smoker	-0.1062	
	(0.1398)	
married	0.6839***	
	(0.1102)	
black	-0.1335	
	(0.1670)	
amind	0.1889	
	(0.3108)	
asian	0.0560	
	(0.1408)	
othrace	0.0000	
	(.)	
midwest	0.1316	
	(0.1928)	
south	0.1987	
	(0.1753)	
west	-0.1588	
	(0.1868)	
lesshischool	-0.1058	
	(0.1345)	
collegeplus	-0.0341	
	(0.1338)	
poor	0.5598***	
	(0.1603)	
nearpoor	0.0143	
	(0.2299)	
middleinc	-0.1078	
	(0.1687)	
highinc	-0.3854*	
	(0.1997)	

_bs_1		3.3369e+09**
		(1.5666e+09)
_bs_2		0.0993**
		(0.0436)
_cons	-4.3375***	
	(0.2869)	
glm		
overweight	852.8192	
	(1269.4494)	
obese	877.0597	
	(1272.4061)	
unins	-3793.8168**	
	(1608.6482)	
medicaid	-1825.1314	
	(1545.4380)	
medicare	7227.2773**	
	(3663.3823)	
female	-5159.5896***	
	(1658.6007)	
smoker	70.3581	
	(1487.6374)	
married	434.1625	
	(1198.9775)	
black	3143.6703*	
	(1671.1615)	
amind	-1114.6730	
	(3208.3186)	
asian	1187.6286	
	(1461.1929)	
othrace	0.0000	
	(.)	
midwest	-1634.2903	
	(1939.3854)	
south	-1551.9261	
	(1782.3810)	
west	33.6626	
	(1869.5516)	
lesshischool	2083.3418	
	(1329.2047)	
collegeplus	-1174.9257	
	(1336.6689)	
poor	-80.9104	
	(1624.0386)	
nearpoor	5930.7125**	
	(2334.2632)	
middleinc	2261.3042	

	(1715.7756)	
highinc	4550.3952**	
	(2008.2560)	
_cons	11407.3418***	
	(2746.0026)	
No. of Obs.	5848.0000	5848.0000
R-Squared		

	(1)	(2)
	inpatient	
	Coef./std.errors	Coef./std.errors
main		
overweight	0.2002	
	(0.1989)	
obese	0.8319***	
	(0.1812)	
unins	-0.2825	
	(0.2083)	
medicaid	0.4012*	
	(0.2369)	
medicare	1.2547***	
	(0.2935)	
female	0.7683***	
	(0.1601)	
smoker	0.1365	
	(0.1778)	
married	0.0640	
	(0.1637)	
black	0.2621	
	(0.2566)	
amind	0.7167**	
	(0.3598)	
asian	0.3627*	
	(0.2166)	
othrace	0.0000	
	(.)	
midwest	0.3720	
	(0.2382)	
south	-0.0164	
	(0.2315)	
west	-0.0246	
	(0.2470)	
lesshischool	0.2243	
	(0.2159)	
collegeplus	0.1927	
	(0.1801)	
poor	-0.0163	
	(0.2415)	
nearpoor	-0.0635	
	(0.3307)	
middleinc	-0.3384	
	(0.2250)	
highinc	-0.2843	
	(0.2444)	

_bs_1		1.8845e+09
		(2.0608e+09)
_bs_2		0.0766
		(0.0842)
_cons	-4.1216***	
	(0.4102)	
glm		
overweight	1684.0910	
	(3313.8903)	
obese	2990.0399	
	(3166.1976)	
unins	-5774.5219	
	(3603.5567)	
medicaid	345.1821	
	(4074.6451)	
medicare	10725.3856**	
	(4360.7032)	
female	-5691.2832**	
	(2586.6656)	
smoker	7140.4047**	
	(3021.4195)	
married	6888.2930**	
	(2965.8311)	
black	-563.5131	
	(4273.1068)	
amind	-5033.7443	
	(5901.5116)	
asian	-4264.2046	
	(3587.4922)	
othrace	0.0000	
	(.)	
midwest	6827.7689*	
	(3902.8600)	
south	3035.7154	
	(3745.6863)	
west	8826.7765**	
	(3956.9235)	
lesshischool	2100.3848	
	(3537.4515)	
collegeplus	4238.9668	
	(3172.2222)	
poor	1949.2996	
	(3907.1575)	
nearpoor	6290.9965	
	(5249.4572)	
middleinc	751.9616	

	(3671.3290)	
highinc	2136.2558	
	(3909.2107)	
_cons	2195.4252	
	(6424.2101)	
No. of Obs.	3885.0000	3885.0000
R-Squared		

	(1)	(2)
	inpatient	
	Coef./std.errors	Coef./std.errors
main		
overweight	0.0407	
	(0.1657)	
obese	0.1778	
	(0.1600)	
unins	-0.0659	
	(0.1942)	
medicaid	0.8443***	
	(0.2190)	
medicare	0.5240**	
	(0.2254)	
female	0.1924	
	(0.1344)	
smoker	-0.2148	
	(0.1544)	
married	-0.1382	
	(0.1454)	
black	0.3820	
	(0.2336)	
amind	-0.2063	
	(0.5460)	
asian	0.4460**	
	(0.2084)	
othrace	0.0000	
	(.)	
midwest	-0.1453	
	(0.2083)	
south	0.0168	
	(0.1860)	
west	-0.4789**	
	(0.2179)	
lesshischool	-0.1243	
	(0.1896)	
collegeplus	0.0374	
	(0.1523)	
poor	0.5590**	
	(0.2291)	
nearpoor	0.6020**	
	(0.2986)	
middleinc	-0.0838	
	(0.2252)	
highinc	-0.1426	
	(0.2374)	

_bs_1		2.7462e+09
		(2.8928e+09)
_bs_2		0.0819
		(0.0857)
_cons	-3.0469***	
	(0.3616)	
glm		
overweight	2870.3255	
	(3208.5088)	
obese	993.2829	
	(3055.4536)	
unins	-3914.4257	
	(3798.5550)	
medicaid	1795.7142	
	(3810.8131)	
medicare	-538.8949	
	(4037.0150)	
female	-841.3098	
	(2625.5151)	
smoker	-2677.4154	
	(2932.8308)	
married	-43.5838	
	(2866.9823)	
black	-1225.1217	
	(4370.3699)	
amind	-8684.1771	
	(10857.1021)	
asian	-1524.9920	
	(3828.9969)	
othrace	0.0000	
	(.)	
midwest	274.5786	
	(3965.2617)	
south	-376.4266	
	(3617.6786)	
west	7250.5575*	
	(4223.5758)	
lesshischool	-76.3569	
	(3643.8596)	
collegeplus	-824.5754	
	(2870.5802)	
poor	281.2429	
	(4325.3223)	
nearpoor	-2765.0684	
	(5631.6894)	
middleinc	1993.0306	

	(4329.2142)	
highinc	-1677.7935	
	(4386.6672)	
_cons	15074.9540**	
	(6859.9428)	
No. of Obs.	3914.0000	3914.0000
R-Squared		

	(1)	(2)
	inpatient	
	Coef./std.errors	Coef./std.errors
main		
overweight	0.1241	
	(0.1801)	
obese	0.3655**	
	(0.1735)	
unins	-0.2826	
	(0.2142)	
medicaid	0.6721***	
	(0.2227)	
medicare	0.7763***	
	(0.1870)	
female	-0.1186	
	(0.1371)	
smoker	0.0241	
	(0.1647)	
married	-0.1346	
	(0.1469)	
black	0.1245	
	(0.2486)	
amind	-0.4749	
	(0.5032)	
asian	0.1625	
	(0.2143)	
othrace	0.0000	
	(.)	
midwest	0.0028	
	(0.2192)	
south	0.1833	
	(0.1922)	
west	-0.2047	
	(0.2270)	
lesshischool	0.0865	
	(0.1840)	
collegeplus	-0.1179	
	(0.1601)	
poor	0.0902	
	(0.2531)	
nearpoor	0.4640	
	(0.3033)	
middleinc	0.3682	
	(0.2299)	
highinc	-0.1275	
	(0.2479)	

_bs_1		3.5673e+09
		(3.5705e+09)
_bs_2		0.0890
		(0.0869)
_cons	-2.7020***	
	(0.3806)	
glm		
overweight	2619.2003	
	(2936.2388)	
obese	1357.3099	
	(2763.5348)	
unins	-5082.3697	
	(3491.8059)	
medicaid	-3993.9362	
	(3417.4359)	
medicare	-183.8857	
	(2711.2971)	
female	-1284.7015	
	(2106.8592)	
smoker	-6051.2516**	
	(2512.3005)	
married	-479.4268	
	(2326.2148)	
black	8039.8451**	
	(3773.4046)	
amind	4117.3958	
	(8146.7286)	
asian	2552.5057	
	(3177.5700)	
othrace	0.0000	
	(.)	
midwest	3929.6259	
	(3371.0944)	
south	2836.6296	
	(2946.1071)	
west	3153.7192	
	(3667.5371)	
lesshischool	-1382.7343	
	(2818.5224)	
collegeplus	-3640.5991	
	(2495.7235)	
poor	1055.3340	
	(4019.2957)	
nearpoor	1292.4826	
	(4635.9004)	
middleinc	4057.3621	

	(3621.8738)	
highinc	7746.9803*	
	(3985.8809)	
_cons	9762.1985*	
	(5933.0252)	
No. of Obs.	2892.0000	2892.0000
R-Squared		

	(1)	(2)
	inpatient	
	Coef./std.errors	Coef./std.errors
main		
overweight	-0.0711	
	(0.1716)	
obese	0.0809	
	(0.1747)	
female	-0.0868	
	(0.1428)	
smoker	-0.2034	
	(0.2009)	
married	-0.0122	
	(0.1502)	
black	0.3667	
	(0.2534)	
amind	-0.3776	
	(0.4806)	
asian	0.1004	
	(0.2270)	
othrace	0.0000	
	(.)	
midwest	0.3876*	
	(0.2355)	
south	0.2470	
	(0.2182)	
west	0.2135	
	(0.2444)	
lesshischool	0.0290	
	(0.1834)	
collegeplus	-0.0851	
	(0.1712)	
poor	-0.0937	
	(0.2244)	
nearpoor	0.1205	
	(0.2634)	
middleinc	-0.5537**	
	(0.2171)	
highinc	-0.3190	
	(0.2135)	
_bs_1		-5.1938e+09
		(4.9133e+09)
_bs_2		-0.1259
		(0.1107)
_cons	-1.7889***	
	(0.3705)	

glm		
overweight	-4931.2107	
	(5580.9473)	
obese	-1521.8501	
	(5575.1511)	
female	924.7976	
	(4713.9338)	
smoker	4345.5213	
	(6298.9492)	
married	6614.0745	
	(5100.0005)	
black	-6264.4894	
	(7801.1400)	
amind	83799.2586***	
	(15905.1242)	
asian	-7852.0482	
	(7001.8570)	
othrace	0.0000	
	(.)	
midwest	-5552.7305	
	(7522.2236)	
south	-9512.4198	
	(7051.7990)	
west	-4814.7757	
	(8106.8257)	
lesshischool	-5091.9424	
	(5705.4504)	
collegeplus	-2801.7920	
	(5665.9909)	
poor	13108.7461*	
	(7017.8303)	
nearpoor	1972.5231	
	(8211.1285)	
middleinc	-694.3279	
	(6971.1117)	
highinc	7079.5527	
	(6866.5864)	
_cons	24387.1751**	
	(11989.2917)	
No. of Obs.	1788.0000	1788.0000
R-Squared		

	(1)	(2)
	inpatient	
	Coef./std.errors	Coef./std.errors
main		
overweight	-0.1800	
	(0.1471)	
obese	0.0851	
	(0.1744)	
female	0.1511	
	(0.1459)	
smoker	0.2375	
	(0.2430)	
married	-0.3275**	
	(0.1488)	
black	0.1684	
	(0.2729)	
amind	-0.0440	
	(0.4946)	
asian	0.4063*	
	(0.2308)	
othrace	0.0000	
	(.)	
midwest	0.0650	
	(0.2063)	
south	0.1648	
	(0.1847)	
west	-0.3175	
	(0.2239)	
lesshischool	0.2553	
	(0.1635)	
collegeplus	-0.0958	
	(0.1743)	
poor	0.1448	
	(0.2038)	
nearpoor	0.0239	
	(0.2376)	
middleinc	-0.2588	
	(0.1944)	
highinc	0.3273	
	(0.2016)	
_bs_1		-9.5995e+09*
		(5.5935e+09)
_bs_2		-0.1879*
		(0.1032)
_cons	-1.7877***	
	(0.3531)	

glm		
overweight	-6349.9075*	
	(3497.7324)	
obese	-6636.9903	
	(4059.4558)	
female	-4220.1490	
	(3602.1244)	
smoker	-1869.9759	
	(5691.8250)	
married	181.6556	
	(3639.2688)	
black	-8107.1800	
	(6709.0367)	
amind	-475.7672	
	(12459.2629)	
asian	-7005.8709	
	(5775.6105)	
othrace	0.0000	
	(.)	
midwest	-6929.3034	
	(4875.9886)	
south	1040.6784	
	(4363.4869)	
west	-8683.7301	
	(5644.2521)	
lesshischool	-104.5715	
	(3944.6007)	
collegeplus	-4726.3208	
	(4239.8491)	
poor	-1170.3682	
	(4829.6649)	
nearpoor	177.3089	
	(5588.1297)	
middleinc	3726.0471	
	(4670.0978)	
highinc	6770.6868	
	(4736.7131)	
_cons	30602.7438***	
	(8971.9963)	
No. of Obs.	1569.0000	1569.0000
R-Squared		

	(1)	(2)
	inpatient	
	Coef./std.errors	Coef./std.errors
main		
overweight	0.0397	
	(0.0691)	
obese	0.3142***	
	(0.0689)	
age3544	-0.4009***	
	(0.0985)	
age4554	-0.1961**	
	(0.0935)	
age5564	0.1120	
	(0.0934)	
age6574	0.4727***	
	(0.0916)	
age75plus	0.8456***	
	(0.0902)	
female	0.4513***	
	(0.0594)	
smoker	0.0332	
	(0.0750)	
married	0.0774	
	(0.0605)	
black	0.1399	
	(0.0992)	
amind	-0.0391	
	(0.1736)	
asian	0.0814	
	(0.0859)	
othrace	0.0000	
	(.)	
midwest	0.1499*	
	(0.0906)	
south	0.1026	
	(0.0833)	
west	-0.0940	
	(0.0937)	
lesshischool	0.0792	
	(0.0754)	
collegeplus	-0.0198	
	(0.0682)	
poor	0.4048***	
	(0.0925)	
nearpoor	0.1713	
	(0.1219)	

middleinc	-0.2740***	
	(0.0886)	
highinc	-0.3763***	
	(0.0923)	
_bs_1		-3.7452e+09
		(8.5965e+09)
_bs_2		-0.0175
		(0.0400)
_cons	-2.7393***	
	(0.1490)	
glm		
overweight	-1342.0693	
	(1496.3188)	
obese	-212.9111	
	(1486.0076)	
age3544	4193.2001*	
	(2205.2329)	
age4554	5325.9214**	
	(2075.1361)	
age5564	6304.3689***	
	(2082.1172)	
age6574	8513.0130***	
	(2018.8146)	
age75plus	7373.2446***	
	(1982.3560)	
female	-2739.4518**	
	(1341.6161)	
smoker	-708.7571	
	(1643.0946)	
married	695.0102	
	(1350.7043)	
black	-715.2612	
	(2156.0719)	
amind	8071.3503**	
	(3825.0258)	
asian	-1596.0002	
	(1855.0132)	
othrace	0.0000	
	(.)	
midwest	-674.3758	
	(1964.5481)	
south	-647.9895	
	(1797.8004)	
west	1369.2276	
	(2062.5835)	
lesshischool	66.2178	

	(1588.2566)	
collegeplus	-1940.8780	
	(1500.4080)	
poor	2236.8637	
	(1981.7048)	
nearpoor	2215.4622	
	(2587.5626)	
middleinc	2487.2337	
	(1932.3329)	
highinc	5338.4772***	
	(1995.3104)	
_cons	10491.4097***	
	(3340.8441)	
No. of Obs.	15175.0000	15175.0000
R-Squared		

	(1)	(2)
	inpatient	
	Coef./std.errors	Coef./std.errors
main		
overweight	0.0943	
	(0.0925)	
obese	0.3932***	
	(0.0938)	
age3544	-0.3582***	
	(0.1230)	
age4554	-0.2599**	
	(0.1204)	
age5564	-0.0006	
	(0.1219)	
age6574	0.6441***	
	(0.1349)	
age75plus	1.2545***	
	(0.1299)	
female	0.5754***	
	(0.0785)	
smoker	-0.0926	
	(0.1112)	
married	0.3526***	
	(0.0838)	
black	0.1383	
	(0.1581)	
amind	0.2996	
	(0.2143)	
asian	0.1993	
	(0.1314)	
othrace	0.0000	
	(.)	
midwest	0.1403	
	(0.1180)	
south	0.1584	
	(0.1120)	
west	-0.0178	
	(0.1262)	
lesshischool	-0.0878	
	(0.1210)	
collegeplus	-0.0194	
	(0.0856)	
poor	0.2202	
	(0.1773)	
nearpoor	0.1092	
	(0.2132)	

middleinc	-0.1390	
	(0.1222)	
highinc	-0.2610**	
	(0.1238)	
_bs_1		3.7000e+09
		(7.7183e+09)
_bs_2		0.0261
		(0.0538)
_cons	-3.3757***	
	(0.2147)	
glm		
overweight	-48.3398	
	(2145.4784)	
obese	657.2754	
	(2140.8243)	
age3544	2675.9633	
	(2878.5112)	
age4554	4726.5321*	
	(2813.9610)	
age5564	8963.2408***	
	(2888.3463)	
age6574	10224.2000***	
	(3131.4725)	
age75plus	5827.9200*	
	(2992.9497)	
female	-1015.0766	
	(1889.3501)	
smoker	-1418.5133	
	(2585.9541)	
married	350.0539	
	(1900.0883)	
black	4287.5829	
	(3703.1181)	
amind	15374.1700***	
	(4946.9518)	
asian	2061.9883	
	(3048.7988)	
othrace	0.0000	
	(.)	
midwest	192.0746	
	(2697.1409)	
south	-1174.1773	
	(2562.0536)	
west	2143.7050	
	(2921.5027)	
lesshischool	1500.3561	

	(2724.5845)	
collegeplus	-1959.8475	
	(1996.6042)	
poor	-887.3945	
	(4015.6845)	
nearpoor	1810.8728	
	(4785.6879)	
middleinc	1453.1394	
	(2799.4525)	
highinc	2541.7664	
	(2836.7083)	
_cons	7130.5411	
	(5009.9071)	
No. of Obs.	11061.0000	11061.0000
R-Squared		

	(1)	(2)
	inpatient	
	Coef./std.errors	Coef./std.errors
main		
overweight	0.0873	
	(0.1434)	
obese	0.1598	
	(0.1353)	
age3544	-0.5461***	
	(0.1857)	
age4554	-0.0059	
	(0.1682)	
age5564	0.1613	
	(0.1874)	
age6574	0.1376	
	(0.1900)	
age75plus	0.2430	
	(0.1912)	
female	0.2618**	
	(0.1308)	
smoker	-0.1031	
	(0.1318)	
married	-0.1051	
	(0.1326)	
black	-0.0237	
	(0.1640)	
amind	-0.4615	
	(0.4218)	
asian	0.1297	
	(0.1511)	
othrace	0.0000	
	(.)	
midwest	0.4951**	
	(0.1924)	
south	0.2635	
	(0.1754)	
west	-0.0433	
	(0.1807)	
lesshischool	0.0674	
	(0.1351)	
collegeplus	0.2442	
	(0.1663)	
poor	0.3774**	
	(0.1628)	
nearpoor	0.2626	
	(0.2085)	

middleinc	0.1884	
	(0.2094)	
highinc	-0.3124	
	(0.4367)	
_bs_1		1.3753e+09
		(1.6008e+09)
_bs_2		0.0504
		(0.0586)
_cons	-2.2210***	
	(0.2747)	
glm		
overweight	1307.7783	
	(2556.1437)	
obese	2937.5579	
	(2427.0935)	
age3544	6985.5575*	
	(3570.5747)	
age4554	8664.9291***	
	(3072.2427)	
age5564	1232.1119	
	(3452.3596)	
age6574	11450.7178***	
	(3497.5120)	
age75plus	3586.6364	
	(3400.0125)	
female	-4900.8808*	
	(2519.0764)	
smoker	854.9366	
	(2428.2256)	
married	540.5378	
	(2495.1814)	
black	-1160.3977	
	(3053.2930)	
amind	-7770.4080	
	(8055.4130)	
asian	-2799.0542	
	(2724.9466)	
othrace	0.0000	
	(.)	
midwest	-441.1397	
	(3479.7089)	
south	-3664.7780	
	(3226.2130)	
west	3632.1731	
	(3295.3814)	
lesshischool	741.4958	

	(2446.4171)	
collegeplus	-1178.5213	
	(3023.7249)	
poor	5172.6391*	
	(3020.8129)	
nearpoor	4678.2682	
	(3773.2541)	
middleinc	2261.5808	
	(3830.3614)	
highinc	6476.1714	
	(8178.0893)	
_cons	7571.9173	
	(5069.9334)	
No. of Obs.	2245.0000	2245.0000
R-Squared		

	(1)	(2)
	inpatient	
	Coef./std.errors	Coef./std.errors
main		
overweight	-0.0778	
	(0.1045)	
obese	0.1786	
	(0.1091)	
age3544	0.2162	
	(0.4525)	
age4554	0.0135	
	(0.4160)	
age5564	0.2490	
	(0.4019)	
age6574	-0.1664	
	(0.3826)	
age75plus	0.1857	
	(0.3822)	
female	0.0820	
	(0.0913)	
smoker	-0.0188	
	(0.1264)	
married	-0.1468	
	(0.0951)	
black	0.0630	
	(0.1639)	
amind	-0.5121	
	(0.3366)	
asian	0.0076	
	(0.1427)	
othrace	0.0000	
	(.)	
midwest	0.1575	
	(0.1399)	
south	0.0880	
	(0.1264)	
west	-0.1059	
	(0.1492)	
lesshischool	0.0272	
	(0.1090)	
collegeplus	-0.0994	
	(0.1085)	
poor	0.0825	
	(0.1337)	
nearpoor	0.2189	
	(0.1589)	

middleinc	-0.2334*	
	(0.1325)	
highinc	0.0741	
	(0.1375)	
_bs_1		-1.5040e+10**
		(6.4348e+09)
_bs_2		-0.1378**
		(0.0560)
_cons	-1.5968***	
	(0.4267)	
glm		
overweight	-5907.6720**	
	(2745.2663)	
obese	-4422.5103	
	(2889.1435)	
age3544	6918.3370	
	(11686.4263)	
age4554	-2807.1766	
	(10846.5192)	
age5564	-1647.1339	
	(10449.7767)	
age6574	-463.9785	
	(10003.8583)	
age75plus	-1450.6572	
	(10067.6837)	
female	-2126.5168	
	(2467.3036)	
smoker	157.8656	
	(3298.5305)	
married	3503.6610	
	(2632.2122)	
black	-4369.6474	
	(4297.7534)	
amind	42099.7292***	
	(9239.1008)	
asian	-4710.1593	
	(3756.2672)	
othrace	0.0000	
	(.)	
midwest	-4349.2425	
	(3643.1770)	
south	-693.5824	
	(3289.4661)	
west	-1769.0440	
	(4064.7493)	
lesshischool	-1763.4468	

	(2857.8510)	
collegeplus	-4289.6647	
	(2884.7446)	
poor	3059.2476	
	(3499.8449)	
nearpoor	1559.6471	
	(4096.6794)	
middleinc	1656.5610	
	(3517.3535)	
highinc	7712.8767**	
	(3578.8089)	
_cons	24796.7558**	
	(11601.8260)	
No. of Obs.	3861.0000	3861.0000
R-Squared		

	(1)	(2)
	totalexp	
	Coef./std.errors	Coef./std.errors
main		
overweight	0.0204	
	(0.1061)	
obese	0.1951	
	(0.1228)	
unins	-0.8434***	
	(0.1164)	
medicaid	0.4269**	
	(0.1913)	
medicare	0.6232	
	(0.5753)	
female	1.0096***	
	(0.0973)	
smoker	0.1437	
	(0.1199)	
married	0.2498**	
	(0.1027)	
black	0.0538	
	(0.1722)	
amind	-0.2361	
	(0.2478)	
asian	0.4107***	
	(0.1325)	
othrace	0.0000	
	(.)	
midwest	-0.0612	
	(0.1470)	
south	-0.1024	
	(0.1365)	
west	-0.2088	
	(0.1502)	
lesshischool	-0.0119	
	(0.1351)	
collegeplus	-0.0885	
	(0.1164)	
poor	0.2753	
	(0.1754)	
nearpoor	0.4046	
	(0.2505)	
middleinc	-0.0789	
	(0.1462)	
highinc	0.0699	
	(0.1523)	

_bs_1		4.2745e+09*
		(2.3400e+09)
_bs_2		0.0452*
		(0.0242)
_cons	6.4661***	
	(0.2287)	
No. of Obs.	5848.0000	5848.0000
R-Squared		

	(1)	(2)
	totalexp	
	Coef./std.errors	Coef./std.errors
main		
overweight	-0.0652	
	(0.1092)	
obese	0.4905***	
	(0.1150)	
unins	-0.9031***	
	(0.1195)	
medicaid	0.6286***	
	(0.2090)	
medicare	1.0692***	
	(0.3111)	
female	0.3313***	
	(0.0901)	
smoker	0.2213*	
	(0.1173)	
married	0.0062	
	(0.0974)	
black	0.4106**	
	(0.1782)	
amind	0.2468	
	(0.2258)	
asian	0.5006***	
	(0.1335)	
othrace	0.0000	
	(.)	
midwest	0.1265	
	(0.1407)	
south	-0.1090	
	(0.1285)	
west	0.0233	
	(0.1370)	
lesshischool	0.1363	
	(0.1493)	
collegeplus	0.3816***	
	(0.1085)	
poor	-0.2008	
	(0.1948)	
nearpoor	0.2493	
	(0.2660)	
middleinc	-0.2557*	
	(0.1509)	
highinc	-0.1699	
	(0.1541)	

_bs_1		1.3683e+10***
		(3.1355e+09)
_bs_2		0.1658***
		(0.0379)
_cons	6.7818***	
	(0.2380)	
No. of Obs.	3885.0000	3885.0000
R-Squared		

	(1)	(2)
	totalexp	
	Coef./std.errors	Coef./std.errors
main		
overweight	0.2736***	
	(0.0978)	
obese	0.4029***	
	(0.1004)	
unins	-0.4409***	
	(0.1143)	
medicaid	0.8227***	
	(0.2011)	
medicare	1.2996***	
	(0.2083)	
female	0.3170***	
	(0.0802)	
smoker	-0.1883**	
	(0.0951)	
married	0.0048	
	(0.0897)	
black	0.4908***	
	(0.1718)	
amind	-0.3147	
	(0.2454)	
asian	0.5055***	
	(0.1334)	
othrace	0.0000	
	(.)	
midwest	-0.0125	
	(0.1239)	
south	-0.1594	
	(0.1157)	
west	-0.1043	
	(0.1235)	
lesshischool	0.0488	
	(0.1324)	
collegeplus	0.1263	
	(0.0887)	
poor	0.3230*	
	(0.1910)	
nearpoor	-0.1076	
	(0.2660)	
middleinc	-0.0738	
	(0.1506)	
highinc	-0.0281	
	(0.1494)	

_bs_1		1.6904e+10***
		(4.6202e+09)
_bs_2		0.1297***
		(0.0318)
_cons	7.1876***	
	(0.2230)	
No. of Obs.	3914.0000	3914.0000
R-Squared		

	(1)	(2)
	totalexp	
	Coef./std.errors	Coef./std.errors
main		
overweight	0.0466	
	(0.1011)	
obese	0.4555***	
	(0.1048)	
unins	-0.4590***	
	(0.1314)	
medicaid	0.6807***	
	(0.2220)	
medicare	0.8395***	
	(0.1676)	
female	0.0140	
	(0.0847)	
smoker	-0.0650	
	(0.1079)	
married	-0.0331	
	(0.0932)	
black	0.5263***	
	(0.1977)	
amind	0.0307	
	(0.2448)	
asian	0.5825***	
	(0.1589)	
othrace	0.0000	
	(.)	
midwest	0.2383*	
	(0.1273)	
south	0.2901**	
	(0.1155)	
west	0.1142	
	(0.1316)	
lesshischool	-0.0198	
	(0.1328)	
collegeplus	-0.1009	
	(0.0973)	
poor	0.2319	
	(0.2017)	
nearpoor	0.4341	
	(0.2767)	
middleinc	0.4838***	
	(0.1634)	
highinc	0.3803**	
	(0.1643)	

_bs_1		2.4986e+10***
		(7.1668e+09)
_bs_2		0.1670***
		(0.0467)
_cons	7.2520***	
	(0.2502)	
No. of Obs.	2892.0000	2892.0000
R-Squared		

	(1)	(2)
	totalexp	
	Coef./std.errors	Coef./std.errors
main		
overweight	-0.0473	
	(0.1254)	
obese	0.2849**	
	(0.1307)	
female	-0.0561	
	(0.1069)	
smoker	-0.0497	
	(0.1487)	
married	0.1208	
	(0.1126)	
black	-0.0729	
	(0.2560)	
amind	0.2613	
	(0.3338)	
asian	-0.0936	
	(0.2021)	
othrace	0.0000	
	(.)	
midwest	0.1737	
	(0.1571)	
south	0.1125	
	(0.1391)	
west	0.1414	
	(0.1663)	
lesshischool	-0.0983	
	(0.1434)	
collegeplus	0.0649	
	(0.1220)	
poor	0.3367	
	(0.2113)	
nearpoor	0.4192*	
	(0.2313)	
middleinc	0.0154	
	(0.1632)	
highinc	-0.0253	
	(0.1647)	
_bs_1		9.9319e+09**
		(4.4815e+09)
_bs_2		0.0913**
		(0.0410)
_cons	8.5477***	
	(0.2932)	

No. of Obs.	1788.0000	1788.0000
R-Squared		

	(1)	(2)
	totalexp	
	Coef./std.errors	Coef./std.errors
main		
overweight	-0.0792	
	(0.0941)	
obese	0.1280	
	(0.1198)	
female	0.0238	
	(0.0951)	
smoker	0.0647	
	(0.1860)	
married	-0.0816	
	(0.0934)	
black	0.3679	
	(0.2431)	
amind	0.2020	
	(0.3113)	
asian	0.2700	
	(0.1911)	
othrace	0.0000	
	(.)	
midwest	-0.1310	
	(0.1262)	
south	0.1188	
	(0.1177)	
west	-0.4036***	
	(0.1381)	
lesshischool	0.0919	
	(0.1141)	
collegeplus	-0.0797	
	(0.1030)	
poor	-0.0712	
	(0.1664)	
nearpoor	0.1591	
	(0.1750)	
middleinc	-0.0038	
	(0.1203)	
highinc	0.1991	
	(0.1266)	
_bs_1		2.9394e+09
		(2.7348e+09)
_bs_2		0.0245
		(0.0226)
_cons	8.6934***	
	(0.2599)	

No. of Obs.	1569.0000	1569.0000
R-Squared		

	(1)	(2)
	totalexp	
	Coef./std.errors	Coef./std.errors
main		
overweight	0.0437	
	(0.0515)	
obese	0.3912***	
	(0.0555)	
age3544	0.2313***	
	(0.0661)	
age4554	0.6339***	
	(0.0664)	
age5564	1.0313***	
	(0.0708)	
age6574	1.2251***	
	(0.0801)	
age75plus	1.4062***	
	(0.0825)	
female	0.3588***	
	(0.0444)	
smoker	-0.0141	
	(0.0599)	
married	0.0980**	
	(0.0464)	
black	0.1814*	
	(0.0990)	
amind	-0.0161	
	(0.1264)	
asian	0.2357***	
	(0.0776)	
othrace	0.0000	
	(.)	
midwest	0.0947	
	(0.0658)	
south	0.0122	
	(0.0609)	
west	-0.0515	
	(0.0676)	
lesshischool	0.1138	
	(0.0697)	
collegeplus	0.0402	
	(0.0513)	
poor	0.3350***	
	(0.1019)	
nearpoor	0.3041**	
	(0.1315)	

middleinc	-0.1079	
	(0.0769)	
highinc	-0.1510**	
	(0.0762)	
_bs_1		7.3640e+10***
		(1.1371e+10)
_bs_2		0.1142***
		(0.0168)
_cons	6.9718***	
	(0.1230)	
No. of Obs.	15175.0000	15175.0000
R-Squared		

	(1)	(2)
	totalexp	
	Coef./std.errors	Coef./std.errors
main		
overweight	0.0715	
	(0.0635)	
obese	0.4105***	
	(0.0693)	
age3544	0.2066***	
	(0.0781)	
age4554	0.5940***	
	(0.0790)	
age5564	1.0117***	
	(0.0843)	
age6574	1.3429***	
	(0.1133)	
age75plus	1.5228***	
	(0.1210)	
female	0.4655***	
	(0.0548)	
smoker	-0.1295*	
	(0.0739)	
married	0.1774***	
	(0.0565)	
black	0.1548	
	(0.1292)	
amind	0.1423	
	(0.1568)	
asian	0.3374***	
	(0.0991)	
othrace	0.0000	
	(.)	
midwest	0.1083	
	(0.0801)	
south	-0.0122	
	(0.0753)	
west	-0.0616	
	(0.0835)	
lesshischool	0.0662	
	(0.0960)	
collegeplus	0.0395	
	(0.0629)	
poor	0.0677	
	(0.1683)	
nearpoor	0.1552	
	(0.2074)	

middleinc	-0.0577	
	(0.1022)	
highinc	-0.0999	
	(0.1004)	
_bs_1		5.2642e+10***
		(9.8169e+09)
_bs_2		0.1151***
		(0.0208)
_cons	6.7254***	
	(0.1568)	
No. of Obs.	11061.0000	11061.0000
R-Squared		

	(1)	(2)
	totalexp	
	Coef./std.errors	Coef./std.errors
main		
overweight	-0.0094	
	(0.1098)	
obese	0.3012***	
	(0.1064)	
age3544	0.4635***	
	(0.1293)	
age4554	1.0192***	
	(0.1355)	
age5564	1.0542***	
	(0.1555)	
age6574	1.0825***	
	(0.1596)	
age75plus	0.9112***	
	(0.1694)	
female	-0.0296	
	(0.0994)	
smoker	0.1052	
	(0.1034)	
married	-0.1654	
	(0.1076)	
black	0.2258	
	(0.1466)	
amind	-0.8830***	
	(0.2382)	
asian	0.1188	
	(0.1253)	
othrace	0.0000	
	(.)	
midwest	0.2749**	
	(0.1373)	
south	-0.0420	
	(0.1253)	
west	0.1612	
	(0.1264)	
lesshischool	0.0023	
	(0.1036)	
collegeplus	0.0635	
	(0.1217)	
poor	0.1436	
	(0.1255)	
nearpoor	0.3095*	
	(0.1620)	

middleinc	0.1170	
	(0.1466)	
highinc	0.1277	
	(0.2411)	
_bs_1		9.2384e+09**
		(4.1093e+09)
_bs_2		0.1147**
		(0.0497)
_cons	7.6531***	
	(0.2081)	
No. of Obs.	2245.0000	2245.0000
R-Squared		

	(1)	(2)
	totalexp	
	Coef./std.errors	Coef./std.errors
main		
overweight	-0.0657	
	(0.0770)	
obese	0.2678***	
	(0.0860)	
age3544	0.7813**	
	(0.3779)	
age4554	1.0993***	
	(0.3495)	
age5564	1.0644***	
	(0.3381)	
age6574	0.5049	
	(0.3162)	
age75plus	0.7377**	
	(0.3184)	
female	-0.0300	
	(0.0690)	
smoker	-0.0258	
	(0.1034)	
married	0.0504	
	(0.0722)	
black	0.0461	
	(0.1697)	
amind	0.1830	
	(0.2333)	
asian	-0.0180	
	(0.1376)	
othrace	0.0000	
	(.)	
midwest	0.0293	
	(0.0993)	
south	0.1050	
	(0.0899)	
west	-0.0513	
	(0.1080)	
lesshischool	0.0082	
	(0.0875)	
collegeplus	-0.0242	
	(0.0777)	
poor	0.0955	
	(0.1218)	
nearpoor	0.3156**	
	(0.1392)	

middleinc	0.0080	
	(0.0970)	
highinc	0.1428	
	(0.1001)	
_bs_1		2.2698e+10***
		(6.9622e+09)
_bs_2		0.0814***
		(0.0244)
_cons	8.0851***	
	(0.3548)	
No. of Obs.	3861.0000	3861.0000
R-Squared		

	(1)	(2)
	outpatient	
	Coef./std.errors	Coef./std.errors
main		
overweight	-0.1027	
	(0.1214)	
obese	0.3668***	
	(0.1273)	
unins	-0.9986***	
	(0.1335)	
medicaid	0.4431*	
	(0.2318)	
medicare	0.6793**	
	(0.3429)	
female	0.4223***	
	(0.1016)	
smoker	0.1162	
	(0.1289)	
married	0.0224	
	(0.1064)	
black	0.2923	
	(0.1969)	
amind	-0.2888	
	(0.2394)	
asian	0.5741***	
	(0.1449)	
othrace	0.0000	
	(.)	
midwest	0.1421	
	(0.1536)	
south	-0.1137	
	(0.1412)	
west	0.0294	
	(0.1512)	
lesshischool	0.1792	
	(0.1666)	
collegeplus	0.3630***	
	(0.1177)	
poor	-0.4114*	
	(0.2172)	
nearpoor	-0.0688	
	(0.2989)	
middleinc	-0.2957*	
	(0.1713)	
highinc	-0.3230*	
	(0.1732)	

_bs_1		4.0495e+09***
		(1.5384e+09)
_bs_2		0.1208***
		(0.0460)
_cons	6.0538***	
	(0.2667)	
No. of Obs.	3885.0000	3885.0000
R-Squared		

	(1)	(2)
	outpatient	
	Coef./std.errors	Coef./std.errors
main		
overweight	0.2410**	
	(0.0992)	
obese	0.3459***	
	(0.1026)	
unins	-0.6880***	
	(0.1130)	
medicaid	0.4028*	
	(0.2118)	
medicare	0.7299***	
	(0.2166)	
female	0.4919***	
	(0.0832)	
smoker	-0.1525	
	(0.0970)	
married	-0.1352	
	(0.0909)	
black	0.2754	
	(0.1734)	
amind	-0.2429	
	(0.2489)	
asian	0.2545*	
	(0.1365)	
othrace	0.0000	
	(.)	
midwest	0.0364	
	(0.1269)	
south	-0.3025***	
	(0.1172)	
west	-0.2172*	
	(0.1273)	
lesshischool	0.0899	
	(0.1358)	
collegeplus	0.3007***	
	(0.0895)	
poor	0.1157	
	(0.1960)	
nearpoor	-0.2806	
	(0.2738)	
middleinc	0.0267	
	(0.1528)	
highinc	0.1562	
	(0.1518)	

_bs_1		4.6757e+09***
		(1.3864e+09)
_bs_2		0.1033***
		(0.0294)
_cons	6.3855***	
	(0.2253)	
No. of Obs.	3914.0000	3914.0000
R-Squared		

	(1)	(2)
	outpatient	
	Coef./std.errors	Coef./std.errors
main		
overweight	-0.2289**	
	(0.1112)	
obese	0.1651	
	(0.1167)	
unins	-0.5553***	
	(0.1455)	
medicaid	0.7424***	
	(0.2525)	
medicare	0.9900***	
	(0.1889)	
female	0.1156	
	(0.0942)	
smoker	-0.2064*	
	(0.1203)	
married	-0.0448	
	(0.1048)	
black	0.4019*	
	(0.2274)	
amind	0.0493	
	(0.2709)	
asian	0.7409***	
	(0.1774)	
othrace	0.0000	
	(.)	
midwest	0.2339*	
	(0.1390)	
south	0.2190*	
	(0.1285)	
west	0.0263	
	(0.1451)	
lesshischool	-0.1697	
	(0.1516)	
collegeplus	-0.0301	
	(0.1077)	
poor	-0.2492	
	(0.2258)	
nearpoor	-0.3373	
	(0.3019)	
middleinc	0.2169	
	(0.1818)	
highinc	0.2714	
	(0.1815)	

_bs_1		3.6244e+09
		(3.0685e+09)
_bs_2		0.0640
		(0.0544)
_cons	6.5496***	
	(0.2874)	
No. of Obs.	2892.0000	2892.0000
R-Squared		

	(1)	(2)
	outpatient	
	Coef./std.errors	Coef./std.errors
main		
overweight	-0.0755	
	(0.1208)	
obese	0.0432	
	(0.1272)	
female	-0.1212	
	(0.1025)	
smoker	-0.3685**	
	(0.1462)	
married	0.0991	
	(0.1068)	
black	-0.1306	
	(0.2609)	
amind	-0.8884***	
	(0.3162)	
asian	-0.2287	
	(0.2061)	
othrace	0.0000	
	(.)	
midwest	0.3851**	
	(0.1543)	
south	0.1601	
	(0.1359)	
west	0.1862	
	(0.1602)	
lesshischool	-0.1978	
	(0.1423)	
collegeplus	0.3143***	
	(0.1178)	
poor	0.2264	
	(0.2059)	
nearpoor	0.3622	
	(0.2287)	
middleinc	0.2927*	
	(0.1610)	
highinc	0.1678	
	(0.1587)	
_bs_1		4.7635e+08
		(1.5809e+09)
_bs_2		0.0138
		(0.0459)
_cons	7.4022***	
	(0.2872)	

No. of Obs.	1788.0000	1788.0000
R-Squared		

	(1)	(2)
	outpatient	
	Coef./std.errors	Coef./std.errors
main		
overweight	0.0811	
	(0.0987)	
obese	0.4183***	
	(0.1234)	
female	0.0520	
	(0.0956)	
smoker	-0.1391	
	(0.1883)	
married	0.1372	
	(0.0962)	
black	0.8850***	
	(0.2509)	
amind	0.1554	
	(0.3212)	
asian	0.4540**	
	(0.1995)	
othrace	0.0000	
	(.)	
midwest	0.0897	
	(0.1296)	
south	-0.1830	
	(0.1196)	
west	-0.3427**	
	(0.1396)	
lesshischool	-0.0646	
	(0.1145)	
collegeplus	0.2552**	
	(0.1054)	
poor	-0.0445	
	(0.1729)	
nearpoor	0.1577	
	(0.1831)	
middleinc	0.1259	
	(0.1222)	
highinc	0.3251**	
	(0.1272)	
_bs_1		2.7863e+09***
		(1.0232e+09)
_bs_2		0.0822***
		(0.0282)
_cons	6.8784***	
	(0.2669)	

No. of Obs.	1569.0000	1569.0000
R-Squared		

	(1)	(2)
	outpatient	
	Coef./std.errors	Coef./std.errors
main		
overweight	0.0138	
	(0.0514)	
obese	0.3065***	
	(0.0552)	
age3544	0.2910***	
	(0.0654)	
age4554	0.5794***	
	(0.0655)	
age5564	1.0383***	
	(0.0698)	
age6574	1.0941***	
	(0.0796)	
age75plus	1.1276***	
	(0.0819)	
female	0.4013***	
	(0.0440)	
smoker	-0.0517	
	(0.0592)	
married	0.0963**	
	(0.0460)	
black	0.1853*	
	(0.0986)	
amind	-0.3514***	
	(0.1254)	
asian	0.2157***	
	(0.0778)	
othrace	0.0000	
	(.)	
midwest	0.1591**	
	(0.0656)	
south	-0.1127*	
	(0.0605)	
west	-0.0402	
	(0.0674)	
lesshischool	0.0459	
	(0.0698)	
collegeplus	0.1872***	
	(0.0505)	
poor	0.1253	
	(0.1019)	
nearpoor	0.0567	
	(0.1316)	

middleinc	-0.0501	
	(0.0770)	
highinc	-0.0897	
	(0.0762)	
_bs_1		2.0014e+10***
		(3.9859e+09)
_bs_2		0.0893***
		(0.0175)
_cons	5.9813***	
	(0.1255)	
No. of Obs.	15175.0000	15175.0000
R-Squared		

	(1)	(2)
	outpatient	
	Coef./std.errors	Coef./std.errors
main		
overweight	0.0369	
	(0.0619)	
obese	0.3229***	
	(0.0678)	
age3544	0.3062***	
	(0.0756)	
age4554	0.5864***	
	(0.0761)	
age5564	1.0427***	
	(0.0814)	
age6574	1.2126***	
	(0.1100)	
age75plus	1.2876***	
	(0.1181)	
female	0.5003***	
	(0.0529)	
smoker	-0.1231*	
	(0.0716)	
married	0.1249**	
	(0.0551)	
black	0.0671	
	(0.1263)	
amind	-0.2324	
	(0.1523)	
asian	0.2979***	
	(0.0973)	
othrace	0.0000	
	(.)	
midwest	0.1819**	
	(0.0782)	
south	-0.0945	
	(0.0734)	
west	0.0073	
	(0.0818)	
lesshischool	0.0226	
	(0.0944)	
collegeplus	0.1566***	
	(0.0607)	
poor	-0.0452	
	(0.1657)	
nearpoor	0.0479	
	(0.2058)	

middleinc	-0.0508	
	(0.1004)	
highinc	-0.0993	
	(0.0979)	
_bs_1		1.5728e+10***
		(3.5014e+09)
_bs_2		0.0904***
		(0.0197)
_cons	5.8228***	
	(0.1581)	
No. of Obs.	11061.0000	11061.0000
R-Squared		

	(1)	(2)
	outpatient	
	Coef./std.errors	Coef./std.errors
main		
overweight	-0.1732	
	(0.1582)	
obese	0.0840	
	(0.1535)	
age3544	0.5268***	
	(0.1876)	
age4554	0.7082***	
	(0.1890)	
age5564	0.8239***	
	(0.2134)	
age6574	0.9152***	
	(0.2322)	
age75plus	0.7858***	
	(0.2364)	
female	0.2670*	
	(0.1372)	
smoker	0.1531	
	(0.1457)	
married	0.1104	
	(0.1512)	
black	0.4116**	
	(0.2007)	
amind	-1.2003***	
	(0.3286)	
asian	-0.0105	
	(0.1758)	
othrace	0.0000	
	(.)	
midwest	0.2457	
	(0.1941)	
south	-0.1839	
	(0.1747)	
west	-0.0131	
	(0.1845)	
lesshischool	-0.0184	
	(0.1497)	
collegeplus	0.1157	
	(0.1718)	
poor	-0.2468	
	(0.1828)	
nearpoor	-0.0964	
	(0.2321)	

middleinc	-0.0524	
	(0.2140)	
highinc	-0.3333	
	(0.3398)	
_bs_1		7.2993e+08
		(1.2765e+09)
_bs_2		0.0336
		(0.0592)
_cons	6.6930***	
	(0.2967)	
No. of Obs.	2245.0000	2245.0000
R-Squared		

	(1)	(2)
	outpatient	
	Coef./std.errors	Coef./std.errors
main		
overweight	0.0302	
	(0.0765)	
obese	0.2597***	
	(0.0840)	
age3544	0.6736*	
	(0.3753)	
age4554	0.8218**	
	(0.3465)	
age5564	1.1770***	
	(0.3366)	
age6574	0.5185	
	(0.3153)	
age75plus	0.6486**	
	(0.3159)	
female	-0.0409	
	(0.0678)	
smoker	-0.2658***	
	(0.1028)	
married	0.1387**	
	(0.0700)	
black	0.2236	
	(0.1716)	
amind	-0.5215**	
	(0.2288)	
asian	-0.1077	
	(0.1397)	
othrace	0.0000	
	(.)	
midwest	0.2064**	
	(0.0989)	
south	-0.0507	
	(0.0891)	
west	-0.0262	
	(0.1065)	
lesshischool	-0.1136	
	(0.0868)	
collegeplus	0.2711***	
	(0.0763)	
poor	0.1231	
	(0.1223)	
nearpoor	0.2382*	
	(0.1389)	

middleinc	0.2183**	
	(0.0964)	
highinc	0.2574***	
	(0.0979)	
_bs_1		6.3116e+09***
		(1.9492e+09)
_bs_2		0.0773***
		(0.0237)
_cons	6.7451***	
	(0.3587)	
No. of Obs.	3861.0000	3861.0000
R-Squared		

	(1)	(2)
	outpatient	
	Coef./std.errors	Coef./std.errors
main		
overweight	0.0142	
	(0.0959)	
obese	0.3695***	
	(0.1111)	
unins	-0.9198***	
	(0.1014)	
medicaid	0.4907***	
	(0.1732)	
medicare	0.4293	
	(0.5197)	
female	0.9185***	
	(0.0865)	
smoker	0.2206**	
	(0.1059)	
married	0.2741***	
	(0.0909)	
black	-0.0944	
	(0.1577)	
amind	-0.2763	
	(0.2246)	
asian	0.3910***	
	(0.1203)	
othrace	0.0000	
	(.)	
midwest	-0.0658	
	(0.1328)	
south	-0.1722	
	(0.1236)	
west	-0.1135	
	(0.1354)	
lesshischool	-0.0153	
	(0.1213)	
collegeplus	0.2936***	
	(0.1037)	
poor	0.1079	
	(0.1570)	
nearpoor	0.3025	
	(0.2235)	
middleinc	0.0277	
	(0.1330)	
highinc	0.1184	
	(0.1426)	

_bs_1		1.2489e+08
		(9.5384e+08)
_bs_2		0.0035
		(0.0268)
_cons	5.2827***	
	(0.2116)	
No. of Obs.	5848.0000	5848.0000
R-Squared		

	(1)	(2)
	outpatient	
	Coef./std.errors	Coef./std.errors
main		
overweight	-0.1027	
	(0.1214)	
obese	0.3668***	
	(0.1273)	
unins	-0.9986***	
	(0.1335)	
medicaid	0.4431*	
	(0.2318)	
medicare	0.6793**	
	(0.3429)	
female	0.4223***	
	(0.1016)	
smoker	0.1162	
	(0.1289)	
married	0.0224	
	(0.1064)	
black	0.2923	
	(0.1969)	
amind	-0.2888	
	(0.2394)	
asian	0.5741***	
	(0.1449)	
othrace	0.0000	
	(.)	
midwest	0.1421	
	(0.1536)	
south	-0.1137	
	(0.1412)	
west	0.0294	
	(0.1512)	
lesshischool	0.1792	
	(0.1666)	
collegeplus	0.3630***	
	(0.1177)	
poor	-0.4114*	
	(0.2172)	
nearpoor	-0.0688	
	(0.2989)	
middleinc	-0.2957*	
	(0.1713)	
highinc	-0.3230*	
	(0.1732)	

_bs_1		-9.4229e+08
		(1.3604e+09)
_bs_2		-0.0281
		(0.0400)
_cons	6.0538***	
	(0.2667)	
No. of Obs.	3885.0000	3885.0000
R-Squared		

	(1)	(2)
	outpatient	
	Coef./std.errors	Coef./std.errors
main		
overweight	0.2410**	
	(0.0992)	
obese	0.3459***	
	(0.1026)	
unins	-0.6880***	
	(0.1130)	
medicaid	0.4028*	
	(0.2118)	
medicare	0.7299***	
	(0.2166)	
female	0.4919***	
	(0.0832)	
smoker	-0.1525	
	(0.0970)	
married	-0.1352	
	(0.0909)	
black	0.2754	
	(0.1734)	
amind	-0.2429	
	(0.2489)	
asian	0.2545*	
	(0.1365)	
othrace	0.0000	
	(.)	
midwest	0.0364	
	(0.1269)	
south	-0.3025***	
	(0.1172)	
west	-0.2172*	
	(0.1273)	
lesshischool	0.0899	
	(0.1358)	
collegeplus	0.3007***	
	(0.0895)	
poor	0.1157	
	(0.1960)	
nearpoor	-0.2806	
	(0.2738)	
middleinc	0.0267	
	(0.1528)	
highinc	0.1562	
	(0.1518)	

_bs_1		3.4394e+09**
		(1.6131e+09)
_bs_2		0.0760**
		(0.0349)
_cons	6.3855***	
	(0.2253)	
No. of Obs.	3914.0000	3914.0000
R-Squared		

	(1)	(2)
	outpatient	
	Coef./std.errors	Coef./std.errors
main		
overweight	-0.2289**	
	(0.1112)	
obese	0.1651	
	(0.1167)	
unins	-0.5553***	
	(0.1455)	
medicaid	0.7424***	
	(0.2525)	
medicare	0.9900***	
	(0.1889)	
female	0.1156	
	(0.0942)	
smoker	-0.2064*	
	(0.1203)	
married	-0.0448	
	(0.1048)	
black	0.4019*	
	(0.2274)	
amind	0.0493	
	(0.2709)	
asian	0.7409***	
	(0.1774)	
othrace	0.0000	
	(.)	
midwest	0.2339*	
	(0.1390)	
south	0.2190*	
	(0.1285)	
west	0.0263	
	(0.1451)	
lesshischool	-0.1697	
	(0.1516)	
collegeplus	-0.0301	
	(0.1077)	
poor	-0.2492	
	(0.2258)	
nearpoor	-0.3373	
	(0.3019)	
middleinc	0.2169	
	(0.1818)	
highinc	0.2714	
	(0.1815)	

_bs_1		-4.1878e+09
		(2.8488e+09)
_bs_2		-0.0740
		(0.0487)
_cons	6.5496***	
	(0.2874)	
No. of Obs.	2892.0000	2892.0000
R-Squared		

	(1)	(2)
	outpatient	
	Coef./std.errors	Coef./std.errors
main		
overweight	-0.0755	
	(0.1208)	
obese	0.0432	
	(0.1272)	
female	-0.1212	
	(0.1025)	
smoker	-0.3685**	
	(0.1462)	
married	0.0991	
	(0.1068)	
black	-0.1306	
	(0.2609)	
amind	-0.8884***	
	(0.3162)	
asian	-0.2287	
	(0.2061)	
othrace	0.0000	
	(.)	
midwest	0.3851**	
	(0.1543)	
south	0.1601	
	(0.1359)	
west	0.1862	
	(0.1602)	
lesshischool	-0.1978	
	(0.1423)	
collegeplus	0.3143***	
	(0.1178)	
poor	0.2264	
	(0.2059)	
nearpoor	0.3622	
	(0.2287)	
middleinc	0.2927*	
	(0.1610)	
highinc	0.1678	
	(0.1587)	
_bs_1		-1.0200e+09
		(1.8158e+09)
_bs_2		-0.0296
		(0.0523)
_cons	7.4022***	
	(0.2872)	

No. of Obs.	1788.0000	1788.0000
R-Squared		

	(1)	(2)
	outpatient	
	Coef./std.errors	Coef./std.errors
main		
overweight	0.0811	
	(0.0987)	
obese	0.4183***	
	(0.1234)	
female	0.0520	
	(0.0956)	
smoker	-0.1391	
	(0.1883)	
married	0.1372	
	(0.0962)	
black	0.8850***	
	(0.2509)	
amind	0.1554	
	(0.3212)	
asian	0.4540**	
	(0.1995)	
othrace	0.0000	
	(.)	
midwest	0.0897	
	(0.1296)	
south	-0.1830	
	(0.1196)	
west	-0.3427**	
	(0.1396)	
lesshischool	-0.0646	
	(0.1145)	
collegeplus	0.2552**	
	(0.1054)	
poor	-0.0445	
	(0.1729)	
nearpoor	0.1577	
	(0.1831)	
middleinc	0.1259	
	(0.1222)	
highinc	0.3251**	
	(0.1272)	
_bs_1		9.5047e+08
		(1.1494e+09)
_bs_2		0.0280
		(0.0334)
_cons	6.8784***	
	(0.2669)	

No. of Obs.	1569.0000	1569.0000
R-Squared		

	(1)	(2)
	outpatient	
	Coef./std.errors	Coef./std.errors
main		
overweight	0.0138	
	(0.0514)	
obese	0.3065***	
	(0.0552)	
age3544	0.2910***	
	(0.0654)	
age4554	0.5794***	
	(0.0655)	
age5564	1.0383***	
	(0.0698)	
age6574	1.0941***	
	(0.0796)	
age75plus	1.1276***	
	(0.0819)	
female	0.4013***	
	(0.0440)	
smoker	-0.0517	
	(0.0592)	
married	0.0963**	
	(0.0460)	
black	0.1853*	
	(0.0986)	
amind	-0.3514***	
	(0.1254)	
asian	0.2157***	
	(0.0778)	
othrace	0.0000	
	(.)	
midwest	0.1591**	
	(0.0656)	
south	-0.1127*	
	(0.0605)	
west	-0.0402	
	(0.0674)	
lesshischool	0.0459	
	(0.0698)	
collegeplus	0.1872***	
	(0.0505)	
poor	0.1253	
	(0.1019)	
nearpoor	0.0567	
	(0.1316)	

middleinc	-0.0501	
	(0.0770)	
highinc	-0.0897	
	(0.0762)	
_bs_1		9.7423e+08
		(3.8834e+09)
_bs_2		0.0043
		(0.0173)
_cons	5.9813***	
	(0.1255)	
No. of Obs.	15175.0000	15175.0000
R-Squared		

	(1)	(2)
	outpatient	
	Coef./std.errors	Coef./std.errors
main		
overweight	0.0369	
	(0.0619)	
obese	0.3229***	
	(0.0678)	
age3544	0.3062***	
	(0.0756)	
age4554	0.5864***	
	(0.0761)	
age5564	1.0427***	
	(0.0814)	
age6574	1.2126***	
	(0.1100)	
age75plus	1.2876***	
	(0.1181)	
female	0.5003***	
	(0.0529)	
smoker	-0.1231*	
	(0.0716)	
married	0.1249**	
	(0.0551)	
black	0.0671	
	(0.1263)	
amind	-0.2324	
	(0.1523)	
asian	0.2979***	
	(0.0973)	
othrace	0.0000	
	(.)	
midwest	0.1819**	
	(0.0782)	
south	-0.0945	
	(0.0734)	
west	0.0073	
	(0.0818)	
lesshischool	0.0226	
	(0.0944)	
collegeplus	0.1566***	
	(0.0607)	
poor	-0.0452	
	(0.1657)	
nearpoor	0.0479	
	(0.2058)	

middleinc	-0.0508	
	(0.1004)	
highinc	-0.0993	
	(0.0979)	
_bs_1		2.0531e+09
		(3.4717e+09)
_bs_2		0.0118
		(0.0199)
_cons	5.8228***	
	(0.1581)	
No. of Obs.	11061.0000	11061.0000
R-Squared		

	(1)	(2)
	outpatient	
	Coef./std.errors	Coef./std.errors
main		
overweight	-0.1732	
	(0.1582)	
obese	0.0840	
	(0.1535)	
age3544	0.5268***	
	(0.1876)	
age4554	0.7082***	
	(0.1890)	
age5564	0.8239***	
	(0.2134)	
age6574	0.9152***	
	(0.2322)	
age75plus	0.7858***	
	(0.2364)	
female	0.2670*	
	(0.1372)	
smoker	0.1531	
	(0.1457)	
married	0.1104	
	(0.1512)	
black	0.4116**	
	(0.2007)	
amind	-1.2003***	
	(0.3286)	
asian	-0.0105	
	(0.1758)	
othrace	0.0000	
	(.)	
midwest	0.2457	
	(0.1941)	
south	-0.1839	
	(0.1747)	
west	-0.0131	
	(0.1845)	
lesshischool	-0.0184	
	(0.1497)	
collegeplus	0.1157	
	(0.1718)	
poor	-0.2468	
	(0.1828)	
nearpoor	-0.0964	
	(0.2321)	

middleinc	-0.0524	
	(0.2140)	
highinc	-0.3333	
	(0.3398)	
_bs_1		-1.0218e+09
		(9.5424e+08)
_bs_2		-0.0470
		(0.0427)
_cons	6.6930***	
	(0.2967)	
No. of Obs.	2245.0000	2245.0000
R-Squared		

	(1)	(2)
	outpatient	
	Coef./std.errors	Coef./std.errors
main		
overweight	0.0302	
	(0.0765)	
obese	0.2597***	
	(0.0840)	
age3544	0.6736*	
	(0.3753)	
age4554	0.8218**	
	(0.3465)	
age5564	1.1770***	
	(0.3366)	
age6574	0.5185	
	(0.3153)	
age75plus	0.6486**	
	(0.3159)	
female	-0.0409	
	(0.0678)	
smoker	-0.2658***	
	(0.1028)	
married	0.1387**	
	(0.0700)	
black	0.2236	
	(0.1716)	
amind	-0.5215**	
	(0.2288)	
asian	-0.1077	
	(0.1397)	
othrace	0.0000	
	(.)	
midwest	0.2064**	
	(0.0989)	
south	-0.0507	
	(0.0891)	
west	-0.0262	
	(0.1065)	
lesshischool	-0.1136	
	(0.0868)	
collegeplus	0.2711***	
	(0.0763)	
poor	0.1231	
	(0.1223)	
nearpoor	0.2382*	
	(0.1389)	

middleinc	0.2183**	
	(0.0964)	
highinc	0.2574***	
	(0.0979)	
_bs_1		8.3431e+08
		(2.1026e+09)
_bs_2		0.0102
		(0.0259)
_cons	6.7451***	
	(0.3587)	
No. of Obs.	3861.0000	3861.0000
R-Squared		

	(1)	(2)
	totalexp	
	Coef./std.errors	Coef./std.errors
main		
overweight	0.0204	
	(0.1061)	
obese	0.1951	
	(0.1228)	
unins	-0.8434***	
	(0.1164)	
medicaid	0.4269**	
	(0.1913)	
medicare	0.6232	
	(0.5753)	
female	1.0096***	
	(0.0973)	
smoker	0.1437	
	(0.1199)	
married	0.2498**	
	(0.1027)	
black	0.0538	
	(0.1722)	
amind	-0.2361	
	(0.2478)	
asian	0.4107***	
	(0.1325)	
othrace	0.0000	
	(.)	
midwest	-0.0612	
	(0.1470)	
south	-0.1024	
	(0.1365)	
west	-0.2088	
	(0.1502)	
lesshischool	-0.0119	
	(0.1351)	
collegeplus	-0.0885	
	(0.1164)	
poor	0.2753	
	(0.1754)	
nearpoor	0.4046	
	(0.2505)	
middleinc	-0.0789	
	(0.1462)	
highinc	0.0699	
	(0.1523)	

_bs_1		4.9464e+08
		(2.4561e+09)
_bs_2		0.0052
		(0.0255)
_cons	6.4661***	
	(0.2287)	
No. of Obs.	5848.0000	5848.0000
R-Squared		

	(1)	(2)
	totalexp	
	Coef./std.errors	Coef./std.errors
main		
overweight	-0.0652	
	(0.1092)	
obese	0.4905***	
	(0.1150)	
unins	-0.9031***	
	(0.1195)	
medicaid	0.6286***	
	(0.2090)	
medicare	1.0692***	
	(0.3111)	
female	0.3313***	
	(0.0901)	
smoker	0.2213*	
	(0.1173)	
married	0.0062	
	(0.0974)	
black	0.4106**	
	(0.1782)	
amind	0.2468	
	(0.2258)	
asian	0.5006***	
	(0.1335)	
othrace	0.0000	
	(.)	
midwest	0.1265	
	(0.1407)	
south	-0.1090	
	(0.1285)	
west	0.0233	
	(0.1370)	
lesshischool	0.1363	
	(0.1493)	
collegeplus	0.3816***	
	(0.1085)	
poor	-0.2008	
	(0.1948)	
nearpoor	0.2493	
	(0.2660)	
middleinc	-0.2557*	
	(0.1509)	
highinc	-0.1699	
	(0.1541)	

_bs_1		-1.3907e+09
		(2.7265e+09)
_bs_2		-0.0168
		(0.0328)
_cons	6.7818***	
	(0.2380)	
No. of Obs.	3885.0000	3885.0000
R-Squared		

	(1)	(2)
	totalexp	
	Coef./std.errors	Coef./std.errors
main		
overweight	0.2736***	
	(0.0978)	
obese	0.4029***	
	(0.1004)	
unins	-0.4409***	
	(0.1143)	
medicaid	0.8227***	
	(0.2011)	
medicare	1.2996***	
	(0.2083)	
female	0.3170***	
	(0.0802)	
smoker	-0.1883**	
	(0.0951)	
married	0.0048	
	(0.0897)	
black	0.4908***	
	(0.1718)	
amind	-0.3147	
	(0.2454)	
asian	0.5055***	
	(0.1334)	
othrace	0.0000	
	(.)	
midwest	-0.0125	
	(0.1239)	
south	-0.1594	
	(0.1157)	
west	-0.1043	
	(0.1235)	
lesshischool	0.0488	
	(0.1324)	
collegeplus	0.1263	
	(0.0887)	
poor	0.3230*	
	(0.1910)	
nearpoor	-0.1076	
	(0.2660)	
middleinc	-0.0738	
	(0.1506)	
highinc	-0.0281	
	(0.1494)	

_bs_1		1.0950e+10***
		(3.9940e+09)
_bs_2		0.0840***
		(0.0302)
_cons	7.1876***	
	(0.2230)	
No. of Obs.	3914.0000	3914.0000
R-Squared		

	(1)	(2)
	totalexp	
	Coef./std.errors	Coef./std.errors
main		
overweight	0.0466	
	(0.1011)	
obese	0.4555***	
	(0.1048)	
unins	-0.4590***	
	(0.1314)	
medicaid	0.6807***	
	(0.2220)	
medicare	0.8395***	
	(0.1676)	
female	0.0140	
	(0.0847)	
smoker	-0.0650	
	(0.1079)	
married	-0.0331	
	(0.0932)	
black	0.5263***	
	(0.1977)	
amind	0.0307	
	(0.2448)	
asian	0.5825***	
	(0.1589)	
othrace	0.0000	
	(.)	
midwest	0.2383*	
	(0.1273)	
south	0.2901**	
	(0.1155)	
west	0.1142	
	(0.1316)	
lesshischool	-0.0198	
	(0.1328)	
collegeplus	-0.1009	
	(0.0973)	
poor	0.2319	
	(0.2017)	
nearpoor	0.4341	
	(0.2767)	
middleinc	0.4838***	
	(0.1634)	
highinc	0.3803**	
	(0.1643)	

_bs_1		2.0933e+09
		(5.6080e+09)
_bs_2		0.0140
		(0.0370)
_cons	7.2520***	
	(0.2502)	
No. of Obs.	2892.0000	2892.0000
R-Squared		

	(1)	(2)
	totalexp	
	Coef./std.errors	Coef./std.errors
main		
overweight	-0.0473	
	(0.1254)	
obese	0.2849**	
	(0.1307)	
female	-0.0561	
	(0.1069)	
smoker	-0.0497	
	(0.1487)	
married	0.1208	
	(0.1126)	
black	-0.0729	
	(0.2560)	
amind	0.2613	
	(0.3338)	
asian	-0.0936	
	(0.2021)	
othrace	0.0000	
	(.)	
midwest	0.1737	
	(0.1571)	
south	0.1125	
	(0.1391)	
west	0.1414	
	(0.1663)	
lesshischool	-0.0983	
	(0.1434)	
collegeplus	0.0649	
	(0.1220)	
poor	0.3367	
	(0.2113)	
nearpoor	0.4192*	
	(0.2313)	
middleinc	0.0154	
	(0.1632)	
highinc	-0.0253	
	(0.1647)	
_bs_1		-1.7994e+09
		(4.6066e+09)
_bs_2		-0.0165
		(0.0423)
_cons	8.5477***	
	(0.2932)	

No. of Obs.	1788.0000	1788.0000
R-Squared		

	(1)	(2)
	totalexp	
	Coef./std.errors	Coef./std.errors
main		
overweight	-0.0792	
	(0.0941)	
obese	0.1280	
	(0.1198)	
female	0.0238	
	(0.0951)	
smoker	0.0647	
	(0.1860)	
married	-0.0816	
	(0.0934)	
black	0.3679	
	(0.2431)	
amind	0.2020	
	(0.3113)	
asian	0.2700	
	(0.1911)	
othrace	0.0000	
	(.)	
midwest	-0.1310	
	(0.1262)	
south	0.1188	
	(0.1177)	
west	-0.4036***	
	(0.1381)	
lesshischool	0.0919	
	(0.1141)	
collegeplus	-0.0797	
	(0.1030)	
poor	-0.0712	
	(0.1664)	
nearpoor	0.1591	
	(0.1750)	
middleinc	-0.0038	
	(0.1203)	
highinc	0.1991	
	(0.1266)	
_bs_1		-3.4151e+09
		(4.4073e+09)
_bs_2		-0.0284
		(0.0365)
_cons	8.6934***	
	(0.2599)	

No. of Obs.	1569.0000	1569.0000
R-Squared		

	(1)	(2)
	totalexp	
	Coef./std.errors	Coef./std.errors
main		
overweight	0.0437	
	(0.0515)	
obese	0.3912***	
	(0.0555)	
age3544	0.2313***	
	(0.0661)	
age4554	0.6339***	
	(0.0664)	
age5564	1.0313***	
	(0.0708)	
age6574	1.2251***	
	(0.0801)	
age75plus	1.4062***	
	(0.0825)	
female	0.3588***	
	(0.0444)	
smoker	-0.0141	
	(0.0599)	
married	0.0980**	
	(0.0464)	
black	0.1814*	
	(0.0990)	
amind	-0.0161	
	(0.1264)	
asian	0.2357***	
	(0.0776)	
othrace	0.0000	
	(.)	
midwest	0.0947	
	(0.0658)	
south	0.0122	
	(0.0609)	
west	-0.0515	
	(0.0676)	
lesshischool	0.1138	
	(0.0697)	
collegeplus	0.0402	
	(0.0513)	
poor	0.3350***	
	(0.1019)	
nearpoor	0.3041**	
	(0.1315)	

middleinc	-0.1079	
	(0.0769)	
highinc	-0.1510**	
	(0.0762)	
_bs_1		8.7160e+09
		(1.0735e+10)
_bs_2		0.0135
		(0.0167)
_cons	6.9718***	
	(0.1230)	
No. of Obs.	15175.0000	15175.0000
R-Squared		

	(1)	(2)
	totalexp	
	Coef./std.errors	Coef./std.errors
main		
overweight	0.0715	
	(0.0635)	
obese	0.4105***	
	(0.0693)	
age3544	0.2066***	
	(0.0781)	
age4554	0.5940***	
	(0.0790)	
age5564	1.0117***	
	(0.0843)	
age6574	1.3429***	
	(0.1133)	
age75plus	1.5228***	
	(0.1210)	
female	0.4655***	
	(0.0548)	
smoker	-0.1295*	
	(0.0739)	
married	0.1774***	
	(0.0565)	
black	0.1548	
	(0.1292)	
amind	0.1423	
	(0.1568)	
asian	0.3374***	
	(0.0991)	
othrace	0.0000	
	(.)	
midwest	0.1083	
	(0.0801)	
south	-0.0122	
	(0.0753)	
west	-0.0616	
	(0.0835)	
lesshischool	0.0662	
	(0.0960)	
collegeplus	0.0395	
	(0.0629)	
poor	0.0677	
	(0.1683)	
nearpoor	0.1552	
	(0.2074)	

middleinc	-0.0577	
	(0.1022)	
highinc	-0.0999	
	(0.1004)	
_bs_1		1.0300e+10
		(9.4759e+09)
_bs_2		0.0225
		(0.0206)
_cons	6.7254***	
	(0.1568)	
No. of Obs.	11061.0000	11061.0000
R-Squared		

	(1)	(2)
	totalexp	
	Coef./std.errors	Coef./std.errors
main		
overweight	-0.0094	
	(0.1098)	
obese	0.3012***	
	(0.1064)	
age3544	0.4635***	
	(0.1293)	
age4554	1.0192***	
	(0.1355)	
age5564	1.0542***	
	(0.1555)	
age6574	1.0825***	
	(0.1596)	
age75plus	0.9112***	
	(0.1694)	
female	-0.0296	
	(0.0994)	
smoker	0.1052	
	(0.1034)	
married	-0.1654	
	(0.1076)	
black	0.2258	
	(0.1466)	
amind	-0.8830***	
	(0.2382)	
asian	0.1188	
	(0.1253)	
othrace	0.0000	
	(.)	
midwest	0.2749**	
	(0.1373)	
south	-0.0420	
	(0.1253)	
west	0.1612	
	(0.1264)	
lesshischool	0.0023	
	(0.1036)	
collegeplus	0.0635	
	(0.1217)	
poor	0.1436	
	(0.1255)	
nearpoor	0.3095*	
	(0.1620)	

middleinc	0.1170	
	(0.1466)	
highinc	0.1277	
	(0.2411)	
_bs_1		-1.9692e+08
		(2.6372e+09)
_bs_2		-0.0024
		(0.0323)
_cons	7.6531***	
	(0.2081)	
No. of Obs.	2245.0000	2245.0000
R-Squared		

	(1)	(2)
	totalexp	
	Coef./std.errors	Coef./std.errors
main		
overweight	-0.0657	
	(0.0770)	
obese	0.2678***	
	(0.0860)	
age3544	0.7813**	
	(0.3779)	
age4554	1.0993***	
	(0.3495)	
age5564	1.0644***	
	(0.3381)	
age6574	0.5049	
	(0.3162)	
age75plus	0.7377**	
	(0.3184)	
female	-0.0300	
	(0.0690)	
smoker	-0.0258	
	(0.1034)	
married	0.0504	
	(0.0722)	
black	0.0461	
	(0.1697)	
amind	0.1830	
	(0.2333)	
asian	-0.0180	
	(0.1376)	
othrace	0.0000	
	(.)	
midwest	0.0293	
	(0.0993)	
south	0.1050	
	(0.0899)	
west	-0.0513	
	(0.1080)	
lesshischool	0.0082	
	(0.0875)	
collegeplus	-0.0242	
	(0.0777)	
poor	0.0955	
	(0.1218)	
nearpoor	0.3156**	
	(0.1392)	

middleinc	0.0080	
	(0.0970)	
highinc	0.1428	
	(0.1001)	
_bs_1		-5.9508e+09
		(6.4686e+09)
_bs_2		-0.0213
		(0.0231)
_cons	8.0851***	
	(0.3548)	
No. of Obs.	3861.0000	3861.0000
R-Squared		
