# Supplementary material for: Changes in healthcare spending attributable to obesity and overweight: payer- and service-specific estimates
Source: BMC Public Health. 2022 May 13;22:962. doi: 10.1186/s12889-022-13176-y (PMC9101934; doi:10.1186/s12889-022-13176-y)
Supplement: Supplementary file 2 — Additional file 2. 2016 Full regression Results. [file 12889_2022_13176_MOESM2_ESM.pdf]

	(1)	(2)
	inpatient	
	Coef./std.errors	Coef./std.errors
main		
overweight	0.1641	
	(0.1379)	
obese	-0.0135	
	(0.1392)	
unins	-0.3306*	
	(0.1872)	
medicaid	0.7268***	
	(0.1631)	
medicare	0.8981**	
	(0.3834)	
female	1.8191***	
	(0.1684)	
smoker	-0.2829*	
	(0.1713)	
married	0.8744***	
	(0.1253)	
black	-0.0551	
	(0.1453)	
amind	0.2683	
	(0.6320)	
asian	-0.0949	
	(0.2289)	
othrace	-0.2193	
	(0.3167)	
midwest	0.4168**	
	(0.1860)	
south	0.2065	
	(0.1740)	
west	-0.1580	
	(0.1884)	
lesshischool	-0.2667	
	(0.1627)	
collegeplus	-0.0151	
	(0.1367)	
poor	0.6960***	
	(0.1700)	
nearpoor	-0.1683	
	(0.2726)	
middleinc	-0.4233**	
	(0.1951)	
highinc	-0.3488*	
	(0.2119)	

_bs_1		-3.3249e+09
		(3.1876e+09)
_bs_2		-0.0827
		(0.0761)
_cons	-4.6144***	
	(0.2913)	
glm		
overweight	-391.4874	
	(2524.5990)	
obese	-3358.2167	
	(2533.5858)	
unins	1931.0648	
	(3625.6758)	
medicaid	1607.1043	
	(3152.5995)	
medicare	21549.7469***	
	(6663.6651)	
female	-5219.2791	
	(3401.5068)	
smoker	2368.8328	
	(3120.0699)	
married	-901.9378	
	(2531.5460)	
black	441.7747	
	(2627.8251)	
amind	5765.9183	
	(11542.6336)	
asian	-3482.0310	
	(4323.7412)	
othrace	-5882.7376	
	(5903.7918)	
midwest	-5889.2289*	
	(3416.7197)	
south	-4642.4160	
	(3244.0177)	
west	-3858.1026	
	(3509.7962)	
lesshischool	-37.1351	
	(2969.2443)	
collegeplus	276.7460	
	(2570.1213)	
poor	709.9060	
	(3143.0255)	
nearpoor	-3908.1159	
	(5096.1782)	
middleinc	6912.1575*	

	(3827.9773)	
highinc	7555.6833*	
	(4192.9226)	
_cons	17466.0135***	
	(5442.5835)	
No. of Obs.	6003.0000	6003.0000
R-Squared		

	(1)	(2)
	inpatient	
	Coef./std.errors	Coef./std.errors
main		
overweight	0.0523	
	(0.1960)	
obese	0.0448	
	(0.1901)	
unins	0.0726	
	(0.2308)	
medicaid	0.6261***	
	(0.2323)	
medicare	1.5392***	
	(0.2890)	
female	0.5248***	
	(0.1648)	
smoker	0.2435	
	(0.2005)	
married	0.1666	
	(0.1642)	
black	-0.4966**	
	(0.2200)	
amind	-1.4320	
	(1.0599)	
asian	-0.3752	
	(0.3087)	
othrace	-0.1529	
	(0.4434)	
midwest	0.2615	
	(0.2898)	
south	0.5799**	
	(0.2601)	
west	0.2975	
	(0.2697)	
lesshischool	-0.2281	
	(0.2305)	
collegeplus	0.2896	
	(0.1873)	
poor	0.6695**	
	(0.2702)	
nearpoor	0.7161**	
	(0.3385)	
middleinc	0.3556	
	(0.2672)	
highinc	0.2496	
	(0.2901)	

_bs_1		-8.7395e+08
		(2.9600e+09)
_bs_2		-0.0282
		(0.0946)
_cons	-4.2975***	
	(0.4278)	
glm		
overweight	578.7015	
	(3890.3042)	
obese	-1989.1176	
	(3692.2765)	
unins	-1275.3493	
	(4573.8666)	
medicaid	-2468.7091	
	(4192.3252)	
medicare	10631.9424**	
	(4999.7143)	
female	-2207.3203	
	(3150.0022)	
smoker	216.6305	
	(3988.6425)	
married	-5147.8491	
	(3293.0477)	
black	-5535.2884	
	(4235.2011)	
amind	-10658.6154	
	(20481.2934)	
asian	-2678.8126	
	(6044.3735)	
othrace	-7442.5942	
	(8399.9883)	
midwest	1521.1412	
	(5545.9762)	
south	1449.9104	
	(5000.6107)	
west	3346.0409	
	(5234.3123)	
lesshischool	-1416.5722	
	(4435.0461)	
collegeplus	3045.3533	
	(3385.5266)	
poor	1112.5433	
	(5421.9261)	
nearpoor	-3361.2569	
	(6538.1343)	
middleinc	6532.3915	

	(5364.3070)	
highinc	2936.1370	
	(5691.7229)	
_cons	16511.2452**	
	(8357.8918)	
No. of Obs.	3609.0000	3609.0000
R-Squared		

	(1)	(2)
	inpatient	
	Coef./std.errors	Coef./std.errors
main		
overweight	0.0522	
	(0.2112)	
obese	0.3003	
	(0.1998)	
unins	-0.6464**	
	(0.2659)	
medicaid	0.3322	
	(0.2148)	
medicare	0.8786***	
	(0.2342)	
female	0.6101***	
	(0.1640)	
smoker	-0.0067	
	(0.1950)	
married	-0.2846*	
	(0.1689)	
black	-0.2158	
	(0.1941)	
amind	-0.1869	
	(1.0339)	
asian	-0.1634	
	(0.3457)	
othrace	0.4212	
	(0.4012)	
midwest	0.0462	
	(0.2385)	
south	0.0376	
	(0.2124)	
west	-0.5042**	
	(0.2497)	
lesshischool	0.0202	
	(0.2121)	
collegeplus	-0.1514	
	(0.1790)	
poor	0.0784	
	(0.2455)	
nearpoor	-0.1851	
	(0.3669)	
middleinc	-0.1369	
	(0.2330)	
highinc	-0.3621	
	(0.2546)	

_bs_1		9.8614e+09**
		(4.6238e+09)
_bs_2		0.2407**
		(0.1078)
_cons	-2.9520***	
	(0.3543)	
glm		
overweight	4868.5274	
	(6524.1937)	
obese	7743.7505	
	(6061.3125)	
unins	-11227.4818	
	(8299.8400)	
medicaid	-15236.3739**	
	(6404.5958)	
medicare	106.4320	
	(6659.1093)	
female	-5160.8221	
	(5039.5647)	
smoker	519.2615	
	(5905.8152)	
married	-9827.0313*	
	(5327.8644)	
black	4233.5092	
	(5943.8592)	
amind	-12994.2335	
	(32287.2769)	
asian	-4513.6362	
	(10794.1328)	
othrace	18859.8587	
	(11929.1323)	
midwest	-9075.0166	
	(7286.1500)	
south	-6648.4172	
	(6483.7964)	
west	2149.0765	
	(7882.4216)	
lesshischool	4070.8278	
	(6695.5717)	
collegeplus	11846.6911**	
	(5467.1531)	
poor	-137.6396	
	(7362.0981)	
nearpoor	-8036.6157	
	(11148.5031)	
middleinc	-3762.0850	

	(7169.3835)	
highinc	-2846.4830	
	(7936.5028)	
_cons	29805.3393***	
	(10415.3659)	
No. of Obs.	3652.0000	3652.0000
R-Squared		

	(1)	(2)
	inpatient	
	Coef./std.errors	Coef./std.errors
main		
overweight	-0.2625	
	(0.1801)	
obese	0.1002	
	(0.1689)	
unins	-0.2196	
	(0.2580)	
medicaid	0.6957***	
	(0.1807)	
medicare	1.0369***	
	(0.1621)	
female	-0.0047	
	(0.1388)	
smoker	0.2747*	
	(0.1644)	
married	-0.0899	
	(0.1508)	
black	0.1655	
	(0.1605)	
amind	-0.8512	
	(1.0344)	
asian	-0.1806	
	(0.3487)	
othrace	0.2422	
	(0.3797)	
midwest	0.1179	
	(0.2090)	
south	-0.0881	
	(0.1940)	
west	-0.1945	
	(0.2165)	
lesshischool	-0.0283	
	(0.1899)	
collegeplus	0.2870*	
	(0.1578)	
poor	0.1536	
	(0.2105)	
nearpoor	-0.2876	
	(0.3326)	
middleinc	-0.1616	
	(0.2152)	
highinc	-0.3351	
	(0.2324)	

_bs_1		-3.3248e+09
		(6.8714e+09)
_bs_2		-0.0508
		(0.1035)
_cons	-2.7485***	
	(0.3164)	
glm		
overweight	-1107.0723	
	(4717.6241)	
obese	-5744.6907	
	(4330.7822)	
unins	142.8105	
	(6895.8863)	
medicaid	-8121.8299*	
	(4530.6711)	
medicare	-6427.7576	
	(4045.4466)	
female	2298.1761	
	(3595.2751)	
smoker	-456.3687	
	(4380.6724)	
married	8154.2401*	
	(4185.1934)	
black	212.4645	
	(4022.3443)	
amind	-17440.4036	
	(27878.0165)	
asian	-18642.0964**	
	(9232.0893)	
othrace	-714.9842	
	(9618.1798)	
midwest	-5583.9283	
	(5439.0387)	
south	-6472.1873	
	(5030.5630)	
west	-427.9161	
	(5685.6240)	
lesshischool	3739.6139	
	(4940.1871)	
collegeplus	4451.8055	
	(4151.6124)	
poor	-142.5480	
	(5333.8136)	
nearpoor	-7427.6623	
	(8664.3927)	
middleinc	-27.1970	

	(5492.0452)	
highinc	-3606.8024	
	(6104.6425)	
_cons	30683.5370***	
	(8389.7270)	
No. of Obs.	3353.0000	3353.0000
R-Squared		

	(1)	(2)
	inpatient	
	Coef./std.errors	Coef./std.errors
main		
overweight	0.2030	
	(0.1703)	
obese	0.2306	
	(0.1714)	
female	0.0625	
	(0.1360)	
smoker	0.4135**	
	(0.1845)	
married	-0.3582**	
	(0.1437)	
black	-0.0297	
	(0.1748)	
amind	0.6083	
	(0.6573)	
asian	-0.2935	
	(0.3212)	
othrace	-0.3379	
	(0.4804)	
midwest	0.1274	
	(0.2186)	
south	0.1298	
	(0.1960)	
west	-0.3049	
	(0.2271)	
lesshischool	0.0608	
	(0.1939)	
collegeplus	0.2686*	
	(0.1628)	
poor	-0.2934	
	(0.2428)	
nearpoor	0.3434	
	(0.2887)	
middleinc	-0.2061	
	(0.2032)	
highinc	-0.3228	
	(0.2098)	
_bs_1		4.5793e+09
		(4.1329e+09)
_bs_2		0.0810
		(0.0717)
_cons	-2.0278***	
	(0.2964)	

glm		
overweight	2853.8682	
	(3552.3007)	
obese	887.3801	
	(3452.4311)	
female	-239.7047	
	(2770.3067)	
smoker	-4025.5523	
	(3752.5214)	
married	-467.9081	
	(2946.9202)	
black	-1519.1410	
	(3404.4166)	
amind	1985.6599	
	(12734.4758)	
asian	5357.7238	
	(6600.0997)	
othrace	7049.1023	
	(9924.3713)	
midwest	-546.6661	
	(4507.5557)	
south	77.5321	
	(4019.0285)	
west	2705.7450	
	(4835.2789)	
lesshischool	-1198.5243	
	(3907.8267)	
collegeplus	-6176.0909*	
	(3293.5594)	
poor	5528.8371	
	(4904.4178)	
nearpoor	2292.1699	
	(5560.5696)	
middleinc	8258.2232**	
	(4113.3533)	
highinc	5861.1050	
	(4303.4706)	
_cons	16411.2265***	
	(5865.3895)	
No. of Obs.	2309.0000	2309.0000
R-Squared		

	(1)	(2)
	inpatient	
	Coef./std.errors	Coef./std.errors
main		
overweight	0.1718	
	(0.1627)	
obese	0.4618***	
	(0.1758)	
female	-0.0996	
	(0.1479)	
smoker	-0.2770	
	(0.4153)	
married	-0.2069	
	(0.1534)	
black	-0.1052	
	(0.1999)	
amind	0.6259	
	(0.6935)	
asian	-1.3503***	
	(0.4735)	
othrace	-0.3491	
	(0.7619)	
midwest	-0.0528	
	(0.2170)	
south	0.0084	
	(0.1889)	
west	0.1205	
	(0.2111)	
lesshischool	0.0578	
	(0.1770)	
collegeplus	-0.3439**	
	(0.1672)	
poor	0.0323	
	(0.2272)	
nearpoor	0.2964	
	(0.3009)	
middleinc	0.2612	
	(0.2010)	
highinc	0.1550	
	(0.2168)	
_bs_1		7.0336e+09**
		(2.9982e+09)
_bs_2		0.1460**
		(0.0609)
_cons	-1.6675***	
	(0.2752)	

glm		
overweight	1000.0608	
	(3052.5234)	
obese	3556.7138	
	(3301.1689)	
female	-3886.7867	
	(2791.7741)	
smoker	-3819.6966	
	(8029.9174)	
married	-1059.6734	
	(3002.4022)	
black	-1180.2407	
	(3735.1474)	
amind	-15863.6516	
	(12125.8145)	
asian	-8807.9483	
	(9423.6021)	
othrace	-6890.7714	
	(14774.8247)	
midwest	-1398.6981	
	(4046.3570)	
south	-2155.0455	
	(3549.7179)	
west	5405.6448	
	(3938.2869)	
lesshischool	5673.2746*	
	(3302.2334)	
collegeplus	3974.9115	
	(3163.1743)	
poor	-2483.8784	
	(4274.1909)	
nearpoor	-2065.3277	
	(5561.7259)	
middleinc	-3304.0186	
	(3922.6660)	
highinc	-2042.2455	
	(4050.4232)	
_cons	17602.1801***	
	(5577.9757)	
No. of Obs.	1640.0000	1640.0000
R-Squared		

	(1)	(2)
	inpatient	
	Coef./std.errors	Coef./std.errors
main		
overweight	0.0464	
	(0.0718)	
obese	0.2066***	
	(0.0708)	
age3544	-0.1680	
	(0.1030)	
age4554	-0.1363	
	(0.1001)	
age5564	0.2197**	
	(0.0919)	
age6574	0.6304***	
	(0.0901)	
age75plus	0.9587***	
	(0.0921)	
female	0.4871***	
	(0.0604)	
smoker	0.2139***	
	(0.0802)	
married	0.0390	
	(0.0625)	
black	-0.0356	
	(0.0734)	
amind	0.0187	
	(0.3414)	
asian	-0.4037***	
	(0.1320)	
othrace	-0.0558	
	(0.1753)	
midwest	0.1424	
	(0.0904)	
south	0.0507	
	(0.0818)	
west	-0.1719*	
	(0.0915)	
lesshischool	-0.0384	
	(0.0806)	
collegeplus	0.0159	
	(0.0670)	
poor	0.4383***	
	(0.0913)	
nearpoor	0.1568	
	(0.1335)	

middleinc	-0.2145**	
	(0.0891)	
highinc	-0.4137***	
	(0.0934)	
_bs_1		1.7026e+10*
		(9.2021e+09)
_bs_2		0.0625*
		(0.0338)
_cons	-2.9328***	
	(0.1321)	
glm		
overweight	1288.5776	
	(1600.6419)	
obese	176.0602	
	(1557.4357)	
age3544	3567.7275	
	(2335.3043)	
age4554	9775.5514***	
	(2254.9131)	
age5564	11178.4394***	
	(2077.5825)	
age6574	5315.5822***	
	(2050.5221)	
age75plus	2940.4923	
	(2077.9635)	
female	-2078.6191	
	(1395.0294)	
smoker	-768.7965	
	(1811.2373)	
married	-822.3417	
	(1417.6979)	
black	74.3313	
	(1602.6946)	
amind	-3053.4765	
	(7447.2250)	
asian	-4012.1297	
	(3002.9835)	
othrace	3178.4434	
	(3900.4726)	
midwest	-4655.1744**	
	(1990.8601)	
south	-3191.5948*	
	(1809.1519)	
west	532.5519	
	(2034.2688)	
lesshischool	2640.4250	

	(1764.2357)	
collegeplus	2523.8087*	
	(1487.1015)	
poor	-1323.0905	
	(2016.9226)	
nearpoor	-3542.9993	
	(2919.8651)	
middleinc	3048.0068	
	(1986.3387)	
highinc	2729.1593	
	(2090.4483)	
_cons	14146.5026***	
	(3053.8433)	
No. of Obs.	17196.0000	17196.0000
R-Squared		

	(1)	(2)
	inpatient	
	Coef./std.errors	Coef./std.errors
main		
overweight	0.0205	
	(0.1052)	
obese	0.3035***	
	(0.1043)	
age3544	-0.1575	
	(0.1399)	
age4554	-0.1674	
	(0.1381)	
age5564	0.0910	
	(0.1335)	
age6574	0.6653***	
	(0.1443)	
age75plus	1.3147***	
	(0.1432)	
female	0.5888***	
	(0.0873)	
smoker	0.0279	
	(0.1434)	
married	0.3459***	
	(0.0918)	
black	-0.0590	
	(0.1202)	
amind	0.4310	
	(0.5286)	
asian	-0.0856	
	(0.1605)	
othrace	0.0156	
	(0.2571)	
midwest	-0.0353	
	(0.1327)	
south	-0.0236	
	(0.1228)	
west	-0.2072	
	(0.1361)	
lesshischool	-0.0083	
	(0.1520)	
collegeplus	0.0666	
	(0.0969)	
poor	0.1930	
	(0.1944)	
nearpoor	0.0858	
	(0.2749)	

middleinc	-0.1220	
	(0.1364)	
highinc	-0.2741**	
	(0.1373)	
_bs_1		1.4972e+10*
		(7.7396e+09)
_bs_2		0.0879**
		(0.0446)
_cons	-3.4249***	
	(0.2070)	
glm		
overweight	752.3977	
	(2515.2907)	
obese	32.0338	
	(2499.7390)	
age3544	4765.7158	
	(3369.0131)	
age4554	15240.7460***	
	(3305.8907)	
age5564	18602.8074***	
	(3231.4261)	
age6574	5434.0670	
	(3482.4903)	
age75plus	3144.7592	
	(3425.9951)	
female	-3276.5865	
	(2160.5240)	
smoker	2526.2321	
	(3466.1274)	
married	-2219.4602	
	(2244.6451)	
black	-2170.8368	
	(2879.5370)	
amind	-7950.3296	
	(12428.9529)	
asian	-4808.7685	
	(3880.9894)	
othrace	1219.0099	
	(6230.6698)	
midwest	-5566.5183*	
	(3155.1495)	
south	-5677.3015*	
	(2929.0091)	
west	476.2371	
	(3236.2827)	
lesshischool	5763.7376	

	(3588.4235)	
collegeplus	5294.5318**	
	(2326.1911)	
poor	678.8772	
	(4581.5360)	
nearpoor	-4220.5826	
	(6468.7403)	
middleinc	391.8166	
	(3257.5235)	
highinc	-1241.9206	
	(3285.3796)	
_cons	17485.2045***	
	(5339.0021)	
No. of Obs.	11222.0000	11222.0000
R-Squared		

	(1)	(2)
	inpatient	
	Coef./std.errors	Coef./std.errors
main		
overweight	-0.0184	
	(0.1256)	
obese	-0.0335	
	(0.1210)	
age3544	-0.2660	
	(0.1640)	
age4554	-0.1371	
	(0.1624)	
age5564	0.3728***	
	(0.1433)	
age6574	0.6648***	
	(0.1688)	
age75plus	0.7066***	
	(0.1799)	
female	0.6100***	
	(0.1141)	
smoker	0.0871	
	(0.1185)	
married	-0.0743	
	(0.1234)	
black	-0.1222	
	(0.1132)	
amind	-1.4525	
	(1.0248)	
asian	-0.8137***	
	(0.2679)	
othrace	-0.1693	
	(0.2795)	
midwest	0.3883**	
	(0.1508)	
south	0.3190**	
	(0.1355)	
west	-0.3399**	
	(0.1473)	
lesshischool	-0.0845	
	(0.1171)	
collegeplus	0.1001	
	(0.1271)	
poor	0.2252*	
	(0.1299)	
nearpoor	-0.0924	
	(0.1893)	

middleinc	-0.1271	
	(0.1722)	
highinc	-0.3030	
	(0.3063)	
_bs_1		-4.7837e+08
		(3.3271e+09)
_bs_2		-0.0091
		(0.0628)
_cons	-2.4546***	
	(0.2029)	
glm		
overweight	4630.8584	
	(2824.0484)	
obese	5.1181	
	(2712.1932)	
age3544	2915.6094	
	(3807.0962)	
age4554	2385.4156	
	(3727.2299)	
age5564	4938.5405	
	(3301.8311)	
age6574	5023.4514	
	(3891.6128)	
age75plus	3814.0517	
	(4037.7097)	
female	-1198.7510	
	(2705.7152)	
smoker	1480.4665	
	(2724.1426)	
married	-2180.0988	
	(2758.9566)	
black	-205.8441	
	(2511.8396)	
amind	-4927.1294	
	(24398.4671)	
asian	-2474.9894	
	(6218.5735)	
othrace	-495.9784	
	(6275.8189)	
midwest	-5631.2814*	
	(3390.2672)	
south	-1853.6195	
	(3018.7275)	
west	-1711.0233	
	(3365.4281)	
lesshischool	883.7690	

	(2631.1152)	
collegeplus	-876.3627	
	(2820.7116)	
poor	-1334.0044	
	(2910.3403)	
nearpoor	-3354.3068	
	(4249.2817)	
middleinc	8086.3213**	
	(3960.1160)	
highinc	13141.6291*	
	(7013.4594)	
_cons	13817.9197***	
	(4758.5488)	
No. of Obs.	3772.0000	3772.0000
R-Squared		

	(1)	(2)
	inpatient	
	Coef./std.errors	Coef./std.errors
main		
overweight	0.1154	
	(0.1072)	
obese	0.1906*	
	(0.1097)	
age3544	0.5315	
	(0.4299)	
age4554	0.1224	
	(0.3980)	
age5564	0.4034	
	(0.3673)	
age6574	-0.0857	
	(0.3556)	
age75plus	0.2430	
	(0.3569)	
female	0.0748	
	(0.0888)	
smoker	0.3317***	
	(0.1267)	
married	-0.2437***	
	(0.0945)	
black	-0.0542	
	(0.1125)	
amind	-0.0417	
	(0.4556)	
asian	-0.4978**	
	(0.2399)	
othrace	-0.1877	
	(0.2972)	
midwest	0.0716	
	(0.1361)	
south	0.0515	
	(0.1208)	
west	-0.1478	
	(0.1409)	
lesshischool	0.1113	
	(0.1149)	
collegeplus	0.0763	
	(0.1019)	
poor	-0.0352	
	(0.1340)	
nearpoor	0.0589	
	(0.1841)	

middleinc	-0.0351	
	(0.1262)	
highinc	-0.1779	
	(0.1371)	
_bs_1		1.0289e+10*
		(5.7031e+09)
_bs_2		0.0804*
		(0.0434)
_cons	-1.9117***	
	(0.3852)	
glm		
overweight	3223.6035	
	(2225.1562)	
obese	1665.7601	
	(2237.0545)	
age3544	-5800.7395	
	(8698.5269)	
age4554	-7530.8476	
	(8171.1207)	
age5564	-12364.0650	
	(7577.4995)	
age6574	-12639.1432*	
	(7437.9768)	
age75plus	-14970.0130**	
	(7489.7610)	
female	-880.3699	
	(1839.3067)	
smoker	-511.3810	
	(2623.5606)	
married	-1299.9772	
	(1978.3760)	
black	657.6487	
	(2271.9367)	
amind	-5626.6795	
	(9289.1570)	
asian	671.9345	
	(5131.0400)	
othrace	5114.3179	
	(6186.2085)	
midwest	-379.5060	
	(2797.6047)	
south	1917.2964	
	(2472.6081)	
west	5601.5573*	
	(2935.3715)	
lesshischool	3127.9792	

	(2353.7386)	
collegeplus	979.6703	
	(2099.9814)	
poor	609.7110	
	(2725.0952)	
nearpoor	-2125.6033	
	(3722.2059)	
middleinc	3187.9857	
	(2614.5068)	
highinc	2323.4910	
	(2823.3175)	
_cons	26154.6249***	
	(8178.8433)	
No. of Obs.	4547.0000	4547.0000
R-Squared		

	(1)	(2)
	inpatient	
	Coef./std.errors	Coef./std.errors
main		
overweight	0.1641	
	(0.1379)	
obese	-0.0135	
	(0.1392)	
unins	-0.3306*	
	(0.1872)	
medicaid	0.7268***	
	(0.1631)	
medicare	0.8981**	
	(0.3834)	
female	1.8191***	
	(0.1684)	
smoker	-0.2829*	
	(0.1713)	
married	0.8744***	
	(0.1253)	
black	-0.0551	
	(0.1453)	
amind	0.2683	
	(0.6320)	
asian	-0.0949	
	(0.2289)	
othrace	-0.2193	
	(0.3167)	
midwest	0.4168**	
	(0.1860)	
south	0.2065	
	(0.1740)	
west	-0.1580	
	(0.1884)	
lesshischool	-0.2667	
	(0.1627)	
collegeplus	-0.0151	
	(0.1367)	
poor	0.6960***	
	(0.1700)	
nearpoor	-0.1683	
	(0.2726)	
middleinc	-0.4233**	
	(0.1951)	
highinc	-0.3488*	
	(0.2119)	

_bs_1		1.3628e+09
		(2.6367e+09)
_bs_2		0.0339
		(0.0658)
_cons	-4.6144***	
	(0.2913)	
glm		
overweight	-391.4874	
	(2524.5990)	
obese	-3358.2167	
	(2533.5858)	
unins	1931.0648	
	(3625.6758)	
medicaid	1607.1043	
	(3152.5995)	
medicare	21549.7469***	
	(6663.6651)	
female	-5219.2791	
	(3401.5068)	
smoker	2368.8328	
	(3120.0699)	
married	-901.9378	
	(2531.5460)	
black	441.7747	
	(2627.8251)	
amind	5765.9183	
	(11542.6336)	
asian	-3482.0310	
	(4323.7412)	
othrace	-5882.7376	
	(5903.7918)	
midwest	-5889.2289*	
	(3416.7197)	
south	-4642.4160	
	(3244.0177)	
west	-3858.1026	
	(3509.7962)	
lesshischool	-37.1351	
	(2969.2443)	
collegeplus	276.7460	
	(2570.1213)	
poor	709.9060	
	(3143.0255)	
nearpoor	-3908.1159	
	(5096.1782)	
middleinc	6912.1575*	

	(3827.9773)	
highinc	7555.6833*	
	(4192.9226)	
_cons	17466.0135***	
	(5442.5835)	
No. of Obs.	6003.0000	6003.0000
R-Squared		

	(1)	(2)
	inpatient	
	Coef./std.errors	Coef./std.errors
main		
overweight	0.0523	
	(0.1960)	
obese	0.0448	
	(0.1901)	
unins	0.0726	
	(0.2308)	
medicaid	0.6261***	
	(0.2323)	
medicare	1.5392***	
	(0.2890)	
female	0.5248***	
	(0.1648)	
smoker	0.2435	
	(0.2005)	
married	0.1666	
	(0.1642)	
black	-0.4966**	
	(0.2200)	
amind	-1.4320	
	(1.0599)	
asian	-0.3752	
	(0.3087)	
othrace	-0.1529	
	(0.4434)	
midwest	0.2615	
	(0.2898)	
south	0.5799**	
	(0.2601)	
west	0.2975	
	(0.2697)	
lesshischool	-0.2281	
	(0.2305)	
collegeplus	0.2896	
	(0.1873)	
poor	0.6695**	
	(0.2702)	
nearpoor	0.7161**	
	(0.3385)	
middleinc	0.3556	
	(0.2672)	
highinc	0.2496	
	(0.2901)	

_bs_1		7.5258e+08
		(2.6338e+09)
_bs_2		0.0243
		(0.0857)
_cons	-4.2975***	
	(0.4278)	
glm		
overweight	578.7015	
	(3890.3042)	
obese	-1989.1176	
	(3692.2765)	
unins	-1275.3493	
	(4573.8666)	
medicaid	-2468.7091	
	(4192.3252)	
medicare	10631.9424**	
	(4999.7143)	
female	-2207.3203	
	(3150.0022)	
smoker	216.6305	
	(3988.6425)	
married	-5147.8491	
	(3293.0477)	
black	-5535.2884	
	(4235.2011)	
amind	-10658.6154	
	(20481.2934)	
asian	-2678.8126	
	(6044.3735)	
othrace	-7442.5942	
	(8399.9883)	
midwest	1521.1412	
	(5545.9762)	
south	1449.9104	
	(5000.6107)	
west	3346.0409	
	(5234.3123)	
lesshischool	-1416.5722	
	(4435.0461)	
collegeplus	3045.3533	
	(3385.5266)	
poor	1112.5433	
	(5421.9261)	
nearpoor	-3361.2569	
	(6538.1343)	
middleinc	6532.3915	

	(5364.3070)	
highinc	2936.1370	
	(5691.7229)	
_cons	16511.2452**	
	(8357.8918)	
No. of Obs.	3609.0000	3609.0000
R-Squared		

	(1)	(2)
	inpatient	
	Coef./std.errors	Coef./std.errors
main		
overweight	0.0522	
	(0.2112)	
obese	0.3003	
	(0.1998)	
unins	-0.6464**	
	(0.2659)	
medicaid	0.3322	
	(0.2148)	
medicare	0.8786***	
	(0.2342)	
female	0.6101***	
	(0.1640)	
smoker	-0.0067	
	(0.1950)	
married	-0.2846*	
	(0.1689)	
black	-0.2158	
	(0.1941)	
amind	-0.1869	
	(1.0339)	
asian	-0.1634	
	(0.3457)	
othrace	0.4212	
	(0.4012)	
midwest	0.0462	
	(0.2385)	
south	0.0376	
	(0.2124)	
west	-0.5042**	
	(0.2497)	
lesshischool	0.0202	
	(0.2121)	
collegeplus	-0.1514	
	(0.1790)	
poor	0.0784	
	(0.2455)	
nearpoor	-0.1851	
	(0.3669)	
middleinc	-0.1369	
	(0.2330)	
highinc	-0.3621	
	(0.2546)	

_bs_1		3.0935e+09
		(4.1870e+09)
_bs_2		0.0755
		(0.0986)
_cons	-2.9520***	
	(0.3543)	
glm		
overweight	4868.5274	
	(6524.1937)	
obese	7743.7505	
	(6061.3125)	
unins	-11227.4818	
	(8299.8400)	
medicaid	-15236.3739**	
	(6404.5958)	
medicare	106.4320	
	(6659.1093)	
female	-5160.8221	
	(5039.5647)	
smoker	519.2615	
	(5905.8152)	
married	-9827.0313*	
	(5327.8644)	
black	4233.5092	
	(5943.8592)	
amind	-12994.2335	
	(32287.2769)	
asian	-4513.6362	
	(10794.1328)	
othrace	18859.8587	
	(11929.1323)	
midwest	-9075.0166	
	(7286.1500)	
south	-6648.4172	
	(6483.7964)	
west	2149.0765	
	(7882.4216)	
lesshischool	4070.8278	
	(6695.5717)	
collegeplus	11846.6911**	
	(5467.1531)	
poor	-137.6396	
	(7362.0981)	
nearpoor	-8036.6157	
	(11148.5031)	
middleinc	-3762.0850	

	(7169.3835)	
highinc	-2846.4830	
	(7936.5028)	
_cons	29805.3393***	
	(10415.3659)	
No. of Obs.	3652.0000	3652.0000
R-Squared		

	(1)	(2)
	inpatient	
	Coef./std.errors	Coef./std.errors
main		
overweight	-0.2625	
	(0.1801)	
obese	0.1002	
	(0.1689)	
unins	-0.2196	
	(0.2580)	
medicaid	0.6957***	
	(0.1807)	
medicare	1.0369***	
	(0.1621)	
female	-0.0047	
	(0.1388)	
smoker	0.2747*	
	(0.1644)	
married	-0.0899	
	(0.1508)	
black	0.1655	
	(0.1605)	
amind	-0.8512	
	(1.0344)	
asian	-0.1806	
	(0.3487)	
othrace	0.2422	
	(0.3797)	
midwest	0.1179	
	(0.2090)	
south	-0.0881	
	(0.1940)	
west	-0.1945	
	(0.2165)	
lesshischool	-0.0283	
	(0.1899)	
collegeplus	0.2870*	
	(0.1578)	
poor	0.1536	
	(0.2105)	
nearpoor	-0.2876	
	(0.3326)	
middleinc	-0.1616	
	(0.2152)	
highinc	-0.3351	
	(0.2324)	

_bs_1		-6.6452e+09
		(6.7000e+09)
_bs_2		-0.1016
		(0.0966)
_cons	-2.7485***	
	(0.3164)	
glm		
overweight	-1107.0723	
	(4717.6241)	
obese	-5744.6907	
	(4330.7822)	
unins	142.8105	
	(6895.8863)	
medicaid	-8121.8299*	
	(4530.6711)	
medicare	-6427.7576	
	(4045.4466)	
female	2298.1761	
	(3595.2751)	
smoker	-456.3687	
	(4380.6724)	
married	8154.2401*	
	(4185.1934)	
black	212.4645	
	(4022.3443)	
amind	-17440.4036	
	(27878.0165)	
asian	-18642.0964**	
	(9232.0893)	
othrace	-714.9842	
	(9618.1798)	
midwest	-5583.9283	
	(5439.0387)	
south	-6472.1873	
	(5030.5630)	
west	-427.9161	
	(5685.6240)	
lesshischool	3739.6139	
	(4940.1871)	
collegeplus	4451.8055	
	(4151.6124)	
poor	-142.5480	
	(5333.8136)	
nearpoor	-7427.6623	
	(8664.3927)	
middleinc	-27.1970	

	(5492.0452)	
highinc	-3606.8024	
	(6104.6425)	
_cons	30683.5370***	
	(8389.7270)	
No. of Obs.	3353.0000	3353.0000
R-Squared		

	(1)	(2)
	inpatient	
	Coef./std.errors	Coef./std.errors
main		
overweight	0.2030	
	(0.1703)	
obese	0.2306	
	(0.1714)	
female	0.0625	
	(0.1360)	
smoker	0.4135**	
	(0.1845)	
married	-0.3582**	
	(0.1437)	
black	-0.0297	
	(0.1748)	
amind	0.6083	
	(0.6573)	
asian	-0.2935	
	(0.3212)	
othrace	-0.3379	
	(0.4804)	
midwest	0.1274	
	(0.2186)	
south	0.1298	
	(0.1960)	
west	-0.3049	
	(0.2271)	
lesshischool	0.0608	
	(0.1939)	
collegeplus	0.2686*	
	(0.1628)	
poor	-0.2934	
	(0.2428)	
nearpoor	0.3434	
	(0.2887)	
middleinc	-0.2061	
	(0.2032)	
highinc	-0.3228	
	(0.2098)	
_bs_1		6.1996e+09
		(4.8753e+09)
_bs_2		0.1097
		(0.0812)
_cons	-2.0278***	
	(0.2964)	

glm		
overweight	2853.8682	
	(3552.3007)	
obese	887.3801	
	(3452.4311)	
female	-239.7047	
	(2770.3067)	
smoker	-4025.5523	
	(3752.5214)	
married	-467.9081	
	(2946.9202)	
black	-1519.1410	
	(3404.4166)	
amind	1985.6599	
	(12734.4758)	
asian	5357.7238	
	(6600.0997)	
othrace	7049.1023	
	(9924.3713)	
midwest	-546.6661	
	(4507.5557)	
south	77.5321	
	(4019.0285)	
west	2705.7450	
	(4835.2789)	
lesshischool	-1198.5243	
	(3907.8267)	
collegeplus	-6176.0909*	
	(3293.5594)	
poor	5528.8371	
	(4904.4178)	
nearpoor	2292.1699	
	(5560.5696)	
middleinc	8258.2232**	
	(4113.3533)	
highinc	5861.1050	
	(4303.4706)	
_cons	16411.2265***	
	(5865.3895)	
No. of Obs.	2309.0000	2309.0000
R-Squared		

	(1)	(2)
	inpatient	
	Coef./std.errors	Coef./std.errors
main		
overweight	0.1718	
	(0.1627)	
obese	0.4618***	
	(0.1758)	
female	-0.0996	
	(0.1479)	
smoker	-0.2770	
	(0.4153)	
married	-0.2069	
	(0.1534)	
black	-0.1052	
	(0.1999)	
amind	0.6259	
	(0.6935)	
asian	-1.3503***	
	(0.4735)	
othrace	-0.3491	
	(0.7619)	
midwest	-0.0528	
	(0.2170)	
south	0.0084	
	(0.1889)	
west	0.1205	
	(0.2111)	
lesshischool	0.0578	
	(0.1770)	
collegeplus	-0.3439**	
	(0.1672)	
poor	0.0323	
	(0.2272)	
nearpoor	0.2964	
	(0.3009)	
middleinc	0.2612	
	(0.2010)	
highinc	0.1550	
	(0.2168)	
_bs_1		3.2970e+09
		(3.4988e+09)
_bs_2		0.0684
		(0.0703)
_cons	-1.6675***	
	(0.2752)	

glm		
overweight	1000.0608	
	(3052.5234)	
obese	3556.7138	
	(3301.1689)	
female	-3886.7867	
	(2791.7741)	
smoker	-3819.6966	
	(8029.9174)	
married	-1059.6734	
	(3002.4022)	
black	-1180.2407	
	(3735.1474)	
amind	-15863.6516	
	(12125.8145)	
asian	-8807.9483	
	(9423.6021)	
othrace	-6890.7714	
	(14774.8247)	
midwest	-1398.6981	
	(4046.3570)	
south	-2155.0455	
	(3549.7179)	
west	5405.6448	
	(3938.2869)	
lesshischool	5673.2746*	
	(3302.2334)	
collegeplus	3974.9115	
	(3163.1743)	
poor	-2483.8784	
	(4274.1909)	
nearpoor	-2065.3277	
	(5561.7259)	
middleinc	-3304.0186	
	(3922.6660)	
highinc	-2042.2455	
	(4050.4232)	
_cons	17602.1801***	
	(5577.9757)	
No. of Obs.	1640.0000	1640.0000
R-Squared		

	(1)	(2)
	inpatient	
	Coef./std.errors	Coef./std.errors
main		
overweight	0.0464	
	(0.0718)	
obese	0.2066***	
	(0.0708)	
age3544	-0.1680	
	(0.1030)	
age4554	-0.1363	
	(0.1001)	
age5564	0.2197**	
	(0.0919)	
age6574	0.6304***	
	(0.0901)	
age75plus	0.9587***	
	(0.0921)	
female	0.4871***	
	(0.0604)	
smoker	0.2139***	
	(0.0802)	
married	0.0390	
	(0.0625)	
black	-0.0356	
	(0.0734)	
amind	0.0187	
	(0.3414)	
asian	-0.4037***	
	(0.1320)	
othrace	-0.0558	
	(0.1753)	
midwest	0.1424	
	(0.0904)	
south	0.0507	
	(0.0818)	
west	-0.1719*	
	(0.0915)	
lesshischool	-0.0384	
	(0.0806)	
collegeplus	0.0159	
	(0.0670)	
poor	0.4383***	
	(0.0913)	
nearpoor	0.1568	
	(0.1335)	

middleinc	-0.2145**	
	(0.0891)	
highinc	-0.4137***	
	(0.0934)	
_bs_1		9.5831e+09
		(1.0292e+10)
_bs_2		0.0352
		(0.0373)
_cons	-2.9328***	
	(0.1321)	
glm		
overweight	1288.5776	
	(1600.6419)	
obese	176.0602	
	(1557.4357)	
age3544	3567.7275	
	(2335.3043)	
age4554	9775.5514***	
	(2254.9131)	
age5564	11178.4394***	
	(2077.5825)	
age6574	5315.5822***	
	(2050.5221)	
age75plus	2940.4923	
	(2077.9635)	
female	-2078.6191	
	(1395.0294)	
smoker	-768.7965	
	(1811.2373)	
married	-822.3417	
	(1417.6979)	
black	74.3313	
	(1602.6946)	
amind	-3053.4765	
	(7447.2250)	
asian	-4012.1297	
	(3002.9835)	
othrace	3178.4434	
	(3900.4726)	
midwest	-4655.1744**	
	(1990.8601)	
south	-3191.5948*	
	(1809.1519)	
west	532.5519	
	(2034.2688)	
lesshischool	2640.4250	

	(1764.2357)	
collegeplus	2523.8087*	
	(1487.1015)	
poor	-1323.0905	
	(2016.9226)	
nearpoor	-3542.9993	
	(2919.8651)	
middleinc	3048.0068	
	(1986.3387)	
highinc	2729.1593	
	(2090.4483)	
_cons	14146.5026***	
	(3053.8433)	
No. of Obs.	17196.0000	17196.0000
R-Squared		

	(1)	(2)
	inpatient	
	Coef./std.errors	Coef./std.errors
main		
overweight	0.0205	
	(0.1052)	
obese	0.3035***	
	(0.1043)	
age3544	-0.1575	
	(0.1399)	
age4554	-0.1674	
	(0.1381)	
age5564	0.0910	
	(0.1335)	
age6574	0.6653***	
	(0.1443)	
age75plus	1.3147***	
	(0.1432)	
female	0.5888***	
	(0.0873)	
smoker	0.0279	
	(0.1434)	
married	0.3459***	
	(0.0918)	
black	-0.0590	
	(0.1202)	
amind	0.4310	
	(0.5286)	
asian	-0.0856	
	(0.1605)	
othrace	0.0156	
	(0.2571)	
midwest	-0.0353	
	(0.1327)	
south	-0.0236	
	(0.1228)	
west	-0.2072	
	(0.1361)	
lesshischool	-0.0083	
	(0.1520)	
collegeplus	0.0666	
	(0.0969)	
poor	0.1930	
	(0.1944)	
nearpoor	0.0858	
	(0.2749)	

middleinc	-0.1220	
	(0.1364)	
highinc	-0.2741**	
	(0.1373)	
_bs_1		2.8957e+09
		(8.1595e+09)
_bs_2		0.0170
		(0.0476)
_cons	-3.4249***	
	(0.2070)	
glm		
overweight	752.3977	
	(2515.2907)	
obese	32.0338	
	(2499.7390)	
age3544	4765.7158	
	(3369.0131)	
age4554	15240.7460***	
	(3305.8907)	
age5564	18602.8074***	
	(3231.4261)	
age6574	5434.0670	
	(3482.4903)	
age75plus	3144.7592	
	(3425.9951)	
female	-3276.5865	
	(2160.5240)	
smoker	2526.2321	
	(3466.1274)	
married	-2219.4602	
	(2244.6451)	
black	-2170.8368	
	(2879.5370)	
amind	-7950.3296	
	(12428.9529)	
asian	-4808.7685	
	(3880.9894)	
othrace	1219.0099	
	(6230.6698)	
midwest	-5566.5183*	
	(3155.1495)	
south	-5677.3015*	
	(2929.0091)	
west	476.2371	
	(3236.2827)	
lesshischool	5763.7376	

	(3588.4235)	
collegeplus	5294.5318**	
	(2326.1911)	
poor	678.8772	
	(4581.5360)	
nearpoor	-4220.5826	
	(6468.7403)	
middleinc	391.8166	
	(3257.5235)	
highinc	-1241.9206	
	(3285.3796)	
_cons	17485.2045***	
	(5339.0021)	
No. of Obs.	11222.0000	11222.0000
R-Squared		

	(1)	(2)
	inpatient	
	Coef./std.errors	Coef./std.errors
main		
overweight	-0.0184	
	(0.1256)	
obese	-0.0335	
	(0.1210)	
age3544	-0.2660	
	(0.1640)	
age4554	-0.1371	
	(0.1624)	
age5564	0.3728***	
	(0.1433)	
age6574	0.6648***	
	(0.1688)	
age75plus	0.7066***	
	(0.1799)	
female	0.6100***	
	(0.1141)	
smoker	0.0871	
	(0.1185)	
married	-0.0743	
	(0.1234)	
black	-0.1222	
	(0.1132)	
amind	-1.4525	
	(1.0248)	
asian	-0.8137***	
	(0.2679)	
othrace	-0.1693	
	(0.2795)	
midwest	0.3883**	
	(0.1508)	
south	0.3190**	
	(0.1355)	
west	-0.3399**	
	(0.1473)	
lesshischool	-0.0845	
	(0.1171)	
collegeplus	0.1001	
	(0.1271)	
poor	0.2252*	
	(0.1299)	
nearpoor	-0.0924	
	(0.1893)	

middleinc	-0.1271	
	(0.1722)	
highinc	-0.3030	
	(0.3063)	
_bs_1		4.4569e+09
		(4.0958e+09)
_bs_2		0.0847
		(0.0763)
_cons	-2.4546***	
	(0.2029)	
glm		
overweight	4630.8584	
	(2824.0484)	
obese	5.1181	
	(2712.1932)	
age3544	2915.6094	
	(3807.0962)	
age4554	2385.4156	
	(3727.2299)	
age5564	4938.5405	
	(3301.8311)	
age6574	5023.4514	
	(3891.6128)	
age75plus	3814.0517	
	(4037.7097)	
female	-1198.7510	
	(2705.7152)	
smoker	1480.4665	
	(2724.1426)	
married	-2180.0988	
	(2758.9566)	
black	-205.8441	
	(2511.8396)	
amind	-4927.1294	
	(24398.4671)	
asian	-2474.9894	
	(6218.5735)	
othrace	-495.9784	
	(6275.8189)	
midwest	-5631.2814*	
	(3390.2672)	
south	-1853.6195	
	(3018.7275)	
west	-1711.0233	
	(3365.4281)	
lesshischool	883.7690	

	(2631.1152)	
collegeplus	-876.3627	
	(2820.7116)	
poor	-1334.0044	
	(2910.3403)	
nearpoor	-3354.3068	
	(4249.2817)	
middleinc	8086.3213**	
	(3960.1160)	
highinc	13141.6291*	
	(7013.4594)	
_cons	13817.9197***	
	(4758.5488)	
No. of Obs.	3772.0000	3772.0000
R-Squared		

	(1)	(2)
	inpatient	
	Coef./std.errors	Coef./std.errors
main		
overweight	0.1154	
	(0.1072)	
obese	0.1906*	
	(0.1097)	
age3544	0.5315	
	(0.4299)	
age4554	0.1224	
	(0.3980)	
age5564	0.4034	
	(0.3673)	
age6574	-0.0857	
	(0.3556)	
age75plus	0.2430	
	(0.3569)	
female	0.0748	
	(0.0888)	
smoker	0.3317***	
	(0.1267)	
married	-0.2437***	
	(0.0945)	
black	-0.0542	
	(0.1125)	
amind	-0.0417	
	(0.4556)	
asian	-0.4978**	
	(0.2399)	
othrace	-0.1877	
	(0.2972)	
midwest	0.0716	
	(0.1361)	
south	0.0515	
	(0.1208)	
west	-0.1478	
	(0.1409)	
lesshischool	0.1113	
	(0.1149)	
collegeplus	0.0763	
	(0.1019)	
poor	-0.0352	
	(0.1340)	
nearpoor	0.0589	
	(0.1841)	

middleinc	-0.0351	
	(0.1262)	
highinc	-0.1779	
	(0.1371)	
_bs_1		1.1613e+10*
		(6.2590e+09)
_bs_2		0.0907*
		(0.0478)
_cons	-1.9117***	
	(0.3852)	
glm		
overweight	3223.6035	
	(2225.1562)	
obese	1665.7601	
	(2237.0545)	
age3544	-5800.7395	
	(8698.5269)	
age4554	-7530.8476	
	(8171.1207)	
age5564	-12364.0650	
	(7577.4995)	
age6574	-12639.1432*	
	(7437.9768)	
age75plus	-14970.0130**	
	(7489.7610)	
female	-880.3699	
	(1839.3067)	
smoker	-511.3810	
	(2623.5606)	
married	-1299.9772	
	(1978.3760)	
black	657.6487	
	(2271.9367)	
amind	-5626.6795	
	(9289.1570)	
asian	671.9345	
	(5131.0400)	
othrace	5114.3179	
	(6186.2085)	
midwest	-379.5060	
	(2797.6047)	
south	1917.2964	
	(2472.6081)	
west	5601.5573*	
	(2935.3715)	
lesshischool	3127.9792	

	(2353.7386)	
collegeplus	979.6703	
	(2099.9814)	
poor	609.7110	
	(2725.0952)	
nearpoor	-2125.6033	
	(3722.2059)	
middleinc	3187.9857	
	(2614.5068)	
highinc	2323.4910	
	(2823.3175)	
_cons	26154.6249***	
	(8178.8433)	
No. of Obs.	4547.0000	4547.0000
R-Squared		

	(1)	(2)
	totalexp	
	Coef./std.errors	Coef./std.errors
main		
overweight	0.0956	
	(0.1176)	
obese	0.2103*	
	(0.1273)	
unins	-0.4908***	
	(0.1411)	
medicaid	0.4073**	
	(0.1658)	
medicare	1.2989**	
	(0.5255)	
female	0.6918***	
	(0.1022)	
smoker	0.3715**	
	(0.1531)	
married	0.5643***	
	(0.1183)	
black	-0.3274**	
	(0.1547)	
amind	-0.4447	
	(0.5644)	
asian	-0.3703*	
	(0.1961)	
othrace	-0.1723	
	(0.2584)	
midwest	-0.1059	
	(0.1653)	
south	-0.2135	
	(0.1522)	
west	-0.1463	
	(0.1577)	
lesshischool	-0.4055**	
	(0.1612)	
collegeplus	0.0731	
	(0.1275)	
poor	0.3173	
	(0.2029)	
nearpoor	-0.3457	
	(0.2722)	
middleinc	-0.1059	
	(0.1738)	
highinc	-0.0519	
	(0.1777)	

_bs_1		8.3590e+09
		(5.1241e+09)
_bs_2		0.0613
		(0.0375)
_cons	7.1212***	
	(0.2195)	
No. of Obs.	6003.0000	6003.0000
R-Squared		

	(1)	(2)
	totalexp	
	Coef./std.errors	Coef./std.errors
main		
overweight	-0.1197	
	(0.1336)	
obese	0.3724***	
	(0.1358)	
unins	-0.6085***	
	(0.1544)	
medicaid	0.0658	
	(0.1901)	
medicare	1.8221***	
	(0.3786)	
female	0.5338***	
	(0.1059)	
smoker	0.2534	
	(0.1559)	
married	-0.2070*	
	(0.1199)	
black	-0.5280***	
	(0.1648)	
amind	0.4618	
	(0.5239)	
asian	-0.4526**	
	(0.2070)	
othrace	-0.0551	
	(0.3230)	
midwest	0.1096	
	(0.1781)	
south	0.3274**	
	(0.1587)	
west	0.2036	
	(0.1648)	
lesshischool	-0.4674**	
	(0.1920)	
collegeplus	-0.0951	
	(0.1345)	
poor	0.5171**	
	(0.2369)	
nearpoor	0.5483*	
	(0.3104)	
middleinc	0.3065*	
	(0.1817)	
highinc	0.5288***	
	(0.1799)	

_bs_1		1.7153e+10***
		(6.3084e+09)
_bs_2		0.1468***
		(0.0518)
_cons	7.3118***	
	(0.2638)	
No. of Obs.	3609.0000	3609.0000
R-Squared		

	(1)	(2)
	totalexp	
	Coef./std.errors	Coef./std.errors
main		
overweight	0.1763	
	(0.1391)	
obese	0.3852***	
	(0.1378)	
unins	-0.8727***	
	(0.1836)	
medicaid	-0.1129	
	(0.2237)	
medicare	1.2227***	
	(0.2721)	
female	0.3174***	
	(0.1095)	
smoker	0.0685	
	(0.1514)	
married	-0.1791	
	(0.1275)	
black	-0.1345	
	(0.1731)	
amind	0.4063	
	(0.5916)	
asian	-0.2070	
	(0.2241)	
othrace	0.5706	
	(0.3522)	
midwest	-0.0939	
	(0.1781)	
south	-0.0334	
	(0.1618)	
west	-0.2616	
	(0.1731)	
lesshischool	-0.1003	
	(0.1937)	
collegeplus	0.3819***	
	(0.1268)	
poor	0.0263	
	(0.2537)	
nearpoor	0.0448	
	(0.3662)	
middleinc	-0.4198**	
	(0.2053)	
highinc	-0.3395*	
	(0.2034)	

_bs_1		2.4668e+10***
		(8.7772e+09)
_bs_2		0.1460***
		(0.0509)
_cons	8.2673***	
	(0.2876)	
No. of Obs.	3652.0000	3652.0000
R-Squared		

	(1)	(2)
	totalexp	
	Coef./std.errors	Coef./std.errors
main		
overweight	-0.1478	
	(0.1044)	
obese	0.1950*	
	(0.1060)	
unins	-0.0645	
	(0.1452)	
medicaid	0.5003***	
	(0.1590)	
medicare	0.7979***	
	(0.1511)	
female	0.0764	
	(0.0841)	
smoker	-0.0492	
	(0.1158)	
married	0.1435	
	(0.0944)	
black	0.0114	
	(0.1288)	
amind	-0.6729*	
	(0.3999)	
asian	-0.6642***	
	(0.2054)	
othrace	0.0039	
	(0.2746)	
midwest	0.0963	
	(0.1326)	
south	0.0918	
	(0.1207)	
west	0.0751	
	(0.1329)	
lesshischool	-0.1905	
	(0.1450)	
collegeplus	0.2212**	
	(0.0935)	
poor	0.1919	
	(0.1880)	
nearpoor	-0.2414	
	(0.2600)	
middleinc	0.0712	
	(0.1583)	
highinc	0.0298	
	(0.1536)	

_bs_1		1.8232e+10
		(1.1686e+10)
_bs_2		0.0763
		(0.0487)
_cons	8.2674***	
	(0.2118)	
No. of Obs.	3353.0000	3353.0000
R-Squared		

	(1)	(2)
	totalexp	
	Coef./std.errors	Coef./std.errors
main		
overweight	0.0778	
	(0.1040)	
obese	0.2716***	
	(0.1039)	
female	0.0163	
	(0.0854)	
smoker	-0.1473	
	(0.1314)	
married	-0.3581***	
	(0.0933)	
black	-0.1546	
	(0.1484)	
amind	0.3243	
	(0.5368)	
asian	-0.1820	
	(0.2075)	
othrace	-0.0190	
	(0.2746)	
midwest	0.2158	
	(0.1356)	
south	0.1699	
	(0.1230)	
west	-0.0560	
	(0.1331)	
lesshischool	-0.0552	
	(0.1387)	
collegeplus	-0.0357	
	(0.0994)	
poor	0.1475	
	(0.1911)	
nearpoor	0.1831	
	(0.2390)	
middleinc	0.0770	
	(0.1401)	
highinc	0.2532*	
	(0.1391)	
_bs_1		2.2574e+10**
		(1.0892e+10)
_bs_2		0.0961**
		(0.0450)
_cons	8.9751***	
	(0.1957)	

No. of Obs.	2309.0000	2309.0000
R-Squared		

	(1)	(2)
	totalexp	
	Coef./std.errors	Coef./std.errors
main		
overweight	0.0166	
	(0.0866)	
obese	0.2884***	
	(0.1023)	
female	-0.0630	
	(0.0826)	
smoker	-0.4090*	
	(0.2209)	
married	0.0170	
	(0.0874)	
black	-0.2261	
	(0.1484)	
amind	0.1736	
	(0.4574)	
asian	-0.6627***	
	(0.2030)	
othrace	-0.6672	
	(0.4626)	
midwest	-0.0340	
	(0.1154)	
south	-0.0291	
	(0.1040)	
west	0.2125*	
	(0.1210)	
lesshischool	0.0003	
	(0.1139)	
collegeplus	-0.1261	
	(0.0879)	
poor	0.0300	
	(0.1455)	
nearpoor	-0.0920	
	(0.1858)	
middleinc	-0.1334	
	(0.1139)	
highinc	-0.0499	
	(0.1130)	
_bs_1		1.2109e+10**
		(4.7852e+09)
_bs_2		0.0696**
		(0.0277)
_cons	9.2590***	
	(0.1568)	

No. of Obs.	1640.0000	1640.0000
R-Squared		

	(1)	(2)
	totalexp	
	Coef./std.errors	Coef./std.errors
main		
overweight	0.0518	
	(0.0522)	
obese	0.2605***	
	(0.0543)	
age3544	0.3983***	
	(0.0696)	
age4554	0.7507***	
	(0.0678)	
age5564	1.0503***	
	(0.0674)	
age6574	1.3806***	
	(0.0716)	
age75plus	1.4767***	
	(0.0821)	
female	0.3347***	
	(0.0435)	
smoker	0.1120*	
	(0.0664)	
married	0.0203	
	(0.0471)	
black	-0.2499***	
	(0.0698)	
amind	0.0549	
	(0.2690)	
asian	-0.4700***	
	(0.0917)	
othrace	-0.0115	
	(0.1350)	
midwest	0.0142	
	(0.0690)	
south	-0.0073	
	(0.0625)	
west	-0.0265	
	(0.0673)	
lesshischool	-0.0838	
	(0.0756)	
collegeplus	0.1145**	
	(0.0507)	
poor	0.4044***	
	(0.0967)	
nearpoor	0.0119	
	(0.1320)	

middleinc	-0.0903	
	(0.0769)	
highinc	-0.1049	
	(0.0750)	
_bs_1		8.6358e+10***
		(1.8597e+10)
_bs_2		0.0845***
		(0.0182)
_cons	7.4595***	
	(0.1027)	
No. of Obs.	17196.0000	17196.0000
R-Squared		

	(1)	(2)
	totalexp	
	Coef./std.errors	Coef./std.errors
main		
overweight	0.0328	
	(0.0652)	
obese	0.2645***	
	(0.0686)	
age3544	0.3466***	
	(0.0826)	
age4554	0.7116***	
	(0.0807)	
age5564	1.0338***	
	(0.0815)	
age6574	1.3575***	
	(0.1016)	
age75plus	1.6255***	
	(0.1231)	
female	0.4312***	
	(0.0548)	
smoker	0.1853**	
	(0.0902)	
married	0.1397**	
	(0.0584)	
black	-0.2332**	
	(0.0933)	
amind	0.2200	
	(0.3944)	
asian	-0.4313***	
	(0.1115)	
othrace	0.0996	
	(0.1714)	
midwest	-0.0217	
	(0.0863)	
south	-0.0140	
	(0.0788)	
west	-0.0343	
	(0.0857)	
lesshischool	-0.0597	
	(0.1132)	
collegeplus	0.2129***	
	(0.0634)	
poor	0.2228	
	(0.1738)	
nearpoor	0.0693	
	(0.2328)	

middleinc	-0.0613	
	(0.1097)	
highinc	-0.0854	
	(0.1068)	
_bs_1		5.4389e+10***
		(1.3762e+10)
_bs_2		0.0829***
		(0.0208)
_cons	7.2319***	
	(0.1433)	
No. of Obs.	11222.0000	11222.0000
R-Squared		

	(1)	(2)
	totalexp	
	Coef./std.errors	Coef./std.errors
main		
overweight	0.1459	
	(0.1223)	
obese	0.2648**	
	(0.1196)	
age3544	0.3796**	
	(0.1476)	
age4554	0.7129***	
	(0.1486)	
age5564	1.1989***	
	(0.1469)	
age6574	1.6442***	
	(0.1931)	
age75plus	1.4095***	
	(0.1948)	
female	0.0864	
	(0.1034)	
smoker	-0.0103	
	(0.1149)	
married	-0.2014*	
	(0.1156)	
black	-0.4125***	
	(0.1255)	
amind	-0.6125	
	(0.5036)	
asian	-0.5264**	
	(0.2065)	
othrace	-0.0388	
	(0.2459)	
midwest	0.0388	
	(0.1485)	
south	0.0692	
	(0.1425)	
west	-0.3435**	
	(0.1376)	
lesshischool	-0.1840	
	(0.1211)	
collegeplus	-0.1012	
	(0.1207)	
poor	0.3887***	
	(0.1305)	
nearpoor	-0.1142	
	(0.1788)	

middleinc	0.0508	
	(0.1515)	
highinc	0.2635	
	(0.2143)	
_bs_1		1.6099e+10**
		(7.8086e+09)
_bs_2		0.0940**
		(0.0451)
_cons	7.9736***	
	(0.1739)	
No. of Obs.	3772.0000	3772.0000
R-Squared		

	(1)	(2)
	totalexp	
	Coef./std.errors	Coef./std.errors
main		
overweight	0.0525	
	(0.0649)	
obese	0.2427***	
	(0.0672)	
age3544	0.7426**	
	(0.3408)	
age4554	0.4260	
	(0.2927)	
age5564	0.3708	
	(0.2706)	
age6574	-0.0144	
	(0.2578)	
age75plus	0.0292	
	(0.2605)	
female	0.0193	
	(0.0550)	
smoker	-0.0896	
	(0.0920)	
married	-0.1957***	
	(0.0594)	
black	-0.1140	
	(0.0940)	
amind	0.0023	
	(0.2793)	
asian	-0.3765***	
	(0.1431)	
othrace	-0.1767	
	(0.1891)	
midwest	0.0645	
	(0.0840)	
south	0.0586	
	(0.0758)	
west	0.0274	
	(0.0851)	
lesshischool	-0.0003	
	(0.0829)	
collegeplus	-0.0374	
	(0.0619)	
poor	0.0916	
	(0.1034)	
nearpoor	-0.0596	
	(0.1333)	

middleinc	-0.0006	
	(0.0835)	
highinc	0.1151	
	(0.0833)	
_bs_1		4.0373e+10***
		(1.2675e+10)
_bs_2		0.0824***
		(0.0254)
_cons	9.0896***	
	(0.2845)	
No. of Obs.	4547.0000	4547.0000
R-Squared		

	(1)	(2)
	outpatient	
	Coef./std.errors	Coef./std.errors
main		
overweight	0.2732**	
	(0.1136)	
obese	0.3974***	
	(0.1189)	
unins	-0.9566***	
	(0.1349)	
medicaid	0.0001	
	(0.1566)	
medicare	0.9734*	
	(0.5011)	
female	0.8228***	
	(0.0972)	
smoker	0.2134	
	(0.1394)	
married	0.2668**	
	(0.1102)	
black	-0.6211***	
	(0.1409)	
amind	-0.7030	
	(0.5390)	
asian	-0.0436	
	(0.1950)	
othrace	-0.0841	
	(0.2513)	
midwest	-0.1462	
	(0.1556)	
south	-0.3264**	
	(0.1414)	
west	0.0971	
	(0.1498)	
lesshischool	-0.2327	
	(0.1586)	
collegeplus	0.3211***	
	(0.1145)	
poor	-0.0273	
	(0.1922)	
nearpoor	-0.2732	
	(0.2569)	
middleinc	-0.3138*	
	(0.1632)	
highinc	-0.0604	
	(0.1680)	

_bs_1		5.2670e+09***
		(1.5830e+09)
_bs_2		0.1003***
		(0.0294)
_cons	6.1753***	
	(0.1990)	
No. of Obs.	6003.0000	6003.0000
R-Squared		

	(1)	(2)
	outpatient	
	Coef./std.errors	Coef./std.errors
main		
overweight	0.0779	
	(0.1326)	
obese	0.4582***	
	(0.1314)	
unins	-0.5075***	
	(0.1613)	
medicaid	0.0108	
	(0.2027)	
medicare	1.6019***	
	(0.3711)	
female	0.7064***	
	(0.1024)	
smoker	-0.2132	
	(0.1454)	
married	-0.2257**	
	(0.1147)	
black	-0.4649***	
	(0.1649)	
amind	0.2103	
	(0.5218)	
asian	-0.3517*	
	(0.2039)	
othrace	-0.2089	
	(0.3165)	
midwest	0.1593	
	(0.1711)	
south	0.0458	
	(0.1522)	
west	0.1935	
	(0.1630)	
lesshischool	-0.4745***	
	(0.1841)	
collegeplus	0.0779	
	(0.1331)	
poor	-0.0432	
	(0.2255)	
nearpoor	0.0040	
	(0.3131)	
middleinc	0.2751	
	(0.1878)	
highinc	0.6407***	
	(0.1836)	

_bs_1		7.0436e+09***
		(2.2538e+09)
_bs_2		0.1610***
		(0.0484)
_cons	6.2584***	
	(0.2698)	
No. of Obs.	3609.0000	3609.0000
R-Squared		

	(1)	(2)
	outpatient	
	Coef./std.errors	Coef./std.errors
main		
overweight	0.1240	
	(0.1673)	
obese	0.3253*	
	(0.1664)	
unins	-0.9530***	
	(0.2152)	
medicaid	-0.2383	
	(0.2613)	
medicare	1.2667***	
	(0.3387)	
female	0.4565***	
	(0.1340)	
smoker	-0.2534	
	(0.1827)	
married	-0.0520	
	(0.1542)	
black	-0.0828	
	(0.2085)	
amind	0.5862	
	(0.7113)	
asian	-0.3696	
	(0.2742)	
othrace	0.3207	
	(0.4313)	
midwest	-0.2544	
	(0.2192)	
south	-0.1071	
	(0.1939)	
west	-0.1650	
	(0.2084)	
lesshischool	-0.4512*	
	(0.2412)	
collegeplus	0.4859***	
	(0.1538)	
poor	-0.1215	
	(0.3087)	
nearpoor	-0.4420	
	(0.4456)	
middleinc	-0.2887	
	(0.2507)	
highinc	-0.2476	
	(0.2451)	

_bs_1		7.9785e+09**
		(3.6677e+09)
_bs_2		0.1204**
		(0.0560)
_cons	7.2417***	
	(0.3458)	
No. of Obs.	3652.0000	3652.0000
R-Squared		

	(1)	(2)
	outpatient	
	Coef./std.errors	Coef./std.errors
main		
overweight	0.0814	
	(0.1102)	
obese	0.3615***	
	(0.1125)	
unins	-0.1476	
	(0.1516)	
medicaid	0.1723	
	(0.1612)	
medicare	0.6900***	
	(0.1558)	
female	0.2237**	
	(0.0889)	
smoker	-0.0401	
	(0.1196)	
married	0.2396**	
	(0.0955)	
black	-0.0735	
	(0.1367)	
amind	-0.2792	
	(0.4199)	
asian	-0.4676**	
	(0.2144)	
othrace	-0.1725	
	(0.2849)	
midwest	0.0863	
	(0.1380)	
south	-0.0453	
	(0.1256)	
west	0.0950	
	(0.1380)	
lesshischool	-0.1523	
	(0.1519)	
collegeplus	0.3159***	
	(0.0973)	
poor	0.3571*	
	(0.1931)	
nearpoor	0.0472	
	(0.2723)	
middleinc	0.2758	
	(0.1680)	
highinc	0.5662***	
	(0.1639)	

_bs_1		1.1276e+10***
		(3.7570e+09)
_bs_2		0.1287***
		(0.0403)
_cons	6.6694***	
	(0.2215)	
No. of Obs.	3353.0000	3353.0000
R-Squared		

	(1)	(2)
	outpatient	
	Coef./std.errors	Coef./std.errors
main		
overweight	-0.0397	
	(0.1078)	
obese	0.0096	
	(0.1072)	
female	-0.1143	
	(0.0864)	
smoker	-0.1523	
	(0.1377)	
married	-0.2737***	
	(0.0942)	
black	-0.2027	
	(0.1587)	
amind	-0.2387	
	(0.5527)	
asian	-0.1711	
	(0.2175)	
othrace	-0.4024	
	(0.2864)	
midwest	0.1467	
	(0.1434)	
south	0.0346	
	(0.1297)	
west	0.0584	
	(0.1414)	
lesshischool	-0.2208	
	(0.1422)	
collegeplus	0.1014	
	(0.1017)	
poor	0.0825	
	(0.1972)	
nearpoor	0.1197	
	(0.2458)	
middleinc	0.2112	
	(0.1469)	
highinc	0.4618***	
	(0.1417)	
_bs_1		2.5581e+08
		(3.2787e+09)
_bs_2		0.0033
		(0.0427)
_cons	7.8640***	
	(0.2055)	

No. of Obs.	2309.0000	2309.0000
R-Squared		

	(1)	(2)
	outpatient	
	Coef./std.errors	Coef./std.errors
main		
overweight	0.1120	
	(0.1098)	
obese	0.0114	
	(0.1299)	
female	-0.1326	
	(0.1066)	
smoker	-0.4328	
	(0.2793)	
married	0.0125	
	(0.1114)	
black	-0.3315*	
	(0.1920)	
amind	-0.1824	
	(0.5791)	
asian	-0.8516***	
	(0.2539)	
othrace	-0.3824	
	(0.5896)	
midwest	-0.0316	
	(0.1447)	
south	-0.0780	
	(0.1314)	
west	0.0907	
	(0.1516)	
lesshischool	-0.4838***	
	(0.1445)	
collegeplus	-0.0459	
	(0.1109)	
poor	-0.0026	
	(0.1881)	
nearpoor	-0.3634	
	(0.2343)	
middleinc	-0.0368	
	(0.1461)	
highinc	-0.0242	
	(0.1479)	
_bs_1		1.3374e+08
		(1.5634e+09)
_bs_2		0.0024
		(0.0281)
_cons	8.2800***	
	(0.1885)	

No. of Obs.	1640.0000	1640.0000
R-Squared		

	(1)	(2)
	outpatient	
	Coef./std.errors	Coef./std.errors
main		
overweight	0.1353**	
	(0.0592)	
obese	0.2906***	
	(0.0616)	
age3544	0.3122***	
	(0.0789)	
age4554	0.6911***	
	(0.0766)	
age5564	0.9345***	
	(0.0768)	
age6574	1.1596***	
	(0.0810)	
age75plus	1.2920***	
	(0.0933)	
female	0.3503***	
	(0.0495)	
smoker	-0.0543	
	(0.0741)	
married	0.0335	
	(0.0529)	
black	-0.2975***	
	(0.0792)	
amind	-0.1784	
	(0.3048)	
asian	-0.3262***	
	(0.1051)	
othrace	-0.1928	
	(0.1524)	
midwest	0.0471	
	(0.0783)	
south	-0.0669	
	(0.0703)	
west	0.1164	
	(0.0765)	
lesshischool	-0.2284***	
	(0.0862)	
collegeplus	0.2238***	
	(0.0573)	
poor	0.1383	
	(0.1098)	
nearpoor	-0.0767	
	(0.1494)	

middleinc	0.0172	
	(0.0870)	
highinc	0.1623*	
	(0.0849)	
_bs_1		3.3030e+10***
		(7.2952e+09)
_bs_2		0.0899***
		(0.0196)
_cons	6.3308***	
	(0.1194)	
No. of Obs.	17196.0000	17196.0000
R-Squared		

	(1)	(2)
	outpatient	
	Coef./std.errors	Coef./std.errors
main		
overweight	0.0914	
	(0.0702)	
obese	0.2788***	
	(0.0743)	
age3544	0.2914***	
	(0.0890)	
age4554	0.6493***	
	(0.0871)	
age5564	0.9161***	
	(0.0886)	
age6574	1.1598***	
	(0.1097)	
age75plus	1.4980***	
	(0.1332)	
female	0.4508***	
	(0.0590)	
smoker	-0.0250	
	(0.0949)	
married	0.1340**	
	(0.0632)	
black	-0.1998**	
	(0.1005)	
amind	-0.0004	
	(0.4249)	
asian	-0.4189***	
	(0.1199)	
othrace	-0.0554	
	(0.1835)	
midwest	0.0063	
	(0.0931)	
south	-0.1456*	
	(0.0845)	
west	0.0890	
	(0.0924)	
lesshischool	-0.3231***	
	(0.1217)	
collegeplus	0.3017***	
	(0.0682)	
poor	0.0794	
	(0.1856)	
nearpoor	-0.0864	
	(0.2517)	

middleinc	0.0703	
	(0.1182)	
highinc	0.1840	
	(0.1152)	
_bs_1		2.1914e+10***
		(5.6710e+09)
_bs_2		0.0840***
		(0.0216)
_cons	6.1695***	
	(0.1542)	
No. of Obs.	11222.0000	11222.0000
R-Squared		

	(1)	(2)
	outpatient	
	Coef./std.errors	Coef./std.errors
main		
overweight	0.4025***	
	(0.1269)	
obese	0.5201***	
	(0.1214)	
age3544	0.2521*	
	(0.1517)	
age4554	0.6434***	
	(0.1531)	
age5564	0.9584***	
	(0.1513)	
age6574	1.3801***	
	(0.1996)	
age75plus	0.7516***	
	(0.1976)	
female	0.2752**	
	(0.1093)	
smoker	-0.0475	
	(0.1164)	
married	-0.1388	
	(0.1201)	
black	-0.5746***	
	(0.1295)	
amind	-0.0833	
	(0.5225)	
asian	0.0741	
	(0.2181)	
othrace	-0.3856	
	(0.2546)	
midwest	0.0745	
	(0.1535)	
south	-0.0753	
	(0.1459)	
west	-0.0753	
	(0.1415)	
lesshischool	-0.0932	
	(0.1251)	
collegeplus	0.1167	
	(0.1235)	
poor	0.0804	
	(0.1364)	
nearpoor	-0.0692	
	(0.1871)	

middleinc	-0.1116	
	(0.1573)	
highinc	0.0108	
	(0.2241)	
_bs_1		6.6961e+09***
		(1.4095e+09)
_bs_2		0.1731***
		(0.0340)
_cons	6.4237***	
	(0.1912)	
No. of Obs.	3772.0000	3772.0000
R-Squared		

	(1)	(2)
	outpatient	
	Coef./std.errors	Coef./std.errors
main		
overweight	0.0426	
	(0.0739)	
obese	0.0385	
	(0.0772)	
age3544	0.7420*	
	(0.3859)	
age4554	0.5591*	
	(0.3337)	
age5564	0.5462*	
	(0.3084)	
age6574	0.1453	
	(0.2927)	
age75plus	0.1920	
	(0.2965)	
female	-0.0721	
	(0.0619)	
smoker	-0.2203**	
	(0.1024)	
married	-0.1450**	
	(0.0662)	
black	-0.2549**	
	(0.1083)	
amind	-0.3217	
	(0.3166)	
asian	-0.4647***	
	(0.1628)	
othrace	-0.3868*	
	(0.2156)	
midwest	0.0233	
	(0.0958)	
south	-0.0255	
	(0.0859)	
west	0.0477	
	(0.0974)	
lesshischool	-0.3204***	
	(0.0933)	
collegeplus	0.0451	
	(0.0705)	
poor	-0.0295	
	(0.1175)	
nearpoor	-0.0872	
	(0.1504)	

middleinc	0.1655*	
	(0.0958)	
highinc	0.3041***	
	(0.0950)	
_bs_1		1.8843e+09
		(3.8331e+09)
_bs_2		0.0124
		(0.0251)
_cons	7.8290***	
	(0.3253)	
No. of Obs.	4547.0000	4547.0000
R-Squared		

	(1)	(2)
	outpatient	
	Coef./std.errors	Coef./std.errors
main		
overweight	0.2732**	
	(0.1136)	
obese	0.3974***	
	(0.1189)	
unins	-0.9566***	
	(0.1349)	
medicaid	0.0001	
	(0.1566)	
medicare	0.9734*	
	(0.5011)	
female	0.8228***	
	(0.0972)	
smoker	0.2134	
	(0.1394)	
married	0.2668**	
	(0.1102)	
black	-0.6211***	
	(0.1409)	
amind	-0.7030	
	(0.5390)	
asian	-0.0436	
	(0.1950)	
othrace	-0.0841	
	(0.2513)	
midwest	-0.1462	
	(0.1556)	
south	-0.3264**	
	(0.1414)	
west	0.0971	
	(0.1498)	
lesshischool	-0.2327	
	(0.1586)	
collegeplus	0.3211***	
	(0.1145)	
poor	-0.0273	
	(0.1922)	
nearpoor	-0.2732	
	(0.2569)	
middleinc	-0.3138*	
	(0.1632)	
highinc	-0.0604	
	(0.1680)	

_bs_1		3.8392e+09**
		(1.7756e+09)
_bs_2		0.0731**
		(0.0325)
_cons	6.1753***	
	(0.1990)	
No. of Obs.	6003.0000	6003.0000
R-Squared		

	(1)	(2)
	outpatient	
	Coef./std.errors	Coef./std.errors
main		
overweight	0.0779	
	(0.1326)	
obese	0.4582***	
	(0.1314)	
unins	-0.5075***	
	(0.1613)	
medicaid	0.0108	
	(0.2027)	
medicare	1.6019***	
	(0.3711)	
female	0.7064***	
	(0.1024)	
smoker	-0.2132	
	(0.1454)	
married	-0.2257**	
	(0.1147)	
black	-0.4649***	
	(0.1649)	
amind	0.2103	
	(0.5218)	
asian	-0.3517*	
	(0.2039)	
othrace	-0.2089	
	(0.3165)	
midwest	0.1593	
	(0.1711)	
south	0.0458	
	(0.1522)	
west	0.1935	
	(0.1630)	
lesshischool	-0.4745***	
	(0.1841)	
collegeplus	0.0779	
	(0.1331)	
poor	-0.0432	
	(0.2255)	
nearpoor	0.0040	
	(0.3131)	
middleinc	0.2751	
	(0.1878)	
highinc	0.6407***	
	(0.1836)	

_bs_1		8.2432e+08
		(1.4789e+09)
_bs_2		0.0188
		(0.0333)
_cons	6.2584***	
	(0.2698)	
No. of Obs.	3609.0000	3609.0000
R-Squared		

	(1)	(2)
	outpatient	
	Coef./std.errors	Coef./std.errors
main		
overweight	0.1240	
	(0.1673)	
obese	0.3253*	
	(0.1664)	
unins	-0.9530***	
	(0.2152)	
medicaid	-0.2383	
	(0.2613)	
medicare	1.2667***	
	(0.3387)	
female	0.4565***	
	(0.1340)	
smoker	-0.2534	
	(0.1827)	
married	-0.0520	
	(0.1542)	
black	-0.0828	
	(0.2085)	
amind	0.5862	
	(0.7113)	
asian	-0.3696	
	(0.2742)	
othrace	0.3207	
	(0.4313)	
midwest	-0.2544	
	(0.2192)	
south	-0.1071	
	(0.1939)	
west	-0.1650	
	(0.2084)	
lesshischool	-0.4512*	
	(0.2412)	
collegeplus	0.4859***	
	(0.1538)	
poor	-0.1215	
	(0.3087)	
nearpoor	-0.4420	
	(0.4456)	
middleinc	-0.2887	
	(0.2507)	
highinc	-0.2476	
	(0.2451)	

_bs_1		2.5172e+09
		(3.2886e+09)
_bs_2		0.0380
		(0.0500)
_cons	7.2417***	
	(0.3458)	
No. of Obs.	3652.0000	3652.0000
R-Squared		

	(1)	(2)
	outpatient	
	Coef./std.errors	Coef./std.errors
main		
overweight	0.0814	
	(0.1102)	
obese	0.3615***	
	(0.1125)	
unins	-0.1476	
	(0.1516)	
medicaid	0.1723	
	(0.1612)	
medicare	0.6900***	
	(0.1558)	
female	0.2237**	
	(0.0889)	
smoker	-0.0401	
	(0.1196)	
married	0.2396**	
	(0.0955)	
black	-0.0735	
	(0.1367)	
amind	-0.2792	
	(0.4199)	
asian	-0.4676**	
	(0.2144)	
othrace	-0.1725	
	(0.2849)	
midwest	0.0863	
	(0.1380)	
south	-0.0453	
	(0.1256)	
west	0.0950	
	(0.1380)	
lesshischool	-0.1523	
	(0.1519)	
collegeplus	0.3159***	
	(0.0973)	
poor	0.3571*	
	(0.1931)	
nearpoor	0.0472	
	(0.2723)	
middleinc	0.2758	
	(0.1680)	
highinc	0.5662***	
	(0.1639)	

_bs_1		2.3006e+09
		(3.3193e+09)
_bs_2		0.0263
		(0.0380)
_cons	6.6694***	
	(0.2215)	
No. of Obs.	3353.0000	3353.0000
R-Squared		

	(1)	(2)
	outpatient	
	Coef./std.errors	Coef./std.errors
main		
overweight	-0.0397	
	(0.1078)	
obese	0.0096	
	(0.1072)	
female	-0.1143	
	(0.0864)	
smoker	-0.1523	
	(0.1377)	
married	-0.2737***	
	(0.0942)	
black	-0.2027	
	(0.1587)	
amind	-0.2387	
	(0.5527)	
asian	-0.1711	
	(0.2175)	
othrace	-0.4024	
	(0.2864)	
midwest	0.1467	
	(0.1434)	
south	0.0346	
	(0.1297)	
west	0.0584	
	(0.1414)	
lesshischool	-0.2208	
	(0.1422)	
collegeplus	0.1014	
	(0.1017)	
poor	0.0825	
	(0.1972)	
nearpoor	0.1197	
	(0.2458)	
middleinc	0.2112	
	(0.1469)	
highinc	0.4618***	
	(0.1417)	
_bs_1		-1.0594e+09
		(3.1199e+09)
_bs_2		-0.0138
		(0.0404)
_cons	7.8640***	
	(0.2055)	

No. of Obs.	2309.0000	2309.0000
R-Squared		

	(1)	(2)
	outpatient	
	Coef./std.errors	Coef./std.errors
main		
overweight	0.1120	
	(0.1098)	
obese	0.0114	
	(0.1299)	
female	-0.1326	
	(0.1066)	
smoker	-0.4328	
	(0.2793)	
married	0.0125	
	(0.1114)	
black	-0.3315*	
	(0.1920)	
amind	-0.1824	
	(0.5791)	
asian	-0.8516***	
	(0.2539)	
othrace	-0.3824	
	(0.5896)	
midwest	-0.0316	
	(0.1447)	
south	-0.0780	
	(0.1314)	
west	0.0907	
	(0.1516)	
lesshischool	-0.4838***	
	(0.1445)	
collegeplus	-0.0459	
	(0.1109)	
poor	-0.0026	
	(0.1881)	
nearpoor	-0.3634	
	(0.2343)	
middleinc	-0.0368	
	(0.1461)	
highinc	-0.0242	
	(0.1479)	
_bs_1		2.3986e+09
		(3.0112e+09)
_bs_2		0.0427
		(0.0534)
_cons	8.2800***	
	(0.1885)	

No. of Obs.	1640.0000	1640.0000
R-Squared		

	(1)	(2)
	outpatient	
	Coef./std.errors	Coef./std.errors
main		
overweight	0.1353**	
	(0.0592)	
obese	0.2906***	
	(0.0616)	
age3544	0.3122***	
	(0.0789)	
age4554	0.6911***	
	(0.0766)	
age5564	0.9345***	
	(0.0768)	
age6574	1.1596***	
	(0.0810)	
age75plus	1.2920***	
	(0.0933)	
female	0.3503***	
	(0.0495)	
smoker	-0.0543	
	(0.0741)	
married	0.0335	
	(0.0529)	
black	-0.2975***	
	(0.0792)	
amind	-0.1784	
	(0.3048)	
asian	-0.3262***	
	(0.1051)	
othrace	-0.1928	
	(0.1524)	
midwest	0.0471	
	(0.0783)	
south	-0.0669	
	(0.0703)	
west	0.1164	
	(0.0765)	
lesshischool	-0.2284***	
	(0.0862)	
collegeplus	0.2238***	
	(0.0573)	
poor	0.1383	
	(0.1098)	
nearpoor	-0.0767	
	(0.1494)	

middleinc	0.0172	
	(0.0870)	
highinc	0.1623*	
	(0.0849)	
_bs_1		1.5871e+10*
		(8.2535e+09)
_bs_2		0.0432*
		(0.0221)
_cons	6.3308***	
	(0.1194)	
No. of Obs.	17196.0000	17196.0000
R-Squared		

	(1)	(2)
	outpatient	
	Coef./std.errors	Coef./std.errors
main		
overweight	0.0914	
	(0.0702)	
obese	0.2788***	
	(0.0743)	
age3544	0.2914***	
	(0.0890)	
age4554	0.6493***	
	(0.0871)	
age5564	0.9161***	
	(0.0886)	
age6574	1.1598***	
	(0.1097)	
age75plus	1.4980***	
	(0.1332)	
female	0.4508***	
	(0.0590)	
smoker	-0.0250	
	(0.0949)	
married	0.1340**	
	(0.0632)	
black	-0.1998**	
	(0.1005)	
amind	-0.0004	
	(0.4249)	
asian	-0.4189***	
	(0.1199)	
othrace	-0.0554	
	(0.1835)	
midwest	0.0063	
	(0.0931)	
south	-0.1456*	
	(0.0845)	
west	0.0890	
	(0.0924)	
lesshischool	-0.3231***	
	(0.1217)	
collegeplus	0.3017***	
	(0.0682)	
poor	0.0794	
	(0.1856)	
nearpoor	-0.0864	
	(0.2517)	

middleinc	0.0703	
	(0.1182)	
highinc	0.1840	
	(0.1152)	
_bs_1		7.7127e+09
		(6.7238e+09)
_bs_2		0.0296
		(0.0256)
_cons	6.1695***	
	(0.1542)	
No. of Obs.	11222.0000	11222.0000
R-Squared		

	(1)	(2)
	outpatient	
	Coef./std.errors	Coef./std.errors
main		
overweight	0.4025***	
	(0.1269)	
obese	0.5201***	
	(0.1214)	
age3544	0.2521*	
	(0.1517)	
age4554	0.6434***	
	(0.1531)	
age5564	0.9584***	
	(0.1513)	
age6574	1.3801***	
	(0.1996)	
age75plus	0.7516***	
	(0.1976)	
female	0.2752**	
	(0.1093)	
smoker	-0.0475	
	(0.1164)	
married	-0.1388	
	(0.1201)	
black	-0.5746***	
	(0.1295)	
amind	-0.0833	
	(0.5225)	
asian	0.0741	
	(0.2181)	
othrace	-0.3856	
	(0.2546)	
midwest	0.0745	
	(0.1535)	
south	-0.0753	
	(0.1459)	
west	-0.0753	
	(0.1415)	
lesshischool	-0.0932	
	(0.1251)	
collegeplus	0.1167	
	(0.1235)	
poor	0.0804	
	(0.1364)	
nearpoor	-0.0692	
	(0.1871)	

middleinc	-0.1116	
	(0.1573)	
highinc	0.0108	
	(0.2241)	
_bs_1		4.2225e+09**
		(1.6553e+09)
_bs_2		0.1091***
		(0.0408)
_cons	6.4237***	
	(0.1912)	
No. of Obs.	3772.0000	3772.0000
R-Squared		

	(1)	(2)
	outpatient	
	Coef./std.errors	Coef./std.errors
main		
overweight	0.0426	
	(0.0739)	
obese	0.0385	
	(0.0772)	
age3544	0.7420*	
	(0.3859)	
age4554	0.5591*	
	(0.3337)	
age5564	0.5462*	
	(0.3084)	
age6574	0.1453	
	(0.2927)	
age75plus	0.1920	
	(0.2965)	
female	-0.0721	
	(0.0619)	
smoker	-0.2203**	
	(0.1024)	
married	-0.1450**	
	(0.0662)	
black	-0.2549**	
	(0.1083)	
amind	-0.3217	
	(0.3166)	
asian	-0.4647***	
	(0.1628)	
othrace	-0.3868*	
	(0.2156)	
midwest	0.0233	
	(0.0958)	
south	-0.0255	
	(0.0859)	
west	0.0477	
	(0.0974)	
lesshischool	-0.3204***	
	(0.0933)	
collegeplus	0.0451	
	(0.0705)	
poor	-0.0295	
	(0.1175)	
nearpoor	-0.0872	
	(0.1504)	

middleinc	0.1655*	
	(0.0958)	
highinc	0.3041***	
	(0.0950)	
_bs_1		2.2651e+09
		(4.4376e+09)
_bs_2		0.0149
		(0.0293)
_cons	7.8290***	
	(0.3253)	
No. of Obs.	4547.0000	4547.0000
R-Squared		

	(1)	(2)
	totalexp	
	Coef./std.errors	Coef./std.errors
main		
overweight	0.0956	
	(0.1176)	
obese	0.2103*	
	(0.1273)	
unins	-0.4908***	
	(0.1411)	
medicaid	0.4073**	
	(0.1658)	
medicare	1.2989**	
	(0.5255)	
female	0.6918***	
	(0.1022)	
smoker	0.3715**	
	(0.1531)	
married	0.5643***	
	(0.1183)	
black	-0.3274**	
	(0.1547)	
amind	-0.4447	
	(0.5644)	
asian	-0.3703*	
	(0.1961)	
othrace	-0.1723	
	(0.2584)	
midwest	-0.1059	
	(0.1653)	
south	-0.2135	
	(0.1522)	
west	-0.1463	
	(0.1577)	
lesshischool	-0.4055**	
	(0.1612)	
collegeplus	0.0731	
	(0.1275)	
poor	0.3173	
	(0.2029)	
nearpoor	-0.3457	
	(0.2722)	
middleinc	-0.1059	
	(0.1738)	
highinc	-0.0519	
	(0.1777)	

_bs_1		3.5093e+09
		(4.3316e+09)
_bs_2		0.0257
		(0.0317)
_cons	7.1212***	
	(0.2195)	
No. of Obs.	6003.0000	6003.0000
R-Squared		

	(1)	(2)
	totalexp	
	Coef./std.errors	Coef./std.errors
main		
overweight	-0.1197	
	(0.1336)	
obese	0.3724***	
	(0.1358)	
unins	-0.6085***	
	(0.1544)	
medicaid	0.0658	
	(0.1901)	
medicare	1.8221***	
	(0.3786)	
female	0.5338***	
	(0.1059)	
smoker	0.2534	
	(0.1559)	
married	-0.2070*	
	(0.1199)	
black	-0.5280***	
	(0.1648)	
amind	0.4618	
	(0.5239)	
asian	-0.4526**	
	(0.2070)	
othrace	-0.0551	
	(0.3230)	
midwest	0.1096	
	(0.1781)	
south	0.3274**	
	(0.1587)	
west	0.2036	
	(0.1648)	
lesshischool	-0.4674**	
	(0.1920)	
collegeplus	-0.0951	
	(0.1345)	
poor	0.5171**	
	(0.2369)	
nearpoor	0.5483*	
	(0.3104)	
middleinc	0.3065*	
	(0.1817)	
highinc	0.5288***	
	(0.1799)	

_bs_1		-3.2686e+09
		(4.2035e+09)
_bs_2		-0.0280
		(0.0349)
_cons	7.3118***	
	(0.2638)	
No. of Obs.	3609.0000	3609.0000
R-Squared		

	(1)	(2)
	totalexp	
	Coef./std.errors	Coef./std.errors
main		
overweight	0.1763	
	(0.1391)	
obese	0.3852***	
	(0.1378)	
unins	-0.8727***	
	(0.1836)	
medicaid	-0.1129	
	(0.2237)	
medicare	1.2227***	
	(0.2721)	
female	0.3174***	
	(0.1095)	
smoker	0.0685	
	(0.1514)	
married	-0.1791	
	(0.1275)	
black	-0.1345	
	(0.1731)	
amind	0.4063	
	(0.5916)	
asian	-0.2070	
	(0.2241)	
othrace	0.5706	
	(0.3522)	
midwest	-0.0939	
	(0.1781)	
south	-0.0334	
	(0.1618)	
west	-0.2616	
	(0.1731)	
lesshischool	-0.1003	
	(0.1937)	
collegeplus	0.3819***	
	(0.1268)	
poor	0.0263	
	(0.2537)	
nearpoor	0.0448	
	(0.3662)	
middleinc	-0.4198**	
	(0.2053)	
highinc	-0.3395*	
	(0.2034)	

_bs_1		8.8032e+09
		(7.7425e+09)
_bs_2		0.0521
		(0.0447)
_cons	8.2673***	
	(0.2876)	
No. of Obs.	3652.0000	3652.0000
R-Squared		

	(1)	(2)
	totalexp	
	Coef./std.errors	Coef./std.errors
main		
overweight	-0.1478	
	(0.1044)	
obese	0.1950*	
	(0.1060)	
unins	-0.0645	
	(0.1452)	
medicaid	0.5003***	
	(0.1590)	
medicare	0.7979***	
	(0.1511)	
female	0.0764	
	(0.0841)	
smoker	-0.0492	
	(0.1158)	
married	0.1435	
	(0.0944)	
black	0.0114	
	(0.1288)	
amind	-0.6729*	
	(0.3999)	
asian	-0.6642***	
	(0.2054)	
othrace	0.0039	
	(0.2746)	
midwest	0.0963	
	(0.1326)	
south	0.0918	
	(0.1207)	
west	0.0751	
	(0.1329)	
lesshischool	-0.1905	
	(0.1450)	
collegeplus	0.2212**	
	(0.0935)	
poor	0.1919	
	(0.1880)	
nearpoor	-0.2414	
	(0.2600)	
middleinc	0.0712	
	(0.1583)	
highinc	0.0298	
	(0.1536)	

_bs_1		-1.1566e+10
		(1.0476e+10)
_bs_2		-0.0484
		(0.0433)
_cons	8.2674***	
	(0.2118)	
No. of Obs.	3353.0000	3353.0000
R-Squared		

	(1)	(2)
	totalexp	
	Coef./std.errors	Coef./std.errors
main		
overweight	0.0778	
	(0.1040)	
obese	0.2716***	
	(0.1039)	
female	0.0163	
	(0.0854)	
smoker	-0.1473	
	(0.1314)	
married	-0.3581***	
	(0.0933)	
black	-0.1546	
	(0.1484)	
amind	0.3243	
	(0.5368)	
asian	-0.1820	
	(0.2075)	
othrace	-0.0190	
	(0.2746)	
midwest	0.2158	
	(0.1356)	
south	0.1699	
	(0.1230)	
west	-0.0560	
	(0.1331)	
lesshischool	-0.0552	
	(0.1387)	
collegeplus	-0.0357	
	(0.0994)	
poor	0.1475	
	(0.1911)	
nearpoor	0.1831	
	(0.2390)	
middleinc	0.0770	
	(0.1401)	
highinc	0.2532*	
	(0.1391)	
_bs_1		5.7951e+09
		(9.5938e+09)
_bs_2		0.0247
		(0.0403)
_cons	8.9751***	
	(0.1957)	

No. of Obs.	2309.0000	2309.0000
R-Squared		

	(1)	(2)
	totalexp	
	Coef./std.errors	Coef./std.errors
main		
overweight	0.0166	
	(0.0866)	
obese	0.2884***	
	(0.1023)	
female	-0.0630	
	(0.0826)	
smoker	-0.4090*	
	(0.2209)	
married	0.0170	
	(0.0874)	
black	-0.2261	
	(0.1484)	
amind	0.1736	
	(0.4574)	
asian	-0.6627***	
	(0.2030)	
othrace	-0.6672	
	(0.4626)	
midwest	-0.0340	
	(0.1154)	
south	-0.0291	
	(0.1040)	
west	0.2125*	
	(0.1210)	
lesshischool	0.0003	
	(0.1139)	
collegeplus	-0.1261	
	(0.0879)	
poor	0.0300	
	(0.1455)	
nearpoor	-0.0920	
	(0.1858)	
middleinc	-0.1334	
	(0.1139)	
highinc	-0.0499	
	(0.1130)	
_bs_1		1.0207e+09
		(6.7657e+09)
_bs_2		0.0059
		(0.0384)
_cons	9.2590***	
	(0.1568)	

No. of Obs.	1640.0000	1640.0000
R-Squared		

	(1)	(2)
	totalexp	
	Coef./std.errors	Coef./std.errors
main		
overweight	0.0518	
	(0.0522)	
obese	0.2605***	
	(0.0543)	
age3544	0.3983***	
	(0.0696)	
age4554	0.7507***	
	(0.0678)	
age5564	1.0503***	
	(0.0674)	
age6574	1.3806***	
	(0.0716)	
age75plus	1.4767***	
	(0.0821)	
female	0.3347***	
	(0.0435)	
smoker	0.1120*	
	(0.0664)	
married	0.0203	
	(0.0471)	
black	-0.2499***	
	(0.0698)	
amind	0.0549	
	(0.2690)	
asian	-0.4700***	
	(0.0917)	
othrace	-0.0115	
	(0.1350)	
midwest	0.0142	
	(0.0690)	
south	-0.0073	
	(0.0625)	
west	-0.0265	
	(0.0673)	
lesshischool	-0.0838	
	(0.0756)	
collegeplus	0.1145**	
	(0.0507)	
poor	0.4044***	
	(0.0967)	
nearpoor	0.0119	
	(0.1320)	

middleinc	-0.0903	
	(0.0769)	
highinc	-0.1049	
	(0.0750)	
_bs_1		1.6680e+10
		(2.4777e+10)
_bs_2		0.0163
		(0.0241)
_cons	7.4595***	
	(0.1027)	
No. of Obs.	17196.0000	17196.0000
R-Squared		

	(1)	(2)
	totalexp	
	Coef./std.errors	Coef./std.errors
main		
overweight	0.0328	
	(0.0652)	
obese	0.2645***	
	(0.0686)	
age3544	0.3466***	
	(0.0826)	
age4554	0.7116***	
	(0.0807)	
age5564	1.0338***	
	(0.0815)	
age6574	1.3575***	
	(0.1016)	
age75plus	1.6255***	
	(0.1231)	
female	0.4312***	
	(0.0548)	
smoker	0.1853**	
	(0.0902)	
married	0.1397**	
	(0.0584)	
black	-0.2332**	
	(0.0933)	
amind	0.2200	
	(0.3944)	
asian	-0.4313***	
	(0.1115)	
othrace	0.0996	
	(0.1714)	
midwest	-0.0217	
	(0.0863)	
south	-0.0140	
	(0.0788)	
west	-0.0343	
	(0.0857)	
lesshischool	-0.0597	
	(0.1132)	
collegeplus	0.2129***	
	(0.0634)	
poor	0.2228	
	(0.1738)	
nearpoor	0.0693	
	(0.2328)	

middleinc	-0.0613	
	(0.1097)	
highinc	-0.0854	
	(0.1068)	
_bs_1		6.9209e+09
		(1.5849e+10)
_bs_2		0.0105
		(0.0242)
_cons	7.2319***	
	(0.1433)	
No. of Obs.	11222.0000	11222.0000
R-Squared		

	(1)	(2)
	totalexp	
	Coef./std.errors	Coef./std.errors
main		
overweight	0.1459	
	(0.1223)	
obese	0.2648**	
	(0.1196)	
age3544	0.3796**	
	(0.1476)	
age4554	0.7129***	
	(0.1486)	
age5564	1.1989***	
	(0.1469)	
age6574	1.6442***	
	(0.1931)	
age75plus	1.4095***	
	(0.1948)	
female	0.0864	
	(0.1034)	
smoker	-0.0103	
	(0.1149)	
married	-0.2014*	
	(0.1156)	
black	-0.4125***	
	(0.1255)	
amind	-0.6125	
	(0.5036)	
asian	-0.5264**	
	(0.2065)	
othrace	-0.0388	
	(0.2459)	
midwest	0.0388	
	(0.1485)	
south	0.0692	
	(0.1425)	
west	-0.3435**	
	(0.1376)	
lesshischool	-0.1840	
	(0.1211)	
collegeplus	-0.1012	
	(0.1207)	
poor	0.3887***	
	(0.1305)	
nearpoor	-0.1142	
	(0.1788)	

middleinc	0.0508	
	(0.1515)	
highinc	0.2635	
	(0.2143)	
_bs_1		7.2049e+09
		(8.4683e+09)
_bs_2		0.0421
		(0.0487)
_cons	7.9736***	
	(0.1739)	
No. of Obs.	3772.0000	3772.0000
R-Squared		

	(1)	(2)
	totalexp	
	Coef./std.errors	Coef./std.errors
main		
overweight	0.0525	
	(0.0649)	
obese	0.2427***	
	(0.0672)	
age3544	0.7426**	
	(0.3408)	
age4554	0.4260	
	(0.2927)	
age5564	0.3708	
	(0.2706)	
age6574	-0.0144	
	(0.2578)	
age75plus	0.0292	
	(0.2605)	
female	0.0193	
	(0.0550)	
smoker	-0.0896	
	(0.0920)	
married	-0.1957***	
	(0.0594)	
black	-0.1140	
	(0.0940)	
amind	0.0023	
	(0.2793)	
asian	-0.3765***	
	(0.1431)	
othrace	-0.1767	
	(0.1891)	
midwest	0.0645	
	(0.0840)	
south	0.0586	
	(0.0758)	
west	0.0274	
	(0.0851)	
lesshischool	-0.0003	
	(0.0829)	
collegeplus	-0.0374	
	(0.0619)	
poor	0.0916	
	(0.1034)	
nearpoor	-0.0596	
	(0.1333)	

middleinc	-0.0006	
	(0.0835)	
highinc	0.1151	
	(0.0833)	
_bs_1		8.1994e+09
		(1.2280e+10)
_bs_2		0.0167
		(0.0250)
_cons	9.0896***	
	(0.2845)	
No. of Obs.	4547.0000	4547.0000
R-Squared		
